# PD-1/PD-L1 inhibitors combined with chemotherapy as first-line treatment for advanced biliary tract cancer: a systematic review and meta-analysis

**DOI:** 10.3389/fmed.2026.1916331

**Published:** 2026-09-03

**Authors:** Hongye Geng, Chengyuan Lu, Shuling Wang, Lijuan Tian

**Affiliations:** School of Business Administration, Shenyang Pharmaceutical University, Shenyang, China

**Keywords:** biliary tract cancer, chemotherapy, meta-analysis, PD-1/PD-L1 inhibitors, randomized controlled trial, retrospective study

## Abstract

**Objective:**

To systematically evaluate the efficacy and safety of PD-1/PD-L1 inhibitors plus chemotherapy as first-line treatment for advanced biliary tract cancer (BTC).

**Methods:**

PubMed, Embase, Cochrane Library, Web of Science, CNKI, Wanfang, and VIP databases were searched for RCTs and retrospective studies. Meta-analysis was performed using R 4.6.1 software with the meta (v8.5–0) and metafor (v5.0–1) packages. The full R code and datasets are provided in the supplementary material to ensure reproducibility.

**Results:**

Part I included 5 RCTs with 2,000 patients; of these, only 2 phase III RCTs (TOPAZ-1 and KEYNOTE-966, *n* = 1,754) reported OS and PFS data, while all 5 RCTs reported ORR and DCR data; Part II included 6 retrospective studies with 1,253 patients. RCT meta-analysis showed: OS was significantly improved (HR = 0.80, 95% CI: 0.72–0.89, *p* < 0.001); PFS was also significantly improved (HR = 0.81, 95% CI: 0.71–0.93, *p* = 0.002); ORR was significantly higher (RR = 1.38, 95% CI: 1.02–1.87, *p* = 0.037). Retrospective study meta-analysis showed: PFS was significantly superior (HR = 0.52, 95% CI: 0.43–0.63, *p* < 0.001); OS was significantly prolonged (HR = 0.61, 95% CI: 0.42–0.87, *p* = 0.017); ORR (RR = 1.97, 95% CI: 1.43–2.71, *p* = 0.004) and DCR (RR = 1.26, 95% CI: 1.09–1.45, *p* = 0.014) were also significantly higher. Neither part showed significantly increased severe adverse events.

**Conclusion:**

PD-1/PD-L1 inhibitors plus chemotherapy significantly improves survival outcomes and objective response rate in advanced BTC without increasing severe adverse event risk.

**Systematic review registration:**

PROSPERO. Unique Identifier: CRD420261401842 URL: https://www.crd.york.ac.uk/prospero/display_record.php?

## 1 Introduction

Biliary tract cancer (BTC) is a group of malignancies originating from the epithelial cells of the biliary system, including intrahepatic cholangiocarcinoma (iCCA), extrahepatic cholangiocarcinoma, and gallbladder cancer (GBC), accounting for approximately 3% of all gastrointestinal malignancies (1). BTC has an extremely poor prognosis, and most patients present with advanced disease at diagnosis, losing the opportunity for surgical resection. For a long time, the gemcitabine plus cisplatin (GP) regimen has been the standard first-line treatment for advanced BTC, but the median overall survival (OS) is only approximately 11.7 months, indicating limited clinical efficacy (2).

In recent years, immune checkpoint inhibitors (ICIs) have achieved breakthrough progress in various solid tumors by blocking the programmed cell death receptor-1 (PD-1) and its ligand (PD-L1) pathway to restore T-cell anti-tumor immune responses (3). In 2022, the TOPAZ-1 study, with its phase 3, double-blind, placebo-controlled design evaluating durvalumab plus gemcitabine/cisplatin versus placebo plus chemotherapy, stratified by disease status and primary tumor location (4). First demonstrated that durvalumab combined with GP significantly improved OS compared with chemotherapy alone in patients with advanced BTC (5), and the KEYNOTE-966 study further validated the clinical benefit of pembrolizumab combined with GP (6), with its phase 3 design (7, 8) including pembrolizumab 200 mg every 3 weeks plus gemcitabine/cisplatin, stratified by region, stage, and tumor origin, with dual primary endpoints of PFS and OS. Other combination strategies are also under active investigation, including an ongoing exploratory trial of anti-PD-1 antibody plus chemotherapy for unresectable iCCA (9), gemcitabine plus durvalumab in cisplatin-ineligible patients with unresectable locally advanced or metastatic BTC (10), and a phase II/III trial of gemcitabine plus cisplatin with or without bintrafusp alfa (M7824) as first-line treatment of BTC (11). However, the strict inclusion and exclusion criteria of randomized controlled trials (RCTs) limit the generalizability of their conclusions to real-world populations. Retrospective studies, as an important complement to RCTs, can reflect the efficacy and safety in real-world clinical practice. This study comprehensively searched the literature for both RCTs and retrospective studies, employing meta-analysis methods to comprehensively evaluate the efficacy and safety of PD-1/PD-L1 inhibitors combined with chemotherapy as first-line treatment for advanced BTC.

## 2 Materials and methods

This systematic review was conducted according to the PRISMA 2020 statement and has been registered in PROSPERO (registration number: CRD420261401842).

### 2.1 Inclusion and exclusion criteria

#### 2.1.1 Randomized controlled trials

Inclusion criteria: (1) study design: RCTs, regardless of blinding status; (2) participants: Patients with histopathologically confirmed advanced BTC (unresectable, recurrent, or metastatic); (3) intervention: The experimental group received PD-1/PD-L1 inhibitors combined with chemotherapy, and the control group received chemotherapy alone; (4) outcome measures: OS, PFS, ORR, DCR, or incidence of adverse events.

Exclusion criteria: (1) non-RCT studies; (2) control groups containing ICIs; (3) duplicate publications; (4) inability to extract valid data.

Languages: English and Chinese.

#### 2.1.2 Retrospective studies

Inclusion criteria: (1) study design: Retrospective studies; (2) participants: Patients with histopathologically confirmed advanced BTC; (3) intervention: The experimental group received PD-1/PD-L1 inhibitors combined with gemcitabine-based chemotherapy, and the control group received gemcitabine-based chemotherapy alone; (4) availability of usable survival outcome data.

Exclusion criteria: (1) prospective studies, RCTs, or case reports; (2) control group containing ICIs; (3) duplicate publications; (4) inability to extract valid data.

### 2.2 Literature search strategy

A computerized search was conducted across PubMed, Embase, Web of Science, Cochrane Library, CNKI, WanFang, and VIP databases from inception to June 1, 2026. R 4.6.1 software with the meta (v8.5–0) and metafor (v5.0–1) packages was used for meta-analysis. All R code and analytical datasets are provided in the Supplementary Material to ensure full reproducibility.

For RCTs, time-to-event variables were analyzed using hazard ratios (HRs) with 95% confidence intervals (CIs), and dichotomous variables were analyzed using risk ratios (RRs) with 95% CIs. Heterogeneity was assessed using the *Q* test and the *i*^2^ statistic. A fixed-effects model was applied when *i*^2^ < 50%; otherwise, a random-effects model was used. Given that the PFS analysis for RCTs showed *i*^2^ = 29.8% (*p* = 0.23), below the 50% threshold, the fixed-effects model was selected for the primary PFS pooled estimate per protocol. Sensitivity analysis was performed using the leave-one-out method, and funnel plots were used to assess publication bias. *p* < 0.05 was considered statistically significant.

For retrospective studies, effect sizes were expressed as HRs with 95% CIs for survival outcomes and RRs with 95% CIs for dichotomous outcomes. Heterogeneity was assessed using the *Q* test and the *i*^2^ statistic. A fixed-effects model was applied when *i*^2^ < 50%, and a random-effects model when *i*^2^ ≥ 50%. Sensitivity analysis was performed using the leave-one-out method. Publication bias was assessed using funnel plots and the trim-and-fill method. Additional sensitivity analyses were conducted to assess the impact of potential patient cohort overlap: Zhao 2021 and Zhao 2025 were both conducted at the same center in China, raising the possibility of overlapping patient populations. We therefore performed scenario analyses excluding each study individually and both studies simultaneously, with results presented in Supplementary Figure S1. Subgroup analyses were conducted by tumor type, geographic region, and study design. ICI type (PD-1 inhibitor vs. PD-L1 inhibitor), and chemotherapy backbone. Interaction tests for subgroup differences were performed using meta-regression (rma.uni function in metafor).

The search strategy is illustrated using RCTs as an example. The search strategy combined medical subject headings (MeSH) and free-text keywords. Using PubMed as an example, the relevant search terms were: (“Biliary tract neoplasms”[MeSH] or “Bile duct neoplasms”[MeSH] or “Gallbladder neoplasms”[MeSH] or “Cholangiocarcinoma”[MeSH] or biliary tract cancer[tiab] or bile duct cancer[tiab] or gallbladder cancer[tiab] or cholangiocarcinoma[tiab] or btc[tiab]) and (“Immune checkpoint inhibitors”[MeSH] or “PD-1”[tiab] or “PD-L1”[tiab] or “PD1”[tiab] or “PDL1”[tiab] or “Programmed cell death 1”[tiab] or “Programmed death ligand 1”[tiab] or pembrolizumab[tiab] or nivolumab[tiab] or atezolizumab[tiab] or durvalumab[tiab] or sintilimab[tiab] or camrelizumab[tiab] or tislelizumab[tiab] or toripalimab[tiab] or sugemalimab[tiab]) and (chemotherapy[tiab] or chemoradiotherapy[tiab] or “Combination therapy”[tiab] or “Combined therapy”[tiab] or “Antineoplastic combined chemotherapy protocols”[MeSH]).

## 3 Results

### 3.1 Literature screening and study characteristics

**Figure 1:**
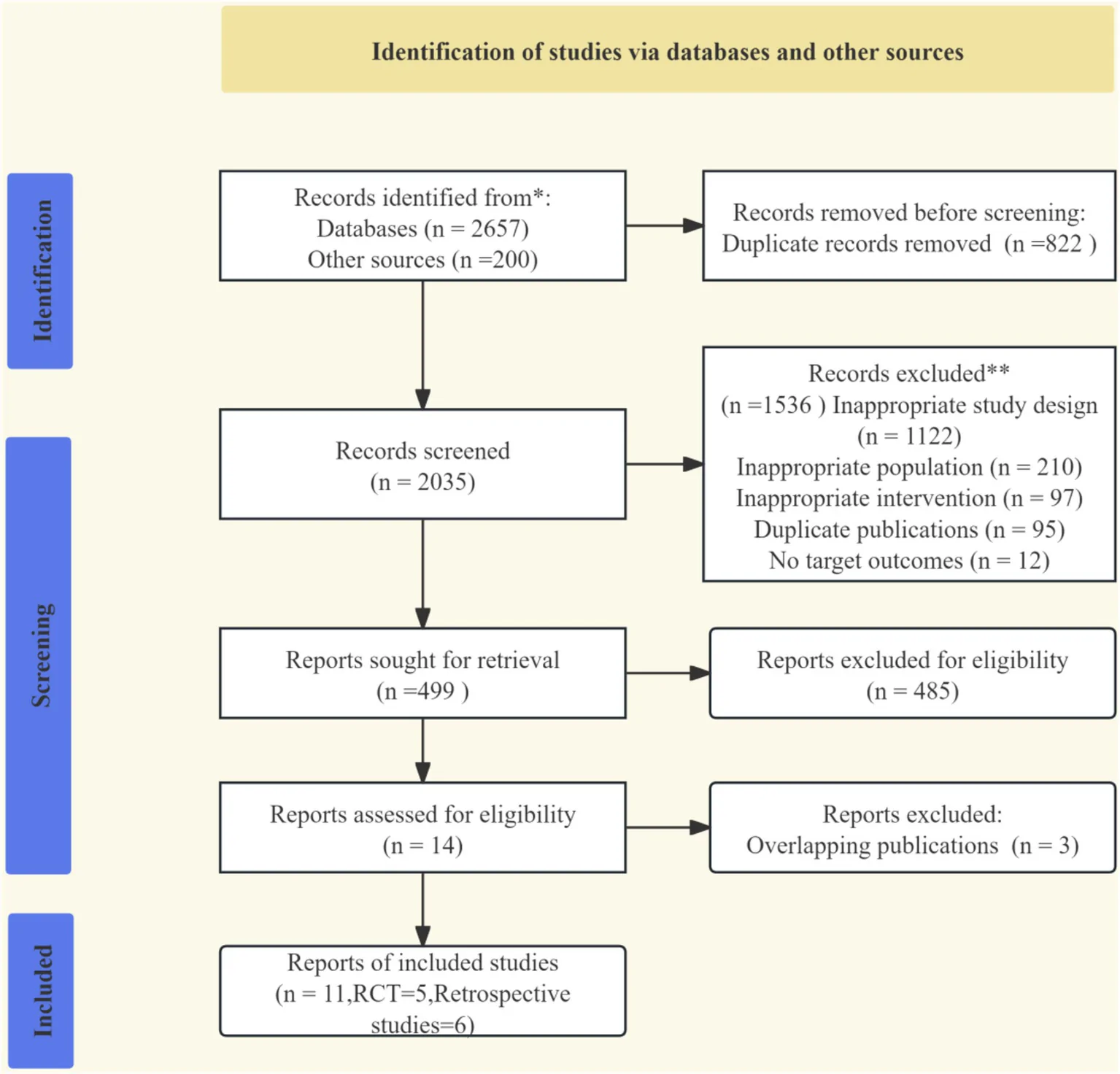
Prisma flowchart of study selection.

A total of 2,657 relevant articles were initially retrieved. After deduplication, screening, and full-text evaluation, 5 RCTs (4–6, 8, 12–14) and 6 retrospective studies (15–20) were finally included (PRISMA flowchart, see Figure 1).

### 3.2 Randomized controlled trials (RCTs)

The included RCTs comprised a total of 2,000 patients. Among the 5 RCTs, 2 were international multicenter phase III studies (TOPAZ-1 and KEYNOTE-966), and 3 were single-center small-sample studies from China. Both phase III studies employed randomized, double-blind designs with a low risk of bias. Regarding the RoB2 assessment: the 3 single-center RCTs did not report detailed information on allocation concealment and blinding implementation; specifically, the sequence generation domain was rated as ‘unclear’ for Cui 2023 and Li 2023 due to insufficient reporting of randomization methods, while Deng 2023 was rated as ‘low risk’ for sequence generation but ‘unclear’ for allocation concealment and blinding of participants/personnel. The basic characteristics of the included studies are shown in Table 1.

**Table 1:** Characteristics of included RCTs.

Study	Publication year	Country	Study design	Tumor type	Experimental group (*n*)	Control group (*n*)	Total sample size
TOPAZ-1	2022	Multinational (17 countries)	Multicenter, double-blind, placebo-controlled RCT	BTC (biliary tract cancer)	Durvalumab + GP (*n* = 341)	Placebo + GP (*n* = 344)	685
KEYNOTE-966	2023	Multinational (Global)	Multicenter, double-blind, placebo-controlled RCT	BTC (biliary tract cancer)	Pembrolizumab + GP (*n* = 533)	Placebo + GP (*n* = 536)	1,069
Cui 2023	2023	China	Single-center, open-label RCT	BTC (biliary tract cancer)	Nivolumab + GEMOX (*n* = 50)	GEMOX (*n* = 50)	100
Deng 2023	2023	China	Single-center, open-label RCT	iCCA (intrahepatic cholangiocarcinoma)	Pembrolizumab + XELOX (*n* = 40)	XELOX (*n* = 40)	80
Li 2023	2023	China	Single-center, open-label RCT	BTC (biliary tract cancer)	Sintilimab + GC (*n* = 33)	GC (*n* = 33)	66

#### 3.2.1 Retrospective studies

The included retrospective studies comprised a total of 1,253 patients. Among the 6 studies, 5 were single-center studies from China and 1 was a multicenter study from Italy. Regarding tumor types, 4 studies included mixed BTC populations, 1 included only iCCA, and 1 included only GBC. Notably, Zhao 2021 and Zhao 2025 were both conducted at the same institution in China, raising the possibility of partial patient cohort overlap. We assessed the robustness of pooled estimates through sensitivity analyses (see Supplementary Figure S1). Quality assessment was performed using the ROBINS-I tool, and all studies were rated as having a moderate risk of bias. The basic characteristics of the included studies are shown in Table 2.

**Table 2:** Characteristics of included retrospective studies.

Study	Publication year	Country	Study design	Tumor type	Experimental group (*n*)	Control group (*n*)	Total sample size
Zhao 2021	2021	China	Single-center retrospective study	Mixed BTC	ICI + chemotherapy (*n* = 45)	Chemotherapy (*n* = 45)	90
Lu 2024	2024	China	Single-center retrospective study	Mixed BTC	Durvalumab + chemotherapy (*n* = 93)	Chemotherapy (*n* = 93)	186
Madzikatire 2024	2024	China	Single-center retrospective study	iCCA	Anti-PD-1/PD-L1 + chemotherapy (*n* = 51)	Chemotherapy (*n* = 30)	81
Rimini 2024	2024	Italy	Multicenter retrospective study	Mixed BTC	Durvalumab + GP (*n* = 350)	GP (*n* = 213)	563
Zhang 2025	2025	China	Single-center retrospective study	GBC	Tislelizumab + GEMOX (*n* = 62)	GEMOX (*n* = 88)	150
Zhao 2025	2025	China	Single-center retrospective study	Mixed BTC	Camrelizumab + chemotherapy (*n* = 93)	Chemotherapy (*n* = 90)	183

### 3.3 Overall survival (OS)

#### 3.3.1 Randomized controlled trials (RCTs)

**Figure 2:**
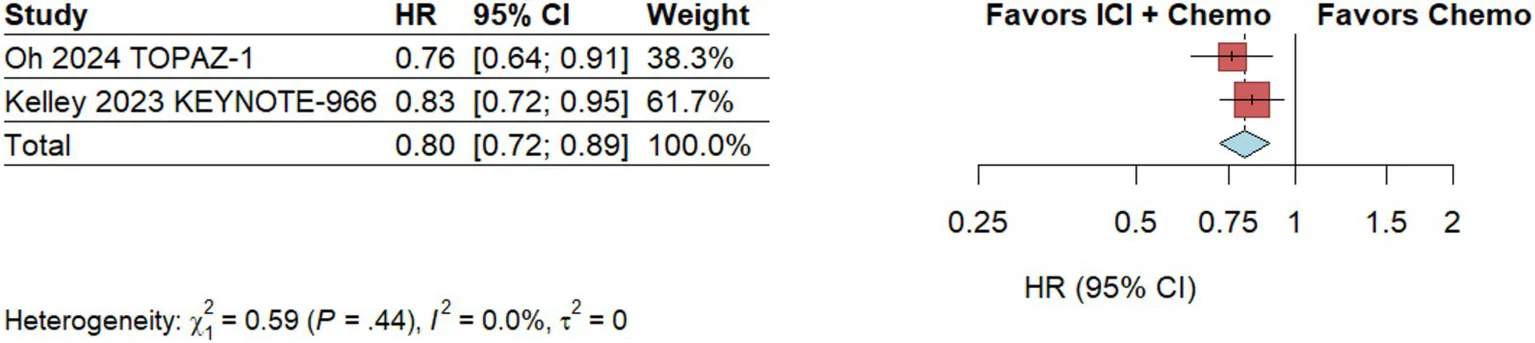
Forest plot of OS for RCTs.

Two phase III RCTs (TOPAZ-1 and KEYNOTE-966) reported OS data, encompassing a total of 1,754 patients; the remaining 3 single-center RCTs did not report OS and were not included in this pooling. Meta-analysis results showed that the combination group had significantly better OS than the chemotherapy-alone group (HR = 0.80, 95% CI: 0.72–0.89, *p* < 0.001), with heterogeneity *i*^2^ = 0%, *p* = 0.44, using a fixed-effects model (Figure 2). This result indicates that PD-1/PD-L1 inhibitors combined with chemotherapy can reduce the risk of death by approximately 20% compared with chemotherapy alone, and the results from two large phase III studies (TOPAZ-1: HR = 0.76; KEYNOTE-966: HR = 0.83) were highly consistent, with zero heterogeneity, enhancing the reliability of the conclusion.

#### 3.3.2 Retrospective studies

**Figure 3:**
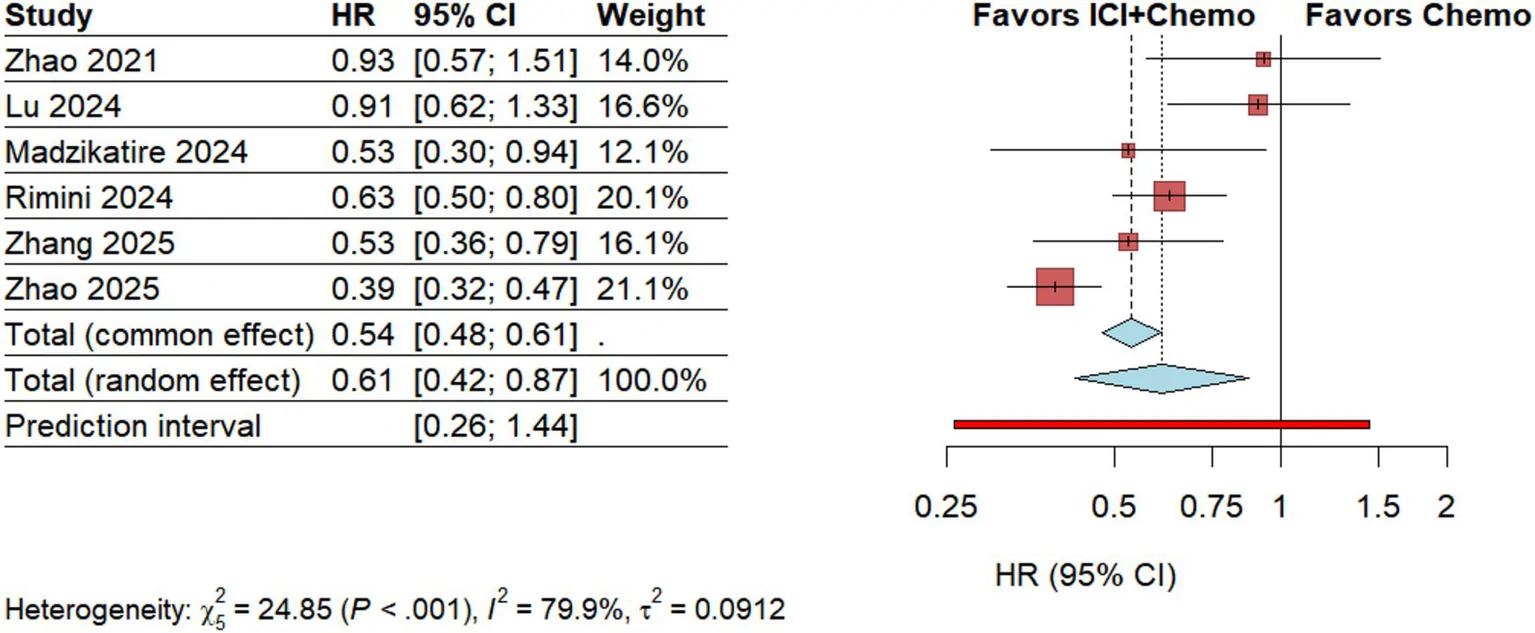
Forest plot of OS for retrospective studies.

All 6 retrospective studies reported OS data. Meta-analysis results showed that the combination group had significantly better OS than the chemotherapy group (HR = 0.61, 95% CI: 0.42–0.87, *p* = 0.017), with high heterogeneity (*i*^2^ = 79.9%, *p* < 0.001) (Figure 3).

### 3.4 Progression-free survival (PFS)

#### 3.4.1 Randomized controlled trials (RCTs)

**Figure 4:**
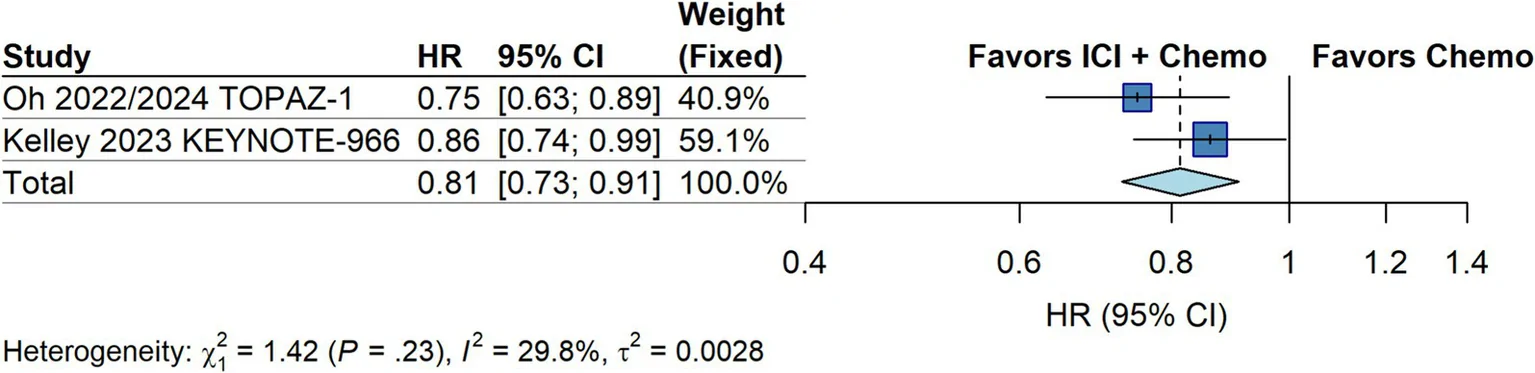
Forest plot of PFS for RCTs.

The above two phase III RCTs reported PFS data; the remaining 3 single-center RCTs did not report PFS and were not included in this pooling. Meta-analysis results showed that the combination group had significantly improved PFS (HR = 0.81, 95% CI: 0.71–0.93, *p* = 0.002), with heterogeneity *i*^2^ = 29.8%, *p* = 0.23, using a fixed-effects model (Figure 4). This model selection is consistent with the predefined protocol (*i*^2^ < 50% threshold).

#### 3.4.2 Retrospective studies

**Figure 5:**
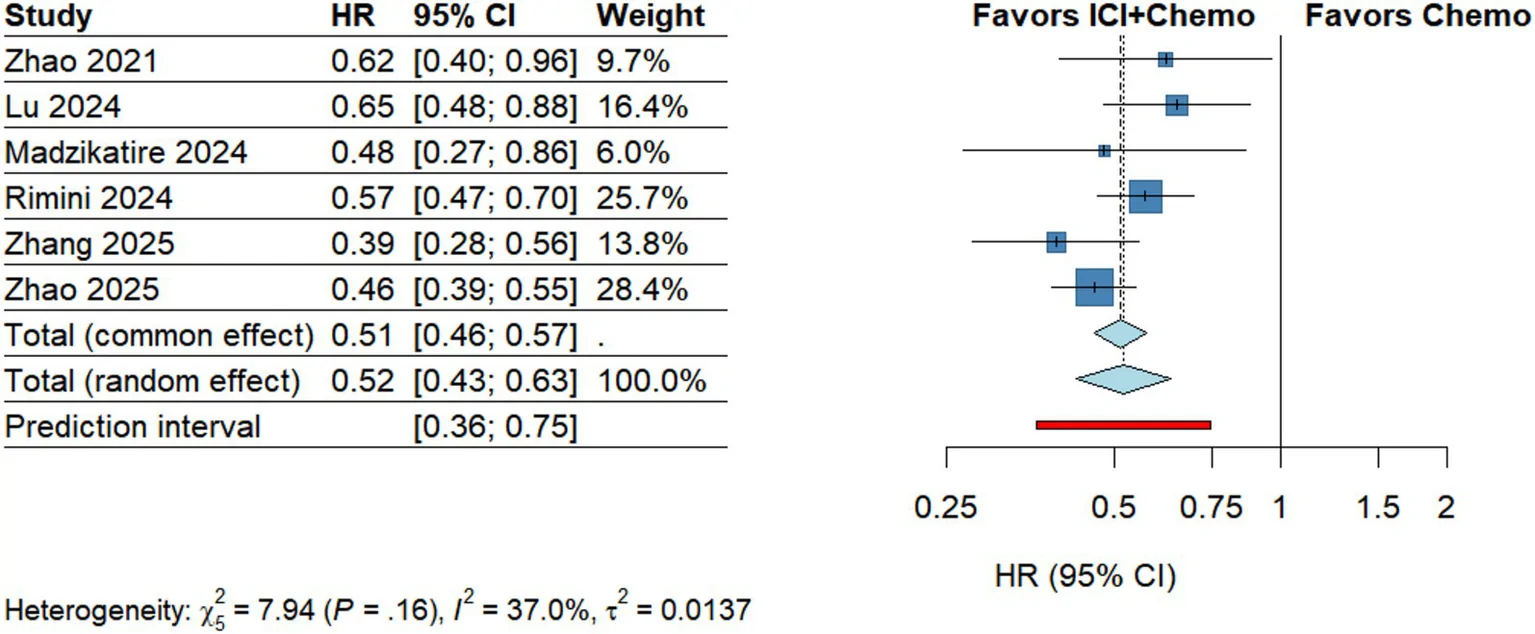
Forest plot of PFS for retrospective studies.

All 6 retrospective studies reported PFS data. Meta-analysis results showed that the combination group had significantly better PFS than the chemotherapy group (HR = 0.52, 95% CI: 0.43–0.63, *p* < 0.001), with moderate heterogeneity (*i*^2^ = 37%, *p* = 0.16) (Figure 5).

### 3.5 Objective response rate (ORR)

#### 3.5.1 Randomized controlled trials (RCTs)

**Figure 6:**
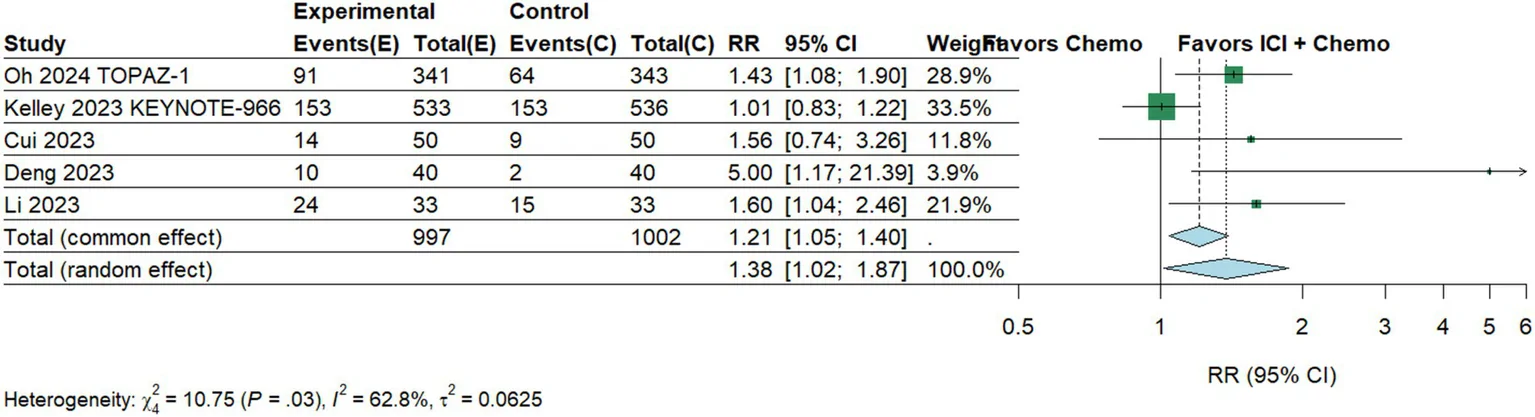
Forest plot of ORR for RCTs.

All 5 RCTs reported ORR data, encompassing a total of 1,999 patients. Meta-analysis results showed that the combination group had a significantly higher ORR than the chemotherapy-alone group (RR = 1.38, 95% CI: 1.02–1.87, *p* = 0.037), with heterogeneity *i*^2^ = 62.8%, p = 0.03 (Figure 6).

#### 3.5.2 Retrospective studies

**Figure 7:**
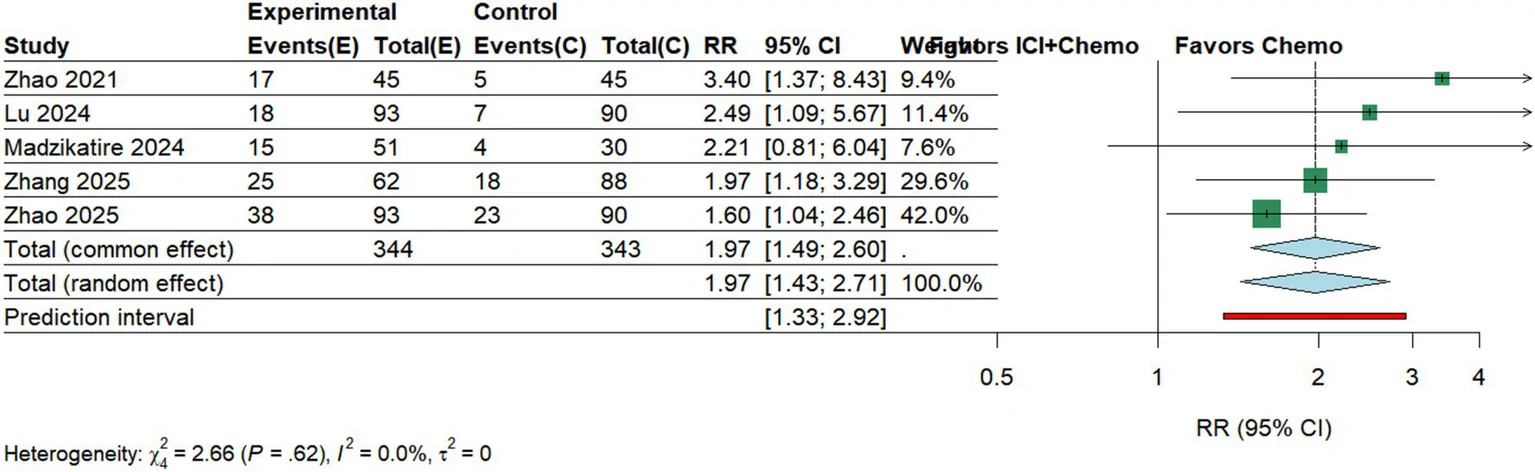
Forest plot of ORR for retrospective studies.

Five of the 6 retrospective studies reported ORR data. Meta-analysis results showed that the combination group had a significantly higher ORR than the chemotherapy group (RR = 1.97, 95% CI: 1.43–2.71, *p* = 0.004), with no heterogeneity (*i*^2^ = 0%) (Figure 7).

### 3.6 Disease control rate (DCR)

#### 3.6.1 Randomized controlled trials (RCTs)

**Figure 8:**
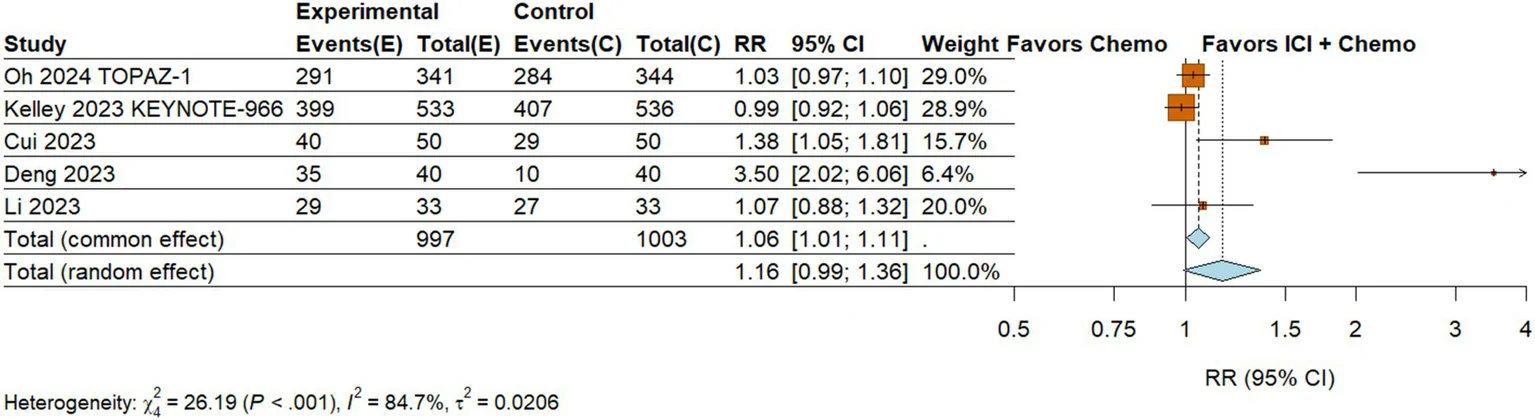
Forest plot of DCR for RCTs.

All 5 RCTs reported DCR data. There was no statistically significant difference in DCR between the two groups (RR = 1.16, 95% CI: 0.99–1.36, *p* = 0.059) (Figure 8).

#### 3.6.2 Retrospective studies

**Figure 9:**
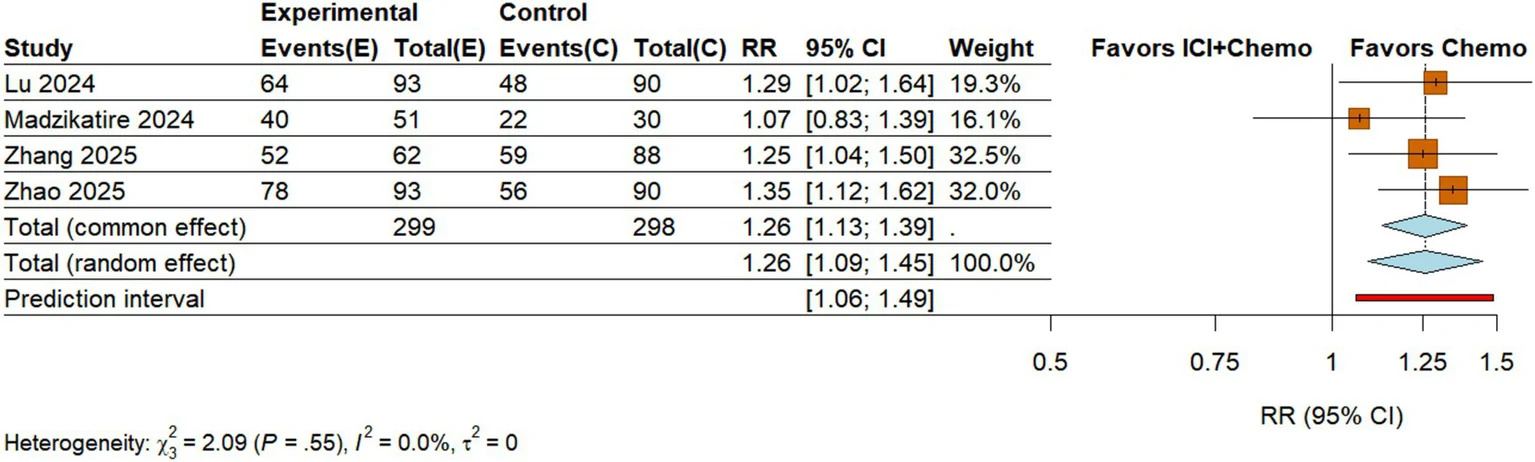
Forest plot of DCR for retrospective studies.

Four of the 6 retrospective studies reported DCR data. The combination group had a significantly higher DCR than the chemotherapy group (RR = 1.26, 95% CI: 1.09–1.45, *p* = 0.014) (Figure 9).

### 3.7 Safety analysis

#### 3.7.1 Randomized controlled trials (RCTs)

**Figure 10:**
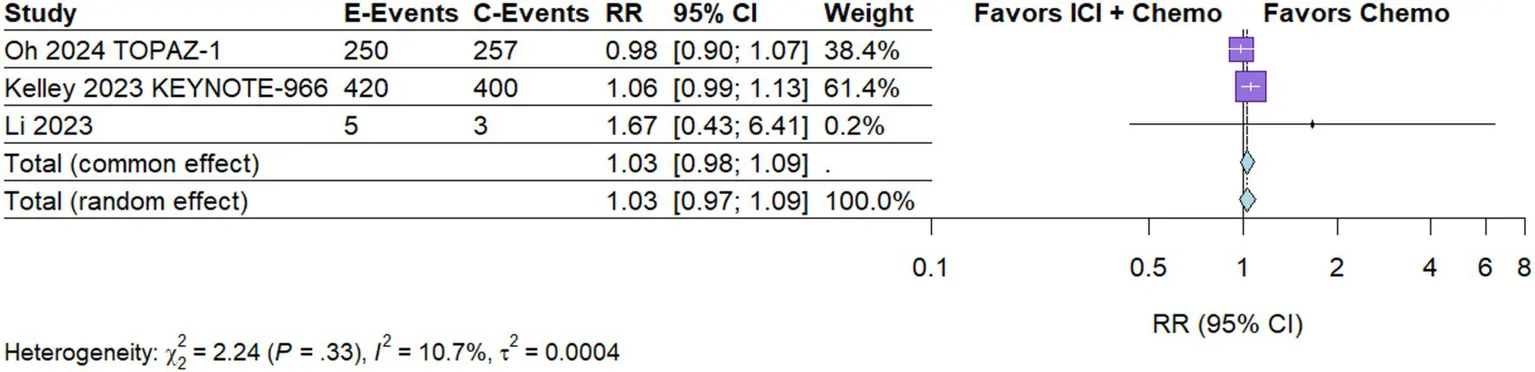
Forest plot of safety outcomes for RCTs.

Two phase III RCTs reported any-grade adverse events, showing no statistically significant difference between the two groups (RR = 1.00, 95% CI: 0.99–1.01, *p* = 0.847). Three studies reported grade 3–4 adverse drug reactions (ADRs), showing no statistically significant difference (RR = 1.03, 95% CI: 0.97–1.09, *p* = 0.311) (Figure 10).

#### 3.7.2 Retrospective studies

**Figure 11:**
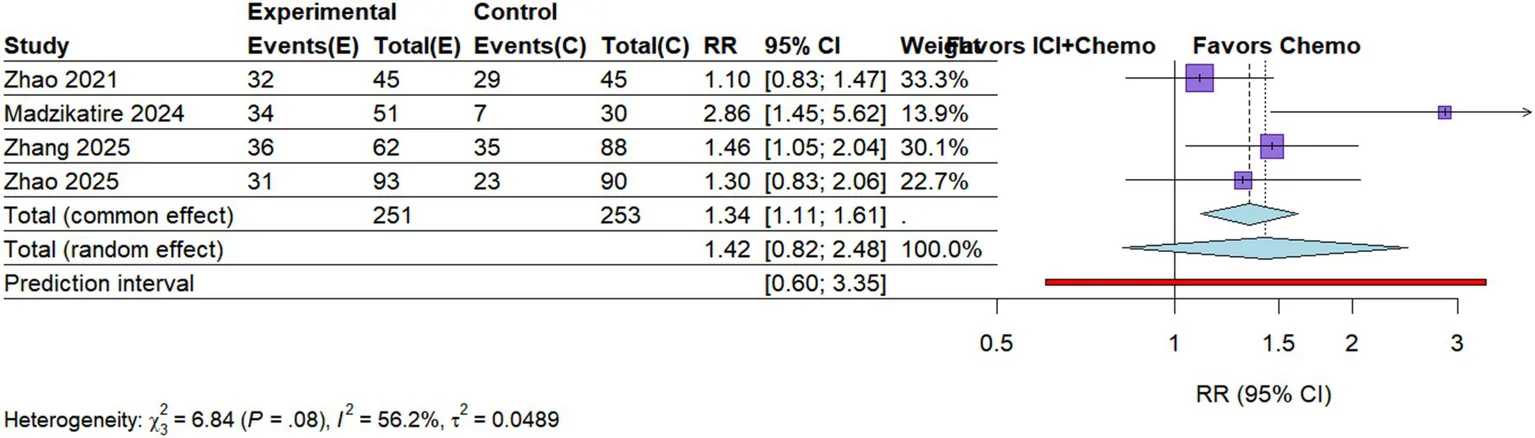
Forest plot of safety outcomes for retrospective studies.

Four of the 6 retrospective studies reported ≥grade 3 treatment-related adverse events (TRAEs). There was no statistically significant difference in the incidence of ≥grade 3 TRAEs between the two groups (RR = 1.42, 95% CI: 0.82–2.48, *p* = 0.136), with moderate heterogeneity (*i*^2^ = 56.2%) (Figure 11).

### 3.8 Sensitivity analysis and publication bias

#### 3.8.1 Randomized controlled trials (RCTs)

**Figure 12:**
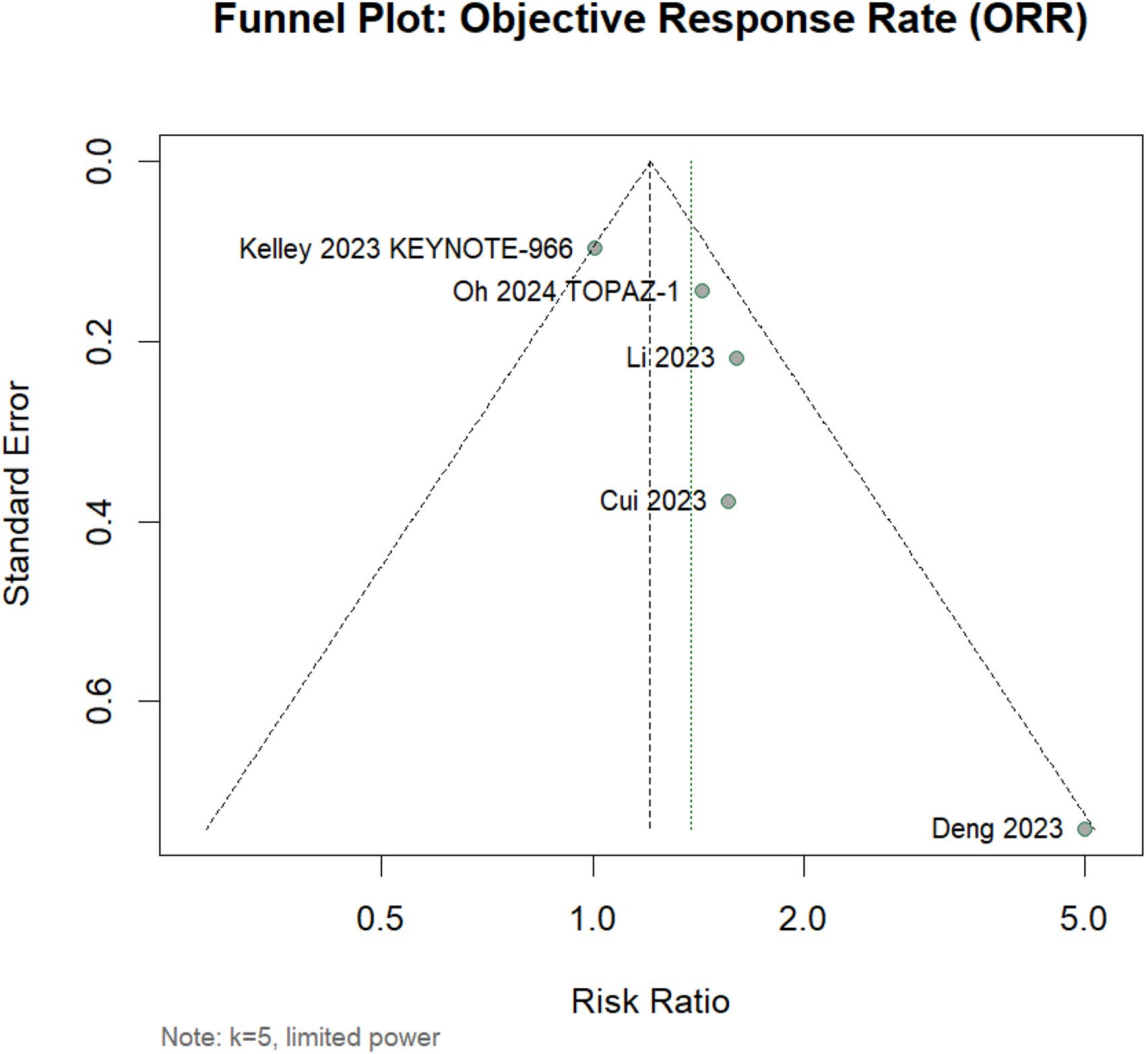
Sensitivity analysis and funnel plots for RCTs.

Both the RCT and retrospective study components employed the leave-one-out method for sensitivity analysis. For the ORR outcome, the pooled RR ranged from 1.29 to 1.53 after sequentially excluding each study. For the DCR outcome, heterogeneity was significantly reduced after excluding Deng 2023. Funnel plots did not reveal obvious publication bias (Figure 12).

#### 3.8.2 Retrospective studies

**Figure 13:**
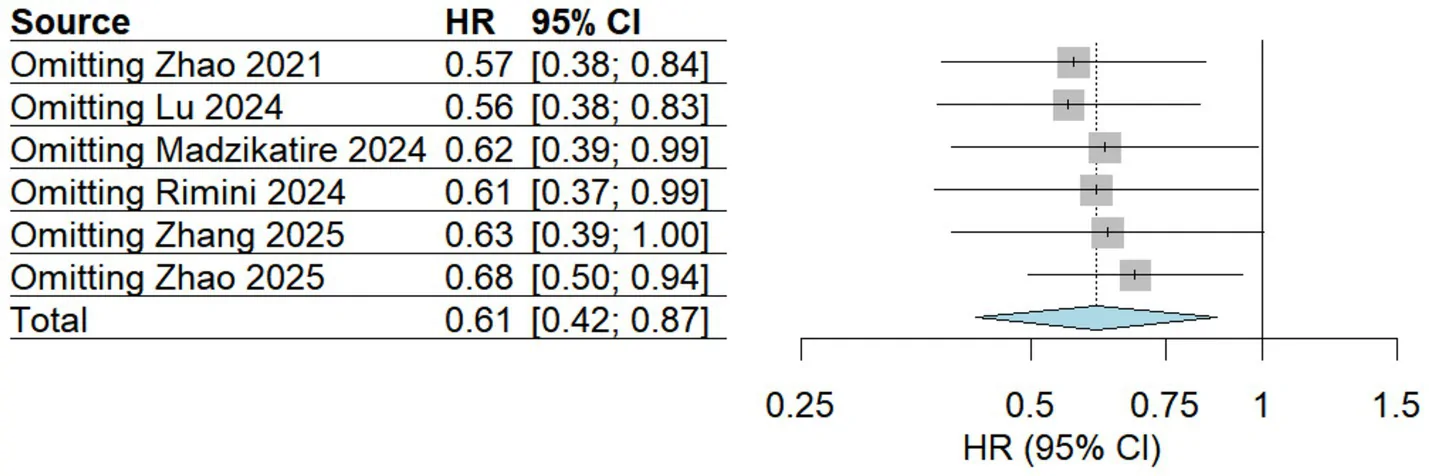
Sensitivity analysis for retrospective studies.

**Figure 14:**
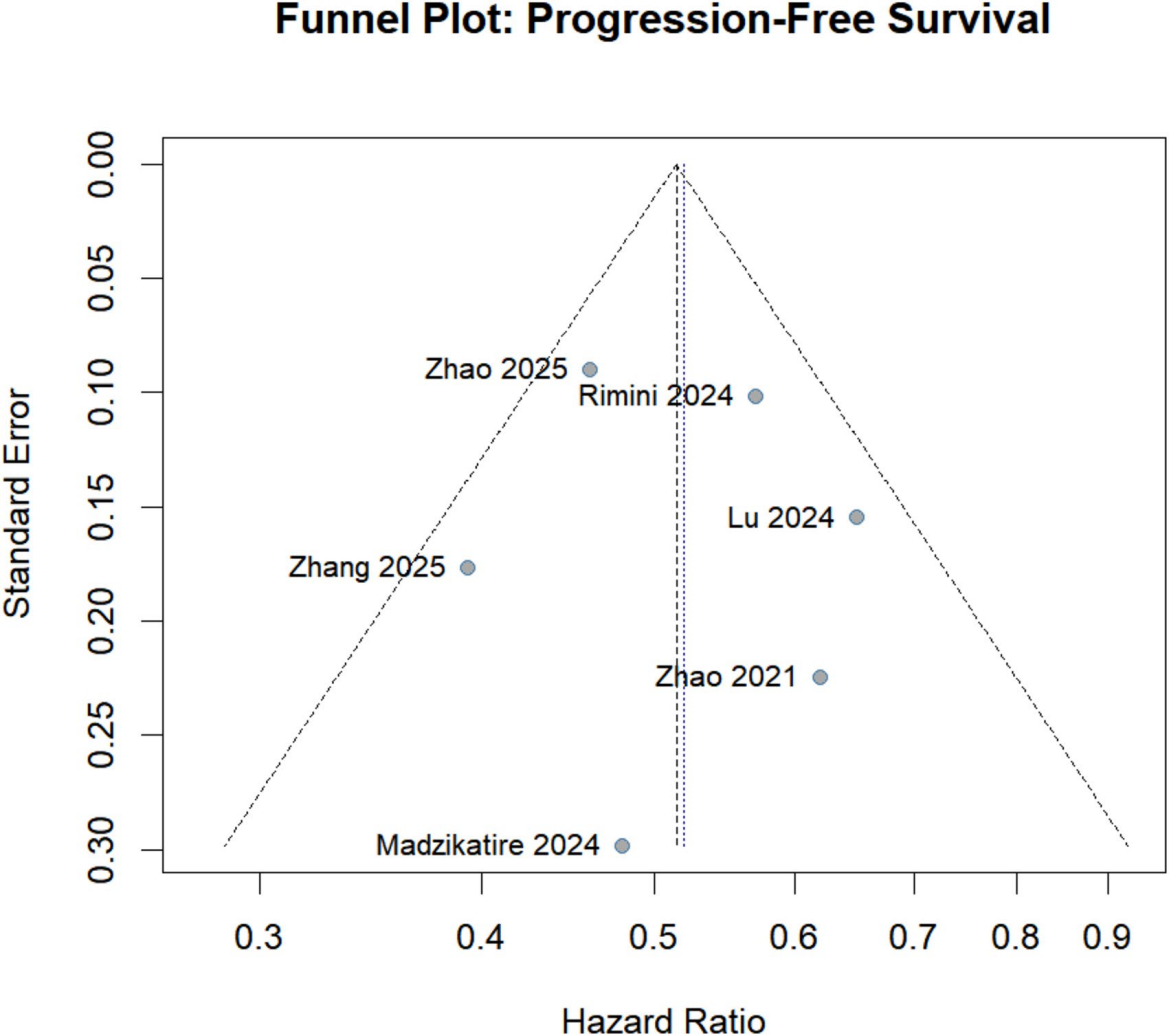
Funnel plots with trim-and-fill analysis for retrospective studies.

In the retrospective studies, after sequentially excluding each study, the pooled PFS HR fluctuated between 0.50 and 0.55, and the pooled OS HR fluctuated between 0.56 and 0.68. Trim-and-fill analysis indicated that the pooled effect sizes remained statistically significant (Figures 13 and 14). Sensitivity analyses excluding potentially overlapping studies (Zhao 2021 and/or Zhao 2025) showed robust pooled estimates: for PFS, the HR ranged from 0.51 to 0.55 across scenarios; for OS, the HR ranged from 0.57 to 0.67. Notably, excluding Zhao 2025 (which contributed the largest weight in the OS analysis) resulted in a modest attenuation of the OS effect (HR = 0.67, 95% CI: 0.50–0.90), but the benefit remained statistically significant (Supplementary Figure S1).

### 3.9 Subgroup analysis

**Figure 15:**
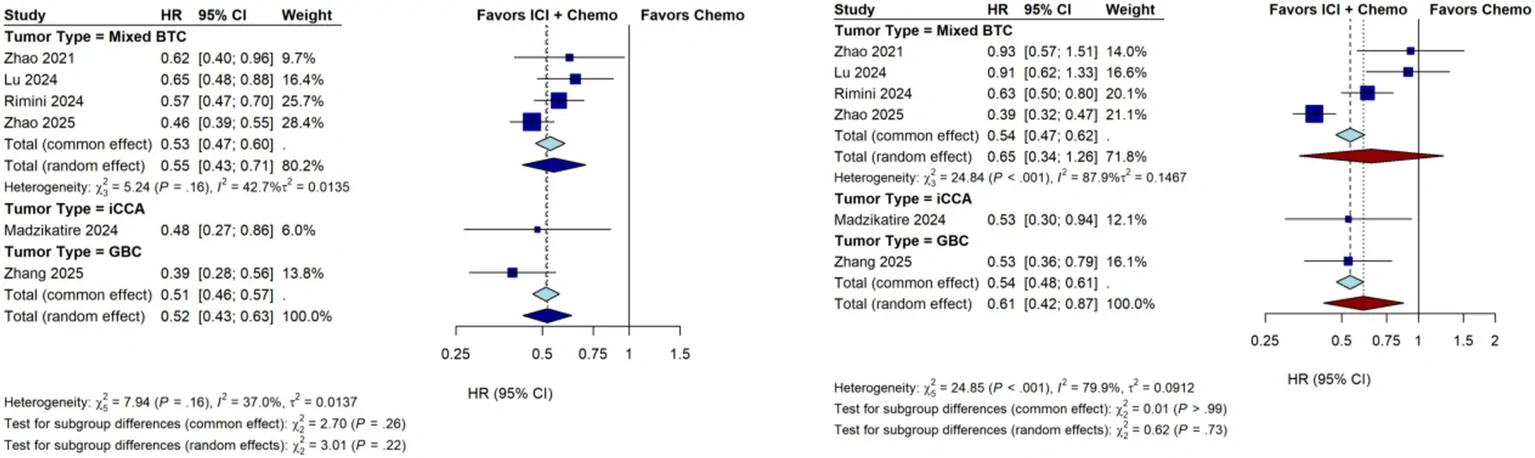
Subgroup analysis by tumor type (Subgroup differences were tested by Cochran’s *Q* test; results were consistent with meta-regression analysis).

Subgroup analysis by tumor type showed that for PFS, the HR was 0.55 (95% CI: 0.43–0.71) in the mixed BTC subgroup, 0.48 (95% CI: 0.27–0.86) in the iCCA subgroup, and 0.39 (95% CI: 0.28–0.56) in the GBC subgroup. For OS, the HR was 0.64 (95% CI: 0.34–1.19) in the mixed BTC subgroup, 0.53 (95% CI: 0.30–0.94) in the iCCA subgroup, and 0.53 (95% CI: 0.36–0.79) in the GBC subgroup (Figure 15). The interaction test for tumor type was not statistically significant for either PFS (*p* = 0.34) or OS (*p* = 0.88), indicating no significant differential effect across anatomical subtypes.

Subgroup analysis by ICI type showed that both PD-1 inhibitors (pooled HR 0.52, 95% CI: 0.43–0.63) and PD-L1 inhibitors (pooled HR 0.59, 95% CI: 0.50–0.70) were associated with improved PFS. For OS, PD-1 inhibitors showed a pooled HR of 0.53 (95% CI: 0.39–0.72), and PD-L1 inhibitors a pooled HR of 0.73 (95% CI: 0.57–0.95). The interaction test for the ICI type was not statistically significant for PFS (*p* = 0.11) or OS (*p* = 0.18), suggesting no conclusive evidence of differential efficacy between PD-1 and PD-L1 inhibitors in this dataset (Supplementary Figures S2a,b). However, given the limited number of studies per subgroup and the predominance of single-arm observations, these comparisons should be interpreted with caution.

## 4 Discussion

This study integrated two types of research evidence—RCTs and retrospective studies—to systematically evaluate the efficacy and safety of PD-1/PD-L1 inhibitors combined with chemotherapy as first-line treatment for advanced BTC. Part I included 5 RCTs with 2,000 patients; the survival benefit (OS HR = 0.80; PFS HR = 0.81) was demonstrated by the 2 phase III trials (*n* = 1,754), while the ORR benefit (RR = 1.38) was pooled from all 5 RCTs.

Regarding the three single-center small-sample RCTs (Cui 2023, Deng 2023, Li 2023), all fully met the PICOS (Population, Intervention, Comparator, Outcome, Study design) inclusion criteria: they enrolled patients with histopathologically confirmed advanced BTC (Population), evaluated PD-1/PD-L1 inhibitors plus chemotherapy versus chemotherapy alone (Intervention/Comparator), reported at least one efficacy outcome (ORR and/or DCR) (Outcome), and employed a randomized controlled design (Study design). Their inclusion serves two purposes: first, to augment the sample size for short-term efficacy endpoints (ORR and DCR), thereby increasing statistical power for these outcomes; second, to explore efficacy signals of additional PD-1/PD-L1 inhibitors (nivolumab, sintilimab) beyond the two phase III agents, providing preliminary evidence for broader class-effect hypotheses. We acknowledge that these single-center studies have limitations, including small sample sizes and unclear risk of bias in some domains, which is why survival outcomes (OS and PFS) are pooled exclusively from the two phase III trials.

ICI combined with chemotherapy significantly improved OS (HR = 0.80), PFS (HR = 0.81), and ORR (RR = 1.38) compared with chemotherapy alone, without increasing the incidence of any-grade or grade 3–4 ADRs. Part II included 6 retrospective studies with 1,253 patients, showing that the combination group significantly prolonged PFS (HR = 0.52) and OS (HR = 0.61), while significantly improving ORR (RR = 1.97) and DCR (RR = 1.26), without increasing the risk of ≥grade 3 TRAEs. The results of both parts were highly consistent, jointly supporting PD-1/PD-L1 inhibitors combined with chemotherapy as an effective first-line treatment option for advanced BTC.

Regarding OS, the pooled HR was 0.80 in the RCT component and 0.61 in the retrospective study component, both indicating that ICI combined with chemotherapy can provide significant survival benefits. Notably, the results of two phase III studies (TOPAZ-1: HR = 0.76; KEYNOTE-966: HR = 0.83) were directionally consistent, with zero heterogeneity, enhancing the reliability of the conclusions. It is important to note that the study by Rimini et al. (21) is a retrospective real-world study and is included exclusively in the retrospective study component; it is not a RCT and was not included in the RCT meta-analysis. TOPAZ-1 (4, 5) and KEYNOTE-966 are the only phase III RCTs in the RCT component. The latest updated data from the TOPAZ-1 study showed that the 24-month survival rate improved from 18% in the placebo group to 25% in the experimental group (22). TOPAZ-1 subgroup analysis further showed greater OS benefit in recurrent vs. initially unresectable disease (HR 0.56 vs. 0.84) (23). A real-world multicenter study (*n* = 563) confirmed durvalumab plus gemcitabine/cisplatin improved OS versus chemotherapy alone (median OS: 14.6 vs. 11.2 months, HR = 0.72, *p* = 0.002) (21). A larger global real-world study (TriNetX, *n* = 1,099) further confirmed the OS benefit of durvalumab plus chemotherapy versus chemotherapy alone (post-PSM median OS: 418 vs. 359 days; HR = 0.86, 95% CI: 0.76–0.98, *p* = 0.022) (24) in the KEYNOTE-966 study reached 12.7 months, an extension of 1.8 months compared with the control group (25). In the prespecified China subpopulation of KEYNOTE-966 (*n* = 158), pembrolizumab plus chemotherapy showed numeric OS improvement versus placebo (median OS not reached vs. 13.5 months; HR = 0.80, 95% CI: 0.60–1.07), with 49% of responders maintaining response ≥12 months and no new safety signals (26).

Our meta-analysis observed a numerically larger effect size in retrospective studies (OS HR = 0.61; PFS HR = 0.52) compared with RCTs (OS HR = 0.80; PFS HR = 0.81). This discrepancy is a common phenomenon in oncology meta-analyses and may reflect differences in study design rather than a true divergence in treatment efficacy. Retrospective studies typically employ less stringent selection criteria and lack standardized follow-up protocols, which may result in the inclusion of patients with better performance status or less aggressive disease—factors that can inflate the observed treatment effect. In contrast, RCTs maintain rigorous eligibility criteria and centralized outcome adjudication, yielding a more conservative but unbiased estimate of efficacy. Nevertheless, RCTs remain the gold standard for evidence-based medicine, and our pooled results from the two phase III trials provide the most robust estimate of the true treatment effect. We did not perform a direct statistical comparison between RCT and retrospective study results, as they address fundamentally different research questions (efficacy versus effectiveness). The consistent direction of benefit across both study types—despite the quantitative difference in effect size—strengthens the overall confidence in the clinical value of PD-1/PD-L1 inhibitor combination therapy. Importantly, RCTs remain the gold standard of evidence-based medicine and carry the highest weight in our conclusion. The primary efficacy estimates (OS HR = 0.80, PFS HR = 0.81) are derived exclusively from the two phase III RCTs, which provide the most rigorous and unbiased assessment of treatment efficacy. Retrospective real-world data serve as complementary evidence, supporting the generalizability of findings to broader clinical practice but should not be interpreted as confirmatory.

Regarding PFS, the pooled HR was 0.81 in the RCT component, consistent with the OS benefit trend. However, the first interim analysis of PFS in the KEYNOTE-966 study did not reach the prespecified statistical significance boundary. The investigators noted that PFS assessment in BTC is affected by multiple non-radiological factors, and PFS may not be the optimal efficacy endpoint for BTC (6). The pooled PFS HR in the retrospective study component was 0.52, with a more pronounced effect size compared with the RCT component, which may be related to the greater heterogeneity of the enrolled population in retrospective studies, making them more reflective of real-world clinical practice.

Regarding ORR, the pooled RR was 1.38 in the RCT component and 1.97 in the retrospective study component, both indicating that ICI combined with chemotherapy can significantly improve the objective response rate. Subgroup analysis showed that patients with iCCA (HR = 0.48) and GBC (HR = 0.39) demonstrated trends toward greater benefit from combination therapy compared with the mixed BTC population. Although the interaction test did not reach statistical significance, this trend suggests that PD-1/PD-L1 inhibitors combined with chemotherapy may have efficacy across different anatomical subtypes of BTC. However, we acknowledge that the evidence for GBC specifically is limited: the two phase III RCTs showed that the GBC subgroup did not achieve statistical significance for OS in either TOPAZ-1 (HR 0.90, 95% CI: 0.65–1.24) or KEYNOTE-966 (HR 0.96, 95% CI: 0.73–1.26). Our meta-analysis of retrospective studies suggests a potential benefit in GBC (PFS HR = 0.39; OS HR = 0.53), but these findings are derived from single-center observational studies with small sample sizes and should be interpreted cautiously. The apparent benefit in GBC may reflect selection bias or differences in baseline prognostic factors rather than a true differential treatment effect. Supporting this notion, a phase II single-arm study of toripalimab (a PD-1 inhibitor) combined with gemcitabine and S-1 in 50 Chinese patients with advanced BTC reported a median PFS of 7.0 months, median OS of 15.0 months, and ORR of 30.6%, with manageable toxicity (27). These results, though from a non-randomized design, align with the efficacy signals observed in our meta-analysis and warrant further validation in larger controlled studies. Furthermore, a real-world PSM study in GBC showed that adding PD-1 inhibitors to chemotherapy significantly improved ORR (31.3% vs. 6.3% after PSM, *p* = 0.015), with 28.3% of patients becoming eligible for surgical conversion, supporting our meta-analysis finding that GBC patients may derive particular benefit from chemoimmunotherapy (28). Similarly, a GBC-focused retrospective study (23, 29) confirmed that adding PD-1 inhibitors to chemotherapy significantly improved OS (18.2 vs. 6.1 months, *p* = 0.016), PFS (11.3 vs. 5.5 months, *p* = 0.008), and ORR (35.5% vs. 5.3%, *p* = 0.037) (GBC 94P study, 2023), further supporting our meta-analysis finding that GBC patients derive substantial benefit from chemoimmunotherapy. Supporting this, a phase 2 study of durvalumab ± tremelimumab plus gemcitabine/cisplatin reported ORR of 66% (72% with durvalumab alone) in 124 advanced BTC patients (24, 30), suggesting potential for enhanced response with dual immunotherapy. In summary, the evidence for GBC benefit is stratified by study design: phase III RCTs show no statistically significant OS benefit in the GBC subgroup (TOPAZ-1 HR 0.90, KEYNOTE-966 HR 0.96), while retrospective studies suggest a more pronounced effect (pooled OS HR 0.53). This discrepancy likely reflects selection bias and smaller sample sizes in retrospective cohorts, and the GBC subgroup finding should be considered hypothesis-generating pending further prospective validation.

We recognize that the RCT and retrospective study components yielded numerically different effect sizes (RCT PFS HR = 0.81 vs. retrospective PFS HR = 0.52; RCT OS HR = 0.80 vs. retrospective OS HR = 0.61). We intentionally did not pool these two components, as RCTs provide higher-quality evidence with lower risk of confounding bias. The retrospective data serve as complementary real-world evidence rather than confirmatory evidence. The consistency of direction (both favoring immunotherapy combination) across study designs strengthens the overall conclusion, but the magnitude of benefit should be primarily guided by the RCT estimates.

Regarding ICI heterogeneity, the included studies evaluated multiple PD-1 inhibitors (nivolumab, pembrolizumab, sintilimab, camrelizumab (31), tislelizumab, toripalimab) and PD-L1 inhibitors (durvalumab). Subgroup analysis by ICI type did not reveal statistically significant interaction (PFS: *p* = 0.11; OS: *p* = 0.18). However, we acknowledge that all approved agents for this indication (durvalumab and pembrolizumab) are supported by phase III RCT evidence, while the Chinese single-center RCTs used non-approved agents (nivolumab, sintilimab) and should be considered exploratory. We do not claim equivalence across all PD-1/PD-L1 inhibitors; the conclusion is restricted to the class effect observed in the available evidence.

This study used the R language for meta-analysis, which offers greater flexibility and extensibility compared with the commonly used RevMan software. Through the meta and metafor packages, not only standard retrieval, data extraction, and statistical analysis can be performed, but also more complex subgroup analyses, meta-regression, and network meta-analysis can be conducted.

The limitations of this study include: (1) only 2 phase III RCTs reported OS and PFS data in part I, while the remaining 3 small-sample RCTs did not report survival data; (2) inconsistencies in chemotherapy regimens and ICI types among included studies resulted in clinical heterogeneity; (3) retrospective studies are subject to inherent confounding bias; (4) five of the six retrospective studies were from China, resulting in a geographically imbalanced distribution; (5) the lack of individual patient data prevented more precise subgroup analysis. (6) Zhao 2021 and Zhao 2025 were conducted at the same center, raising the possibility of patient cohort overlap; sensitivity analyses showed robustness of the overall conclusions, but the OS estimate was modestly attenuated when excluding Zhao 2025 (HR = 0.67 vs. 0.61 for the full dataset).

## 5 Conclusion

The results of this meta-analysis demonstrate that, compared with chemotherapy alone, PD-1/PD-L1 inhibitors combined with chemotherapy as first-line treatment for advanced BTC can significantly improve OS, PFS, and ORR without increasing the incidence of severe adverse events. The RCT component provides high-quality evidence, with survival data derived from two large phase III trials for this treatment strategy, while the retrospective study component further validates its clinical value from a real-world perspective. The results of both parts are highly consistent, jointly supporting PD-1/PD-L1 inhibitors combined with chemotherapy as an effective first-line treatment option for advanced BTC.

Future research requires more high-quality RCTs to further validate the optimal combination of different PD-1/PD-L1 inhibitors and chemotherapy regimens, and to explore predictive biomarkers for efficacy, including peripheral inflammatory markers (32), tumor tissue IL-21/IL-33 expression profiles associated with treatment response (33), hepatitis B virus infection status, which has been shown to influence the efficacy of pembrolizumab plus chemotherapy (34), as well as established biomarkers such as PD-L1 expression, microsatellite instability status, and tumor mutational burden, in order to achieve precision immunotherapy for advanced BTC.

## Data Availability

The original contributions presented in the study are included in the article/Supplementary material, further inquiries can be directed to the corresponding author.
