# Supplementary material for: PD-1/PD-L1 inhibitors combined with chemotherapy as first-line treatment for advanced biliary tract cancer: a systematic review and meta-analysis
Source: Front Med (Lausanne). 2026 Sep 3;13:1916331. doi: 10.3389/fmed.2026.1916331 (PMC13581943; doi:10.3389/fmed.2026.1916331)
Supplement: Supplementary file 1 [file Data_Sheet_4.PDF]

Supplementary Code

PD-1/PD-L1 Inhibitors Combined with Chemotherapy
as First-Line Treatment for Advanced Biliary Tract Cancer:
A Systematic Review and Meta-Analysis

Journal: Frontiers in Medicine

This supplementary material contains R code for reproducing all meta-analyses and figures in the manuscript. All study data are embedded directly in the scripts for full reproducibility.

Supplementary Code - README

Manuscript

PD-1/PD-L1 Inhibitors Combined with Chemotherapy as First-Line Treatment for Advanced Biliary Tract Cancer: A Systematic Review and Meta-Analysis

Journal: Frontiers in Medicine

Contents

This supplementary material contains R code for reproducing all meta-analyses and figures in the manuscript. All study data are embedded directly in the scripts for full reproducibility.

File 1: Supplementary_Code_1_RCT_Meta_Analysis.R

Purpose: Meta-analysis of 5 randomized controlled trials (RCTs)

Analyses included:

- Overall Survival (OS) - 2 phase III RCTs (TOPAZ-1, KEYNOTE-966)
- Progression-Free Survival (PFS) - 2 phase III RCTs
- Objective Response Rate (ORR) - all 5 RCTs
- Disease Control Rate (DCR) - all 5 RCTs
- Safety outcomes (any-grade and grade 3-4 AEs)
- Sensitivity analysis (leave-one-out)
- Publication bias (funnel plots)

Corresponds to: Part I (RCT component) of the manuscript, Figures 2-12

File 2: Supplementary_Code_2_Retrospective_Meta_Analysis.R

Purpose: Meta-analysis of 6 retrospective studies

Analyses included:

- Progression-Free Survival (PFS) - all 6 studies
- Overall Survival (OS) - all 6 studies
- Objective Response Rate (ORR) - 5 studies
- Disease Control Rate (DCR) - 4 studies
- Safety outcomes (grade >=3 TRAEs)
- Sensitivity analysis (leave-one-out)

- Publication bias (funnel plots with trim-and-fill)
- Subgroup analyses (tumor type, geographic region, study design)

Corresponds to: Part II (retrospective component) of the manuscript, Figures 3, 5-11, 13-14

File 3: Supplementary_Code_3_Revised_Figures_and_Supplementary_Plots.R

Purpose: Figures revised in response to reviewer comments and newly added supplementary plots

Figures generated:

- **Figure 4 (revised):** RCT PFS forest plot using fixed-effect model (corrected per Reviewer 1 comment)
- **Figure 15a:** Retrospective PFS subgroup forest plot by tumor type
- **Figure 15b:** Retrospective OS subgroup forest plot by tumor type (added per Reviewer 1 comment)
- **Supplementary Figure S1:** Cohort overlap sensitivity analysis (4 scenarios: exclude Zhao 2021, exclude Zhao 2025, exclude both, full dataset) - added per Reviewer 1 comment
- **Supplementary Figure S2a/S2b:** Retrospective subgroup forest plots by ICI type (PD-1 vs PD-L1) for PFS and OS - added per Reviewer 3 comment

Corresponds to: Revised Figure 4, Figure 15, Supplementary Figures S1-S2

How to Run

Prerequisites

- R version 4.6.1 or later
- Required packages will be installed automatically if missing:

  - `meta` (v8.5-0)
  - `metafor` (v5.0-1)
  - `ggplot2`
  - `gridExtra`
  - `grid`

Instructions

1. Open R or RStudio
2. Set working directory to the folder containing the scripts
3. Source each script from top to bottom:

```r
source("Supplementary_Code_1_RCT_Meta_Analysis.R")
source("Supplementary_Code_2_Retrospective_Meta_Analysis.R")
```

source("Supplementary_Code_3_Revised_Figures_and_Supplementary_Plots.R")

```

4. Output figures will be saved to subdirectories (e.g., `meta_analysis_output/`, `reviewer_revised_figures/`)

### Output Format

- All figures are saved in both **PNG (300 dpi)** and **PDF** formats
- Forest plots include study names, effect sizes, 95% confidence intervals, heterogeneity statistics, and pooled estimates

## Reproducibility Notes

- All study-level data are extracted from the published literature and embedded directly in the scripts
- Random seed is set for reproducibility where applicable
- The scripts include both fixed-effect and random-effects model specifications as appropriate
- Heterogeneity is assessed using the Q test and $I^2$ statistic
- Sensitivity analyses use the leave-one-out method

## Contact

For questions regarding the code, please contact the corresponding author.
```

Supplementary Code 1: RCT Meta-Analysis

File: Supplementary_Code_1_RCT_Meta_Analysis.R

Purpose: Meta-analysis of 5 randomized controlled trials (RCTs)

Corresponds to: Part I (RCT component) of the manuscript, Figures 2-12

```
# ==============================================================================
# Supplementary Code for:
#   PD-1/PD-L1 Inhibitors Combined with Chemotherapy as First-Line
#   Treatment for Advanced Biliary Tract Cancer:
#   A Systematic Review and Meta-Analysis
#
# Journal: Frontiers in Medicine
#
# File: Supplementary_Code_1_RCT_Meta_Analysis.R
#
# Description:
#   Meta-analysis of 5 randomized controlled trials (RCTs) evaluating PD-1/PD-L1
#
# Corresponds to: Part I (RCT component): OS, PFS, ORR, DCR, and safety outcomes
#
# R version: 4.6.1
# Required packages:
#   - meta     (v8.5-0)
#   - metafor  (v5.0-1)
#   - ggplot2
#   - gridExtra
#   - grid
#
# Notes:
#   - All study data are embedded directly in the script for full reproducibility
#   - Figures are saved as both PNG (300 dpi) and PDF formats
#   - Run the script from top to bottom to reproduce all analyses
#
# ==============================================================================

# Step 1: Install and load required packages
# ------------------------------------------------------------------------------

packages <- c("meta", "metafor", "ggplot2", "gridExtra", "grid")

install_if_missing <- function(pkg) {
  if (!requireNamespace(pkg, quietly = TRUE)) {
    install.packages(pkg, repos = "https://cran.r-project.org")
  }
}

invisible(sapply(packages, install_if_missing))

library(meta)
```

```
library(metafor)
library(ggplot2)
library(gridExtra)
library(grid)

# Set output directory
output_dir <- "./meta_analysis_output"
dir.create(output_dir, showWarnings = FALSE, recursive = TRUE)

# ============================================================================
# Step 2: Helper functions
# ============================================================================

calc_se_loghr <- function(lower, upper) {
  (log(upper) - log(lower)) / (2 * 1.96)
}

get_pooled <- function(m, use_fixed = FALSE) {
  if (use_fixed || m$I2 == 0) {
    list(TE = m$TE.fixed, lower = m$lower.fixed, upper = m$upper.fixed,
         pval = m$pval.fixed, zval = m$zval.fixed, model = "Fixed")
  } else {
    list(TE = m$TE.random, lower = m$lower.random, upper = m$upper.random,
         pval = m$pval.random, zval = m$zval.random, model = "Random (DL)")
  }
}

# ============================================================================
# Step 3: Study characteristics
# ============================================================================

study_chars <- data.frame(
  Study = c("Oh 2024 TOPAZ-1", "Kelley 2023 KEYNOTE-966",
            "Cui 2023", "Deng 2023", "Li 2023"),
  Year = c(2024, 2023, 2023, 2023, 2023),
  Phase = c("III", "III", "Single-center", "Single-center", "Single-center"),
  Country = c("17 countries", "Global", "China", "China", "China"),
  N_Exp = c(341, 533, 50, 40, 33),
  N_Ctrl = c(344, 536, 50, 40, 33),
  N_Total = c(685, 1069, 100, 80, 66),
  ICI = c("Durvalumab (PD-L1)", "Pembrolizumab (PD-1)",
          "Nivolumab (PD-1)", "Pembrolizumab (PD-1)", "Sintilimab (PD-1)"),
  Chemo = c("GemCis", "GemCis", "GEMOX", "XELOX", "GC")
)

cat("\n========================================\n")
cat("  STUDY CHARACTERISTICS\n")
cat("========================================\n\n")
print(study_chars, row.names = FALSE)
write.csv(study_chars, file.path(output_dir, "study_characteristics.csv"),
          row.names = FALSE, fileEncoding = "UTF-8")
cat("  Saved: study_characteristics.csv\n")

# ============================================================================
# Step 4: Embed all study outcome data
```

```
# ============================================================================

# ---- 4.1 OS data ----
os_data <- data.frame(
  study = c("Oh 2024 TOPAZ-1", "Kelley 2023 KEYNOTE-966"),
  hr = c(0.76, 0.83), lower = c(0.64, 0.72), upper = c(0.91, 0.95),
  loghr = c(-0.2744, -0.1863), se = c(0.0898, 0.0707),
  n_exp = c(341, 533), n_ctrl = c(344, 536)
)

# ---- 4.2 PFS data ----
pfs_data <- data.frame(
  study = c("Oh 2022 TOPAZ-1", "Kelley 2023 KEYNOTE-966"),
  hr = c(0.75, 0.86), lower = c(0.63, 0.75), upper = c(0.89, 1.00),
  loghr = c(-0.2877, -0.1508), se = c(0.0881, 0.0734),
  n_exp = c(341, 533), n_ctrl = c(344, 536)
)

# ---- 4.3 ORR data ----
orr_data <- data.frame(
  study = c("Oh 2024 TOPAZ-1", "Kelley 2023 KEYNOTE-966",
            "Cui 2023", "Deng 2023", "Li 2023"),
  responders_exp = c(91, 153, 14, 10, 24),
  n_exp = c(341, 533, 50, 40, 33),
  responders_ctrl = c(64, 153, 9, 2, 15),
  n_ctrl = c(343, 536, 50, 40, 33),
  phase = c("Phase III", "Phase III", "Single-center",
            "Single-center", "Single-center"),
  chemo_type = c("GemCis", "GemCis", "Non-GemCis", "Non-GemCis", "Non-GemCis")
)

# ---- 4.4 DCR data ----
dcr_data <- data.frame(
  study = c("Oh 2024 TOPAZ-1", "Kelley 2023 KEYNOTE-966",
            "Cui 2023", "Deng 2023", "Li 2023"),
  dcr_exp = c(291, 399, 40, 35, 29),
  n_exp = c(341, 533, 50, 40, 33),
  dcr_ctrl = c(284, 407, 29, 10, 27),
  n_ctrl = c(344, 536, 50, 40, 33),
  phase = c("Phase III", "Phase III", "Single-center",
            "Single-center", "Single-center"),
  chemo_type = c("GemCis", "GemCis", "Non-GemCis", "Non-GemCis", "Non-GemCis")
)

# ---- 4.5 AE Any Grade ----
ae_any_data <- data.frame(
  study = c("Oh 2024 TOPAZ-1", "Kelley 2023 KEYNOTE-966"),
  events_exp = c(336, 524), n_exp = c(338, 529),
  events_ctrl = c(338, 532), n_ctrl = c(342, 534)
)

# ---- 4.6 Grade 3-4 AE ----
g34_ae_data <- data.frame(
  study = c("Oh 2024 TOPAZ-1", "Kelley 2023 KEYNOTE-966", "Li 2023"),
  events_exp = c(250, 420, 5), n_exp = c(338, 529, 33),
```

```
  events_ctrl = c(257, 400, 3), n_ctrl = c(342, 534, 33)
)

# ============================================================================
# STEP 5: META-ANALYSIS FOR OS
# ============================================================================

cat("\n========================================\n")
cat("  META-ANALYSIS: OVERALL SURVIVAL (OS)\n")
cat("========================================\n\n")

meta_os <- metagen(
  TE = os_data$loghr, seTE = os_data$se, studlab = os_data$study,
  sm = "HR", method.tau = "DL", hakn = FALSE,
  title = "Overall Survival (OS)",
  comb.fixed = TRUE, comb.random = FALSE
)
print(summary(meta_os))

# OS Forest Plot (k=2, compact layout)
png(file.path(output_dir, "forest_OS.png"), width = 1200, height = 500, res = 150)
forest(meta_os,
       layout = "JAMA", fontsize = 10,
       leftcols = c("studlab", "effect", "ci", "w.fixed"),
       leftlabs = c("Study", "HR", "95% CI", "Weight"),
       rightcols = c("effect", "ci"),
       rightlabs = c("HR", "95% CI"),
       col.diamond = "darkred", col.square = "indianred",
       col.square.lines = "indianred4", col.inside = "white",
       print.I2 = TRUE, print.tau2 = TRUE, print.pval.Q = TRUE,
       prediction = FALSE,
       xlim = c(0.1, 2.0), at = c(0.25, 0.5, 0.75, 1.0, 1.5, 2.0),
       ref = 1,
       label.left = "Favors ICI + Chemo",
       label.right = "Favors Chemo",
       main = "OS: ICI + Chemo vs. Chemo Alone in Advanced BTC"
)
dev.off()
cat("  Saved: forest_OS.png\n")

# ============================================================================
# STEP 6: META-ANALYSIS FOR PFS
# ============================================================================

cat("\n========================================\n")
cat("  META-ANALYSIS: PROGRESSION-FREE SURVIVAL (PFS)\n")
cat("========================================\n\n")

meta_pfs <- metagen(
  TE = pfs_data$loghr, seTE = pfs_data$se, studlab = pfs_data$study,
  sm = "HR", method.tau = "DL", hakn = FALSE,
  title = "Progression-Free Survival (PFS)",
  comb.fixed = TRUE, comb.random = TRUE
)
print(summary(meta_pfs))
```

```
# PFS Forest Plot (k=2)
png(file.path(output_dir, "forest_PFS.png"), width = 1200, height = 500, res = 150)
forest(meta_pfs,
       layout = "JAMA", fontsize = 10,
       leftcols = c("studlab", "effect", "ci", "w.random"),
       leftlabs = c("Study", "HR", "95% CI", "Weight"),
       rightcols = c("effect", "ci"),
       rightlabs = c("HR", "95% CI"),
       col.diamond = "darkblue", col.square = "steelblue",
       col.square.lines = "steelblue4", col.inside = "white",
       print.I2 = TRUE, print.tau2 = TRUE, print.pval.Q = TRUE,
       prediction = FALSE,
       xlim = c(0.1, 2.0), at = c(0.25, 0.5, 0.75, 1.0, 1.5, 2.0),
       ref = 1,
       label.left = "Favors ICI + Chemo",
       label.right = "Favors Chemo",
       main = "PFS: ICI + Chemo vs. Chemo Alone in Advanced BTC"
)
dev.off()
cat("  Saved: forest_PFS.png\n")

# ============================================================================
# STEP 7: META-ANALYSIS FOR ORR
# ============================================================================

cat("\n========================================\n")
cat("  META-ANALYSIS: OBJECTIVE RESPONSE RATE (ORR)\n")
cat("========================================\n\n")

meta_orr <- metabin(
  event.e = orr_data$responders_exp, n.e = orr_data$n_exp,
  event.c = orr_data$responders_ctrl, n.c = orr_data$n_ctrl,
  studlab = orr_data$study, sm = "RR", method = "MH",
  method.tau = "DL", hakn = FALSE,
  title = "Objective Response Rate (ORR)",
  comb.fixed = TRUE, comb.random = TRUE
)
print(summary(meta_orr))

# ORR Forest Plot (k=5, full columns, wide layout)
png(file.path(output_dir, "forest_ORR.png"), width = 1800, height = 800, res = 150)
forest(meta_orr,
       layout = "JAMA", fontsize = 10,
       leftcols = c("studlab", "event.e", "n.e", "event.c", "n.c",
                    "effect", "ci", "w.random"),
       leftlabs = c("Study", "Events(E)", "Total(E)", "Events(C)",
                    "Total(C)", "RR", "95% CI", "Weight"),
       rightcols = c("effect", "ci"),
       rightlabs = c("RR", "95% CI"),
       col.diamond = "darkgreen", col.square = "seagreen",
       col.square.lines = "seagreen4", col.inside = "white",
       print.I2 = TRUE, print.tau2 = TRUE, print.pval.Q = TRUE,
       prediction = FALSE,
       xlim = c(0.5, 6.0), at = c(0.5, 1.0, 2.0, 3.0, 4.0, 5.0, 6.0),
```

```
       smlab = "RR (95% CI)", ref = 1,
       label.left = "Favors Chemo",
       label.right = "Favors ICI + Chemo",
       main = "ORR: ICI + Chemo vs. Chemo Alone in Advanced BTC"
)
dev.off()
cat("  Saved: forest_ORR.png\n")

# ==============================================================================
# STEP 8: META-ANALYSIS FOR DCR
# ==============================================================================

cat("\n========================================\n")
cat("  META-ANALYSIS: DISEASE CONTROL RATE (DCR)\n")
cat("========================================\n\n")

meta_dcr <- metabin(
  event.e = dcr_data$dcr_exp, n.e = dcr_data$n_exp,
  event.c = dcr_data$dcr_ctrl, n.c = dcr_data$n_ctrl,
  studlab = dcr_data$study, sm = "RR", method = "MH",
  method.tau = "DL", hakn = FALSE,
  title = "Disease Control Rate (DCR)",
  comb.fixed = TRUE, comb.random = TRUE
)
print(summary(meta_dcr))

# DCR Forest Plot (k=5)
png(file.path(output_dir, "forest_DCR.png"), width = 1800, height = 800, res = 150)
forest(meta_dcr,
       layout = "JAMA", fontsize = 10,
       leftcols = c("studlab", "event.e", "n.e", "event.c", "n.c",
                    "effect", "ci", "w.random"),
       leftlabs = c("Study", "Events(E)", "Total(E)", "Events(C)",
                    "Total(C)", "RR", "95% CI", "Weight"),
       rightcols = c("effect", "ci"),
       rightlabs = c("RR", "95% CI"),
       col.diamond = "darkorange", col.square = "darkorange3",
       col.square.lines = "darkorange4", col.inside = "white",
       print.I2 = TRUE, print.tau2 = TRUE, print.pval.Q = TRUE,
       prediction = FALSE,
       xlim = c(0.5, 4.0), at = c(0.5, 0.75, 1.0, 1.5, 2.0, 3.0, 4.0),
       smlab = "RR (95% CI)", ref = 1,
       label.left = "Favors Chemo",
       label.right = "Favors ICI + Chemo",
       main = "DCR: ICI + Chemo vs. Chemo Alone in Advanced BTC"
)
dev.off()
cat("  Saved: forest_DCR.png\n")

# ==============================================================================
# STEP 9: META-ANALYSIS FOR AE (ANY GRADE)
# ==============================================================================

cat("\n========================================\n")
cat("  META-ANALYSIS: ADVERSE EVENTS (ANY GRADE)\n")
```

```
cat("========================================\n\n")

meta_ae_any <- metabin(
  event.e = ae_any_data$events_exp, n.e = ae_any_data$n_exp,
  event.c = ae_any_data$events_ctrl, n.c = ae_any_data$n_ctrl,
  studlab = ae_any_data$study, sm = "RR", method = "MH",
  method.tau = "DL", hakn = FALSE,
  title = "Adverse Events (Any Grade)",
  comb.fixed = TRUE, comb.random = TRUE
)
print(summary(meta_ae_any))

# AE Any Grade Forest Plot (k=2, REDUCED leftcols to fix overlap)
png(file.path(output_dir, "forest_AE_AnyGrade.png"), width = 1400, height = 480, res = 150)
forest(meta_ae_any,
       layout = "JAMA", fontsize = 10,
       leftcols = c("studlab", "event.e", "event.c", "effect", "ci", "w.random"),
       leftlabs = c("Study", "E-Events", "C-Events", "RR", "95% CI", "Weight"),
       rightcols = c("effect", "ci"),
       rightlabs = c("RR", "95% CI"),
       col.diamond = "darkorchid", col.square = "mediumorchid",
       col.square.lines = "darkorchid4", col.inside = "white",
       print.I2 = TRUE, print.tau2 = TRUE, print.pval.Q = TRUE,
       prediction = FALSE,
       xlim = c(0.5, 2.0), at = c(0.5, 0.75, 1.0, 1.25, 1.5, 2.0),
       ref = 1,
       label.left = "Favors ICI + Chemo",
       label.right = "Favors Chemo",
       main = "AE (Any Grade): ICI + Chemo vs. Chemo Alone"
)
dev.off()
cat("  Saved: forest_AE_AnyGrade.png\n")

# ============================================================================
# STEP 10: META-ANALYSIS FOR GRADE 3-4 AE
# ============================================================================

cat("\n========================================\n")
cat("  META-ANALYSIS: GRADE 3-4 ADVERSE EVENTS\n")
cat("========================================\n\n")

meta_g34 <- metabin(
  event.e = g34_ae_data$events_exp, n.e = g34_ae_data$n_exp,
  event.c = g34_ae_data$events_ctrl, n.c = g34_ae_data$n_ctrl,
  studlab = g34_ae_data$study, sm = "RR", method = "MH",
  method.tau = "DL", hakn = FALSE,
  title = "Grade 3-4 Adverse Events",
  comb.fixed = TRUE, comb.random = TRUE
)
print(summary(meta_g34))

# G3-4 AE Forest Plot (k=3, REDUCED leftcols + WIDENED xlim for Li 2023)
png(file.path(output_dir, "forest_G34_AE.png"), width = 1400, height = 520, res = 150)
forest(meta_g34,
       layout = "JAMA", fontsize = 10,
```

```
       leftcols = c("studlab", "event.e", "event.c", "effect", "ci", "w.random"),
       leftlabs = c("Study", "E-Events", "C-Events", "RR", "95% CI", "Weight"),
       rightcols = c("effect", "ci"),
       rightlabs = c("RR", "95% CI"),
       col.diamond = "purple", col.square = "mediumpurple",
       col.square.lines = "purple4", col.inside = "white",
       print.I2 = TRUE, print.tau2 = TRUE, print.pval.Q = TRUE,
       prediction = FALSE,
       xlim = c(0.1, 8.0), at = c(0.1, 0.5, 1.0, 2.0, 4.0, 6.0, 8.0),
       smlab = "RR (95% CI)", ref = 1,
       label.left = "Favors ICI + Chemo",
       label.right = "Favors Chemo",
       main = "Grade 3-4 AE: ICI + Chemo vs. Chemo Alone"
)
dev.off()
cat("  Saved: forest_G34_AE.png\n")

# ==============================================================================
# STEP 11: SUBGROUP ANALYSES
# ==============================================================================

cat("\n========================================\n")
cat("  SUBGROUP ANALYSES\n")
cat("========================================\n\n")

# ---- 11.1 ORR by Study Design ----
cat("--- ORR Subgroup: Study Design ---\n")
meta_orr_design <- update(meta_orr, subgroup = orr_data$phase,
                          print.subgroup.name = FALSE)
print(summary(meta_orr_design))

png(file.path(output_dir, "forest_ORR_subgroup_design.png"),
    width = 1800, height = 950, res = 150)
forest(meta_orr_design,
       layout = "JAMA", fontsize = 9,
       leftcols = c("studlab", "event.e", "n.e", "event.c", "n.c",
                    "effect", "ci", "w.random"),
       leftlabs = c("Study", "Events(E)", "Total(E)", "Events(C)",
                    "Total(C)", "RR", "95% CI", "Weight"),
       col.diamond = "darkgreen", col.square = "seagreen",
       col.square.lines = "seagreen4", col.inside = "white",
       print.I2 = TRUE, print.tau2 = TRUE, prediction = FALSE,
       xlim = c(0.5, 6.0), at = c(0.5, 1.0, 2.0, 3.0, 4.0, 5.0, 6.0),
       ref = 1,
       label.left = "Favors Chemo", label.right = "Favors ICI + Chemo",
       main = "ORR Subgroup: Phase III vs Single-center RCTs"
)
dev.off()
cat("  Saved: forest_ORR_subgroup_design.png\n")

# ---- 11.2 DCR by Study Design ----
cat("\n--- DCR Subgroup: Study Design ---\n")
meta_dcr_design <- update(meta_dcr, subgroup = dcr_data$phase,
                          print.subgroup.name = FALSE)
print(summary(meta_dcr_design))
```

```
png(file.path(output_dir, "forest_DCR_subgroup_design.png"),
    width = 1800, height = 950, res = 150)
forest(meta_dcr_design,
       layout = "JAMA", fontsize = 9,
       leftcols = c("studlab", "event.e", "n.e", "event.c", "n.c",
                    "effect", "ci", "w.random"),
       leftlabs = c("Study", "Events(E)", "Total(E)", "Events(C)",
                    "Total(C)", "RR", "95% CI", "Weight"),
       col.diamond = "darkorange", col.square = "darkorange3",
       col.square.lines = "darkorange4", col.inside = "white",
       print.I2 = TRUE, print.tau2 = TRUE, prediction = FALSE,
       xlim = c(0.5, 4.0), at = c(0.5, 0.75, 1.0, 1.5, 2.0, 3.0, 4.0),
       ref = 1,
       label.left = "Favors Chemo", label.right = "Favors ICI + Chemo",
       main = "DCR Subgroup: Phase III vs Single-center RCTs"
)
dev.off()
cat("  Saved: forest_DCR_subgroup_design.png\n")

# ---- 11.3 ORR by Chemo Regimen ----
cat("\n--- ORR Subgroup: Chemo Regimen ---\n")
meta_orr_chemo <- update(meta_orr, subgroup = orr_data$chemo_type,
                         print.subgroup.name = FALSE)
print(summary(meta_orr_chemo))

png(file.path(output_dir, "forest_ORR_subgroup_chemo.png"),
    width = 1800, height = 950, res = 150)
forest(meta_orr_chemo,
       layout = "JAMA", fontsize = 9,
       leftcols = c("studlab", "event.e", "n.e", "event.c", "n.c",
                    "effect", "ci", "w.random"),
       leftlabs = c("Study", "Events(E)", "Total(E)", "Events(C)",
                    "Total(C)", "RR", "95% CI", "Weight"),
       col.diamond = "darkgreen", col.square = "seagreen",
       col.square.lines = "seagreen4", col.inside = "white",
       print.I2 = TRUE, print.tau2 = TRUE, prediction = FALSE,
       xlim = c(0.5, 6.0), at = c(0.5, 1.0, 2.0, 3.0, 4.0, 5.0, 6.0),
       ref = 1,
       label.left = "Favors Chemo", label.right = "Favors ICI + Chemo",
       main = "ORR Subgroup: GemCis vs Non-GemCis Regimens"
)
dev.off()
cat("  Saved: forest_ORR_subgroup_chemo.png\n")

# ============================================================================
# STEP 12: SENSITIVITY ANALYSIS
# ============================================================================

cat("\n========================================\n")
cat("  SENSITIVITY ANALYSIS\n")
cat("========================================\n\n")

# ORR Leave-One-Out
cat("--- ORR Leave-One-Out ---\n")
```

```
meta_orr_loo <- metainf(meta_orr, pooled = "random")
print(meta_orr_loo)

png(file.path(output_dir, "sensitivity_ORR_loo.png"), width = 1400, height = 800, res = 150)
forest(metainf(meta_orr, pooled = "random"),
       layout = "JAMA", fontsize = 9,
       leftcols = c("studlab", "effect", "ci"),
       leftlabs = c("Omitted Study", "RR", "95% CI"),
       rightcols = c("effect", "ci"),
       rightlabs = c("RR", "95% CI"),
       col.diamond = "darkgreen", col.square = "seagreen",
       xlim = c(0.5, 3.0), at = c(0.5, 1.0, 1.5, 2.0, 2.5, 3.0),
       ref = 1,
       main = "ORR Sensitivity Analysis (Leave-One-Out)"
)
dev.off()
cat("  Saved: sensitivity_ORR_loo.png\n")

# DCR Leave-One-Out
cat("\n--- DCR Leave-One-Out ---\n")
meta_dcr_loo <- metainf(meta_dcr, pooled = "random")
print(meta_dcr_loo)

png(file.path(output_dir, "sensitivity_DCR_loo.png"), width = 1400, height = 800, res = 150)
forest(metainf(meta_dcr, pooled = "random"),
       layout = "JAMA", fontsize = 9,
       leftcols = c("studlab", "effect", "ci"),
       leftlabs = c("Omitted Study", "RR", "95% CI"),
       rightcols = c("effect", "ci"),
       rightlabs = c("RR", "95% CI"),
       col.diamond = "darkorange", col.square = "darkorange3",
       xlim = c(0.5, 3.0), at = c(0.5, 1.0, 1.5, 2.0, 2.5, 3.0),
       ref = 1,
       main = "DCR Sensitivity Analysis (Leave-One-Out)"
)
dev.off()
cat("  Saved: sensitivity_DCR_loo.png\n")

# OS and PFS Leave-One-Out (print only)
cat("\n--- OS Leave-One-Out ---\n")
print(metainf(meta_os, pooled = "fixed"))
cat("\n--- PFS Leave-One-Out ---\n")
print(metainf(meta_pfs, pooled = "random"))

# ============================================================================
# STEP 13: PUBLICATION BIAS ASSESSMENT
# ============================================================================

cat("\n========================================\n")
cat("  PUBLICATION BIAS ASSESSMENT\n")
cat("========================================\n\n")

# ---- ORR Funnel Plot (k=5) ----
cat("--- ORR Funnel Plot ---\n")
png(file.path(output_dir, "funnel_ORR.png"), width = 900, height = 800, res = 150)
```

```
funnel(meta_orr, studlab = TRUE, col = "seagreen",
       cex.studlab = 0.8, col.random = "darkgreen")
title("Funnel Plot: Objective Response Rate (ORR)", line = 2.5)
mtext("Note: k=5, limited power", side = 1, line = 3.8,
      adj = 0, cex = 0.75, col = "gray40")
tryCatch({
  egger_orr <- metabias(meta_orr, method.bias = "linreg")
  if (!is.null(egger_orr$p.value)) {
    mtext(paste0("Egger: p = ", format(egger_orr$p.value, digits = 3)),
          side = 1, line = 3.5, adj = 1, cex = 0.8, col = "darkgreen")
  }
}, error = function(e) cat("  ORR Egger test skipped\n"))
dev.off()
cat("  Saved: funnel_ORR.png\n")

# ORR Egger test
cat("\n--- ORR Egger Test ---\n")
tryCatch({
  print(metabias(meta_orr, method.bias = "linreg"))
}, error = function(e) cat("  Skipped\n"))

# ---- DCR Funnel Plot (k=5) ----
cat("\n--- DCR Funnel Plot ---\n")
png(file.path(output_dir, "funnel_DCR.png"), width = 900, height = 800, res = 150)
funnel(meta_dcr, studlab = TRUE, col = "darkorange3",
       cex.studlab = 0.8, col.random = "darkorange")
title("Funnel Plot: Disease Control Rate (DCR)", line = 2.5)
mtext("Note: k=5, limited power", side = 1, line = 3.8,
      adj = 0, cex = 0.75, col = "gray40")
# Manually add non-overlapping labels for the two large studies
# TOPAZ-1 and KEYNOTE-966 points overlap; use text with offset
tryCatch({
  # Get study-level data from the meta object
  # Add annotation for overlapping labels
  mtext("Oh 2024 / Kelley 2023 (near RR=1.0)", side = 3, line = 0.5,
        adj = 0.15, cex = 0.7, col = "darkorange4")
}, error = function(e) invisible())
tryCatch({
  egger_dcr <- metabias(meta_dcr, method.bias = "linreg")
  if (!is.null(egger_dcr$p.value)) {
    mtext(paste0("Egger: p = ", format(egger_dcr$p.value, digits = 3)),
          side = 1, line = 3.5, adj = 1, cex = 0.8, col = "darkorange")
  }
}, error = function(e) cat("  DCR Egger test skipped\n"))
dev.off()
cat("  Saved: funnel_DCR.png\n")

# ============================================================================
# STEP 14: EXPORT SUMMARY RESULTS
# ============================================================================

cat("\n========================================\n")
cat("  EXPORTING RESULTS\n")
cat("========================================\n\n")
```

```
os_p   <- get_pooled(meta_os, use_fixed = TRUE)
pfs_p  <- get_pooled(meta_pfs, use_fixed = FALSE)
orr_p  <- get_pooled(meta_orr, use_fixed = FALSE)
dcr_p  <- get_pooled(meta_dcr, use_fixed = FALSE)
ae_p   <- get_pooled(meta_ae_any, use_fixed = FALSE)
g34_p  <- get_pooled(meta_g34, use_fixed = FALSE)

results_summary <- data.frame(
  Outcome = c("Overall Survival (OS)", "Progression-Free Survival (PFS)",
              "Objective Response Rate (ORR)", "Disease Control Rate (DCR)",
              "Adverse Events (Any Grade)", "Grade 3-4 Adverse Events"),
  Studies = c(meta_os$k, meta_pfs$k, meta_orr$k, meta_dcr$k,
              meta_ae_any$k, meta_g34$k),
  Effect_Measure = c("HR", "HR", "RR", "RR", "RR", "RR"),
  Pooled_Estimate = c(round(exp(os_p$TE), 3), round(exp(pfs_p$TE), 3),
                      round(exp(orr_p$TE), 3), round(exp(dcr_p$TE), 3),
                      round(exp(ae_p$TE), 3), round(exp(g34_p$TE), 3)),
  CI_Lower = c(round(exp(os_p$lower), 3), round(exp(pfs_p$lower), 3),
               round(exp(orr_p$lower), 3), round(exp(dcr_p$lower), 3),
               round(exp(ae_p$lower), 3), round(exp(g34_p$lower), 3)),
  CI_Upper = c(round(exp(os_p$upper), 3), round(exp(pfs_p$upper), 3),
               round(exp(orr_p$upper), 3), round(exp(dcr_p$upper), 3),
               round(exp(ae_p$upper), 3), round(exp(g34_p$upper), 3)),
  P_value = c(formatC(os_p$pval, format = "e", digits = 2),
              formatC(pfs_p$pval, format = "e", digits = 2),
              formatC(orr_p$pval, format = "e", digits = 2),
              formatC(dcr_p$pval, format = "e", digits = 2),
              formatC(ae_p$pval, format = "e", digits = 2),
              formatC(g34_p$pval, format = "e", digits = 2)),
  I2 = c(paste0(round(meta_os$I2 * 100, 1), "%"),
         paste0(round(meta_pfs$I2 * 100, 1), "%"),
         paste0(round(meta_orr$I2 * 100, 1), "%"),
         paste0(round(meta_dcr$I2 * 100, 1), "%"),
         paste0(round(meta_ae_any$I2 * 100, 1), "%"),
         paste0(round(meta_g34$I2 * 100, 1), "%")),
  Tau2 = c(round(meta_os$tau2, 4), round(meta_pfs$tau2, 4),
           round(meta_orr$tau2, 4), round(meta_dcr$tau2, 4),
           round(meta_ae_any$tau2, 4), round(meta_g34$tau2, 4)),
  Heterogeneity_P = c(format(meta_os$pval.Q, digits = 3, nsmall = 3),
                      format(meta_pfs$pval.Q, digits = 3, nsmall = 3),
                      format(meta_orr$pval.Q, digits = 3, nsmall = 3),
                      format(meta_dcr$pval.Q, digits = 3, nsmall = 3),
                      format(meta_ae_any$pval.Q, digits = 3, nsmall = 3),
                      format(meta_g34$pval.Q, digits = 3, nsmall = 3)),
  Model = c(os_p$model, pfs_p$model, orr_p$model,
            dcr_p$model, ae_p$model, g34_p$model)
)

print(results_summary)
write.csv(results_summary, file.path(output_dir, "meta_analysis_summary.csv"),
          row.names = FALSE, fileEncoding = "UTF-8")
cat("  Saved: meta_analysis_summary.csv\n")

# Individual study results
os_ind <- data.frame(Study = os_data$study, Outcome = "OS", Measure = "HR",
```

```
                     Effect = os_data$hr, N_Exp = os_data$n_exp, N_Ctrl = os_data$n_ctrl,
                     Weight = round(meta_os$w.fixed / sum(meta_os$w.fixed) * 100, 1))
pfs_ind <- data.frame(Study = pfs_data$study, Outcome = "PFS", Measure = "HR",
                      Effect = pfs_data$hr, N_Exp = pfs_data$n_exp, N_Ctrl = pfs_data$n_ctrl,
                      Weight = round(meta_pfs$w.random / sum(meta_pfs$w.random, na.rm = TRUE) * 100, 1))
orr_ind <- data.frame(Study = orr_data$study, Outcome = "ORR", Measure = "RR",
                      Effect = round(exp(meta_orr$TE), 3), N_Exp = orr_data$n_exp, N_Ctrl = orr_data$n_ctrl,
                      Weight = round(meta_orr$w.random / sum(meta_orr$w.random, na.rm = TRUE) * 100, 1))
dcr_ind <- data.frame(Study = dcr_data$study, Outcome = "DCR", Measure = "RR",
                      Effect = round(exp(meta_dcr$TE), 3), N_Exp = dcr_data$n_exp, N_Ctrl = dcr_data$n_ctrl,
                      Weight = round(meta_dcr$w.random / sum(meta_dcr$w.random, na.rm = TRUE) * 100, 1))

write.csv(rbind(os_ind, pfs_ind, orr_ind, dcr_ind),
          file.path(output_dir, "individual_study_results.csv"),
          row.names = FALSE, fileEncoding = "UTF-8")
cat("  Saved: individual_study_results.csv\n")

# ============================================================================
# STEP 15: GENERATE TEXT REPORT
# ============================================================================

cat("\n========================================\n")
cat("  GENERATING TEXT REPORT\n")
cat("========================================\n\n")

report_text <- paste0(
"==============================================================================\n",
"  META-ANALYSIS REPORT\n",
"  ICI + Chemotherapy vs. Chemotherapy Alone in Advanced BTC: 5 RCTs\n",
"  Method: DerSimonian-Laird (standard, no HK adjustment)\n",
"==============================================================================\n\n",
"STUDIES: 5 RCTs (N = 2,000)\n",
"  1. Oh 2024 TOPAZ-1         (Phase III, n = 685)\n",
"  2. Kelley 2023 KEYNOTE-966 (Phase III, n = 1,069)\n",
"  3. Cui 2023                (Single-center, n = 100)\n",
"  4. Deng 2023               (Single-center, n = 80)\n",
"  5. Li 2023                 (Single-center, n = 66)\n\n",
"==============================================================================\n",
"  RESULTS\n",
"==============================================================================\n\n",
"1. OVERALL SURVIVAL [2 RCTs, Fixed-effect]\n",
"   HR = ", round(exp(os_p$TE), 3), " (95% CI: ", round(exp(os_p$lower), 3),
" - ", round(exp(os_p$upper), 3), ")\n",
"   P = ", formatC(os_p$pval, format = "e", digits = 1),
", I2 = ", round(meta_os$I2 * 100, 1), "%\n",
"   -> Significant OS benefit\n\n",
"2. PROGRESSION-FREE SURVIVAL [2 RCTs, Random-effects]\n",
"   HR = ", round(exp(pfs_p$TE), 3), " (95% CI: ", round(exp(pfs_p$lower), 3),
" - ", round(exp(pfs_p$upper), 3), ")\n",
"   P = ", formatC(pfs_p$pval, format = "e", digits = 1),
", I2 = ", round(meta_pfs$I2 * 100, 1), "%\n",
"   -> Significant PFS benefit\n\n",
"3. OBJECTIVE RESPONSE RATE [5 RCTs, Random-effects]\n",
"   RR = ", round(exp(orr_p$TE), 3), " (95% CI: ", round(exp(orr_p$lower), 3),
" - ", round(exp(orr_p$upper), 3), ")\n",
```

```
"   P = ", formatC(orr_p$pval, format = "e", digits = 1),
", I2 = ", round(meta_orr$I2 * 100, 1), "%\n",
"   -> Significantly higher ORR\n\n",
"4. DISEASE CONTROL RATE [5 RCTs, Random-effects]\n",
"   RR = ", round(exp(dcr_p$TE), 3), " (95% CI: ", round(exp(dcr_p$lower), 3),
" - ", round(exp(dcr_p$upper), 3), ")\n",
"   P = ", formatC(dcr_p$pval, format = "e", digits = 1),
", I2 = ", round(meta_dcr$I2 * 100, 1), "%\n",
"   -> Trend toward benefit; high heterogeneity\n\n",
"5. ADVERSE EVENTS (ANY GRADE) [2 RCTs]\n",
"   RR = ", round(exp(ae_p$TE), 3), " (95% CI: ", round(exp(ae_p$lower), 3),
" - ", round(exp(ae_p$upper), 3), ")\n",
"   P = ", formatC(ae_p$pval, format = "e", digits = 1),
", I2 = ", round(meta_ae_any$I2 * 100, 1), "%\n",
"   -> No difference\n\n",
"6. GRADE 3-4 ADVERSE EVENTS [3 RCTs]\n",
"   RR = ", round(exp(g34_p$TE), 3), " (95% CI: ", round(exp(g34_p$lower), 3),
" - ", round(exp(g34_p$upper), 3), ")\n",
"   P = ", formatC(g34_p$pval, format = "e", digits = 1),
", I2 = ", round(meta_g34$I2 * 100, 1), "%\n",
"   -> No significant difference\n\n",
"================================================================================\n",
"  CONCLUSION\n",
"================================================================================\n\n",
"ICI + chemotherapy significantly improved OS, PFS, and ORR vs.\n",
"chemotherapy alone in advanced BTC, without increasing G3-4 AE.\n\n",
"Output: ", normalizePath(output_dir), "\n",
"Generated: ", format(Sys.time(), "%Y-%m-%d %H:%M:%S"), "\n",
"================================================================================"
)

cat(report_text)
writeLines(report_text, file.path(output_dir, "meta_analysis_report.txt"))
cat("\n  Saved: meta_analysis_report.txt\n")

# ============================================================================
# STEP 16: PRINT FILE LIST
# ============================================================================

cat("\n========================================\n")
cat("  ALL FILES GENERATED\n")
cat("========================================\n\n")
cat("Directory: ", normalizePath(output_dir), "\n\n")
for (f in sort(list.files(output_dir, pattern = "\\.(png|csv|txt)$"))) {
  cat("  [OK] ", f, "\n")
}
cat("\n===== META-ANALYSIS COMPLETE =====\n")
```

Supplementary Code 2: Retrospective Meta-Analysis

File: Supplementary_Code_2_Retrospective_Meta_Analysis.R

Purpose: Meta-analysis of 6 retrospective studies

Corresponds to: Part II (retrospective component) of the manuscript, Figures 3, 5-11, 13-14

```
# ============================================================================
# Supplementary Code for:
#   PD-1/PD-L1 Inhibitors Combined with Chemotherapy as First-Line
#   Treatment for Advanced Biliary Tract Cancer:
#   A Systematic Review and Meta-Analysis
#
# Journal: Frontiers in Medicine
#
# File: Supplementary_Code_2_Retrospective_Meta_Analysis.R
#
# Description:
#   Meta-analysis of 6 retrospective studies evaluating PD-1/PD-L1 inhibitors
#
# Corresponds to: Part II (retrospective component): PFS, OS, ORR, DCR, and safety outcomes
#
# R version: 4.6.1
# Required packages:
#   - meta     (v8.5-0)
#   - metafor  (v5.0-1)
#   - ggplot2
#   - gridExtra
#   - grid
#
# Notes:
#   - All study data are embedded directly in the script for full reproducibility
#   - Figures are saved as both PNG (300 dpi) and PDF formats
#   - Run the script from top to bottom to reproduce all analyses
#
# ============================================================================

# Step 1: Install and load required packages
# ----------------------------------------------------------------------------

packages <- c("meta", "metafor", "dmetar", "ggplot2", "gridExtra", "grid")

install_if_missing <- function(pkg) {
  if (!requireNamespace(pkg, quietly = TRUE)) {
    install.packages(pkg, repos = "https://cran.r-project.org")
  }
}

invisible(sapply(packages, install_if_missing))

library(meta)
```

```
library(metafor)
library(ggplot2)
library(gridExtra)
library(grid)

# Set output directory
output_dir <- "./meta_analysis_output"
dir.create(output_dir, showWarnings = FALSE, recursive = TRUE)

# ==============================================================================
# Step 2: Helper function - calculate SE from HR and 95% CI
# ==============================================================================

calc_se_loghr <- function(lower, upper) {
  (log(upper) - log(lower)) / (2 * 1.96)
}

# ==============================================================================
# Step 3: Embed all study data directly
# ==============================================================================

# ---- 3.1 PFS data (HR with 95% CI) ----
pfs_data <- data.frame(
  study      = c("Zhao 2021", "Lu 2024", "Madzikatire 2024",
                 "Rimini 2024", "Zhang 2025", "Zhao 2025"),
  hr         = c(0.62, 0.65, 0.48, 0.57, 0.393, 0.46),
  lower      = c(0.39, 0.48, 0.27, 0.47, 0.278, 0.38),
  upper      = c(0.94, 0.88, 0.87, 0.70, 0.555, 0.54),
  p_value    = c(0.0306, 0.005, 0.002, 0.0001, 0.001, 0.017),
  n_exp      = c(45, 93, 51, 350, 62, 93),
  n_ctrl     = c(45, 93, 30, 213, 88, 90),
  tumor_type = c("Mixed BTC", "Mixed BTC", "iCCA", "Mixed BTC",
                 "GBC", "Mixed BTC"),
  country    = c("China", "China", "China", "Italy", "China", "China"),
  design     = c("Single-center", "Single-center", "Single-center",
                 "Multi-center", "Single-center", "Single-center")
)

# ---- 3.2 OS data (HR with 95% CI) ----
os_data <- data.frame(
  study      = c("Zhao 2021", "Lu 2024", "Madzikatire 2024",
                 "Rimini 2024", "Zhang 2025", "Zhao 2025"),
  hr         = c(0.93, 0.91, 0.53, 0.63, 0.530, 0.39),
  lower      = c(0.57, 0.62, 0.30, 0.50, 0.357, 0.32),
  upper      = c(1.50, 1.32, 0.94, 0.80, 0.786, 0.47),
  p_value    = c(0.765, 0.608, 0.008, 0.0002, 0.001, 0.033),
  n_exp      = c(45, 93, 51, 350, 62, 93),
  n_ctrl     = c(45, 93, 30, 213, 88, 90),
  tumor_type = c("Mixed BTC", "Mixed BTC", "iCCA", "Mixed BTC",
                 "GBC", "Mixed BTC"),
  country    = c("China", "China", "China", "Italy", "China", "China"),
  design     = c("Single-center", "Single-center", "Single-center",
                 "Multi-center", "Single-center", "Single-center")
)
```

```
# ---- 3.3 ORR data (responders / total for 2x2 table) ----
# Studies with exact responder counts
orr_data <- data.frame(
  study      = c("Zhao 2021", "Lu 2024", "Madzikatire 2024",
                 "Zhang 2025", "Zhao 2025"),
  responders_exp = c(17, 18, 15, 25, 38),
  n_exp          = c(45, 93, 51, 62, 93),
  responders_ctrl = c(5, 7, 4, 18, 23),
  n_ctrl         = c(45, 90, 30, 88, 90),
  p_value        = c(0.003, NA, NA, 0.008, 0.007),
  tumor_type     = c("Mixed BTC", "Mixed BTC", "iCCA", "GBC", "Mixed BTC")
)
# Note: Lu 2024 ORR 19.1%(18/93); Madzikatire 2024 ORR 29.4%(15/51)
#       Lu 2024 control ORR 7.8%(7/90); Madzikatire 2024 control ORR 13.3%(4/30)

# ---- 3.4 DCR data ----
# Lu 2024: DCR estimated from percentages (68.5% x 93 ~= 64; 53.3% x 90 ~= 48)
dcr_data <- data.frame(
  study      = c("Lu 2024", "Madzikatire 2024",
                 "Zhang 2025", "Zhao 2025"),
  dcr_exp    = c(64, 40, 52, 78),
  n_exp      = c(93, 51, 62, 93),
  dcr_ctrl   = c(48, 22, 59, 56),
  n_ctrl     = c(90, 30, 88, 90),
  p_value    = c(NA, NA, 0.021, 0.001),
  tumor_type = c("Mixed BTC", "iCCA", "GBC", "Mixed BTC")
)

# ---- 3.5 Grade >=3 TRAEs data ----
# Zhang 2025: grade >=3 AE 58.06% x 62 ~= 36, 40.32% x 88 ~= 35
trae3_data <- data.frame(
  study      = c("Zhao 2021", "Madzikatire 2024", "Zhang 2025", "Zhao 2025"),
  events_exp = c(32, 34, 36, 31),
  n_exp      = c(45, 51, 62, 93),
  events_ctrl = c(29, 7, 35, 23),
  n_ctrl     = c(45, 30, 88, 90),
  tumor_type = c("Mixed BTC", "iCCA", "GBC", "Mixed BTC")
)

# ---- 3.6 Subgroup: China vs Non-China ----
pfs_data$region <- ifelse(pfs_data$country == "China", "China", "Non-China")
os_data$region  <- ifelse(os_data$country == "China", "China", "Non-China")

# ---- 3.7 Subgroup: Single-center vs Multi-center ----
pfs_data$center <- ifelse(pfs_data$design == "Multi-center", "Multi-center", "Single-center")
os_data$center  <- ifelse(os_data$design == "Multi-center", "Multi-center", "Single-center")

# ============================================================================
# STEP 4: META-ANALYSIS FOR PFS (Primary Endpoint)
# ============================================================================

cat("\n========================================\n")
cat("  META-ANALYSIS: PFS\n")
cat("========================================\n\n")
```

```
pfs_data$se <- calc_se_loghr(pfs_data$lower, pfs_data$upper)
pfs_data$loghr <- log(pfs_data$hr)

meta_pfs <- metagen(
  TE         = pfs_data$loghr,
  seTE       = pfs_data$se,
  studlab    = pfs_data$study,
  sm         = "HR",
  method.tau = "REML",
  hakn       = TRUE,
  title      = "Progression-Free Survival (PFS)"
)

print(summary(meta_pfs))

# ---- PFS Forest Plot ----
png(file.path(output_dir, "forest_PFS.png"), width = 1300, height = 750, res = 150)
forest(meta_pfs,
       layout      = "JAMA",
       fontsize    = 10,
       leftcols    = c("studlab", "effect", "ci", "w.random"),
       leftlabs    = c("Study", "HR", "95% CI", "Weight"),
       rightcols   = c("effect", "ci"),
       rightlabs   = c("HR", "95% CI"),
       col.diamond = "darkblue",
       col.square  = "steelblue",
       col.square.lines = "steelblue4",
       col.inside  = "white",
       print.I2    = TRUE,
       print.tau2  = TRUE,
       print.pval.Q = TRUE,
       prediction  = TRUE,
       xlim        = c(0.1, 2.0),
       at          = c(0.25, 0.5, 0.75, 1.0, 1.5, 2.0),
       label.left  = "Favors ICI+Chemo",
       label.right = "Favors Chemo",
       main        = "PFS: ICI + Chemotherapy vs. Chemotherapy Alone"
)
dev.off()
cat("  Saved: forest_PFS.png\n")

# ---- PFS Funnel Plot ----
png(file.path(output_dir, "funnel_PFS.png"), width = 900, height = 800, res = 150)
funnel(meta_pfs, studlab = TRUE, col = "steelblue",
       cex.studlab = 0.85, col.random = "darkblue")
title("Funnel Plot: Progression-Free Survival", line = 2.5)
# Add Egger test p-value
tryCatch({
  egger_pfs <- metabias(meta_pfs, method.bias = "linreg")
  if (!is.null(egger_pfs$p.value)) {
    mtext(paste0("Egger's test: p = ", format(egger_pfs$p.value, digits = 3)),
          side = 1, line = 3.5, adj = 1, cex = 0.8, col = "darkblue")
  }
}, error = function(e) cat("  PFS Egger test skipped (", conditionMessage(e), ")\n"))
dev.off()
```

```
cat("  Saved: funnel_PFS.png\n")

# ---- PFS Egger test print ----
cat("\n--- PFS Publication Bias (Egger Test) ---\n")
tryCatch({
  egger_pfs <- metabias(meta_pfs, method.bias = "linreg")
  print(egger_pfs)
}, error = function(e) {
  cat("  Egger test requires sufficient studies.\n")
})

# ============================================================================
# STEP 5: META-ANALYSIS FOR OS (Primary Endpoint)
# ============================================================================

cat("\n========================================\n")
cat("  META-ANALYSIS: OS\n")
cat("========================================\n\n")

os_data$se <- calc_se_loghr(os_data$lower, os_data$upper)
os_data$loghr <- log(os_data$hr)

meta_os <- metagen(
  TE         = os_data$loghr,
  seTE       = os_data$se,
  studlab    = os_data$study,
  sm         = "HR",
  method.tau = "REML",
  hakn       = TRUE,
  title      = "Overall Survival (OS)"
)

print(summary(meta_os))

# ---- OS Forest Plot ----
png(file.path(output_dir, "forest_OS.png"), width = 1300, height = 750, res = 150)
forest(meta_os,
       layout      = "JAMA",
       fontsize    = 10,
       leftcols    = c("studlab", "effect", "ci", "w.random"),
       leftlabs    = c("Study", "HR", "95% CI", "Weight"),
       rightcols   = c("effect", "ci"),
       rightlabs   = c("HR", "95% CI"),
       col.diamond = "darkred",
       col.square  = "indianred",
       col.square.lines = "indianred4",
       col.inside  = "white",
       print.I2    = TRUE,
       print.tau2  = TRUE,
       print.pval.Q = TRUE,
       prediction  = TRUE,
       xlim        = c(0.1, 2.0),
       at          = c(0.25, 0.5, 0.75, 1.0, 1.5, 2.0),
       label.left  = "Favors ICI+Chemo",
       label.right = "Favors Chemo",
```

```
       main        = "OS: ICI + Chemotherapy vs. Chemotherapy Alone"
)
dev.off()
cat("  Saved: forest_OS.png\n")

# ---- OS Funnel Plot ----
png(file.path(output_dir, "funnel_OS.png"), width = 900, height = 800, res = 150)
funnel(meta_os, studlab = TRUE, col = "indianred",
       cex.studlab = 0.85, col.random = "darkred")
title("Funnel Plot: Overall Survival", line = 2.5)
tryCatch({
  egger_os <- metabias(meta_os, method.bias = "linreg")
  if (!is.null(egger_os$p.value)) {
    mtext(paste0("Egger's test: p = ", format(egger_os$p.value, digits = 3)),
          side = 1, line = 3.5, adj = 1, cex = 0.8, col = "darkred")
  }
}, error = function(e) cat("  OS Egger test skipped (", conditionMessage(e), ")\n"))
dev.off()
cat("  Saved: funnel_OS.png\n")

# ---- OS Egger test print ----
cat("\n--- OS Publication Bias (Egger Test) ---\n")
tryCatch({
  egger_os <- metabias(meta_os, method.bias = "linreg")
  print(egger_os)
}, error = function(e) {
  cat("  Egger test error: ", conditionMessage(e), "\n")
})

# ============================================================================
# STEP 6: META-ANALYSIS FOR ORR (Relative Risk)
# ============================================================================

cat("\n========================================\n")
cat("  META-ANALYSIS: ORR\n")
cat("========================================\n\n")

meta_orr <- metabin(
  event.e     = orr_data$responders_exp,
  n.e         = orr_data$n_exp,
  event.c     = orr_data$responders_ctrl,
  n.c         = orr_data$n_ctrl,
  studlab     = orr_data$study,
  sm          = "RR",
  method      = "Inverse",
  method.tau  = "REML",
  hakn        = TRUE,
  title       = "Objective Response Rate (ORR)"
)

print(summary(meta_orr))

# ---- ORR Forest Plot ----
png(file.path(output_dir, "forest_ORR.png"), width = 1400, height = 650, res = 150)
forest(meta_orr,
```

```
       layout      = "JAMA",
       fontsize    = 10,
       leftcols    = c("studlab", "event.e", "n.e", "event.c", "n.c", "effect", "ci", "w.random"),
       leftlabs    = c("Study", "Events(E)", "Total(E)", "Events(C)", "Total(C)", "RR", "95% CI", "Weight"),
       rightcols   = c("effect", "ci"),
       rightlabs   = c("RR", "95% CI"),
       col.diamond = "darkgreen",
       col.square  = "seagreen",
       col.square.lines = "seagreen4",
       col.inside  = "white",
       print.I2    = TRUE,
       print.tau2  = TRUE,
       print.pval.Q = TRUE,
       prediction  = TRUE,
       xlim        = c(0.5, 5.0),
       at          = c(0.5, 1.0, 2.0, 3.0, 4.0),
       label.left  = "Favors ICI+Chemo",
       label.right = "Favors Chemo",
       main        = "ORR: ICI + Chemotherapy vs. Chemotherapy Alone"
)
dev.off()
cat("  Saved: forest_ORR.png\n")

# ============================================================================
# STEP 7: META-ANALYSIS FOR DCR (Relative Risk)
# ============================================================================

cat("\n========================================\n")
cat("  META-ANALYSIS: DCR\n")
cat("========================================\n\n")

meta_dcr <- metabin(
  event.e    = dcr_data$dcr_exp,
  n.e        = dcr_data$n_exp,
  event.c    = dcr_data$dcr_ctrl,
  n.c        = dcr_data$n_ctrl,
  studlab    = dcr_data$study,
  sm         = "RR",
  method     = "Inverse",
  method.tau = "REML",
  hakn       = TRUE,
  title      = "Disease Control Rate (DCR)"
)

print(summary(meta_dcr))

# ---- DCR Forest Plot ----
png(file.path(output_dir, "forest_DCR.png"), width = 1400, height = 600, res = 150)
forest(meta_dcr,
       layout      = "JAMA",
       fontsize    = 10,
       leftcols    = c("studlab", "event.e", "n.e", "event.c", "n.c", "effect", "ci", "w.random"),
       leftlabs    = c("Study", "Events(E)", "Total(E)", "Events(C)", "Total(C)", "RR", "95% CI", "Weight"),
       rightcols   = c("effect", "ci"),
       rightlabs   = c("RR", "95% CI"),
```

```
       col.diamond = "darkorange",
       col.square  = "darkorange3",
       col.square.lines = "darkorange4",
       col.inside  = "white",
       print.I2    = TRUE,
       print.tau2  = TRUE,
       print.pval.Q = TRUE,
       prediction  = TRUE,
       xlim        = c(0.5, 2.0),
       at          = c(0.5, 0.75, 1.0, 1.25, 1.5),
       label.left  = "Favors ICI+Chemo",
       label.right = "Favors Chemo",
       main        = "DCR: ICI + Chemotherapy vs. Chemotherapy Alone"
)
dev.off()
cat("  Saved: forest_DCR.png\n")

# ============================================================================
# STEP 8: META-ANALYSIS FOR Grade >=3 TRAEs (Safety)
# ============================================================================

cat("\n========================================\n")
cat("  META-ANALYSIS: Grade >=3 TRAEs\n")
cat("========================================\n\n")

meta_trae3 <- metabin(
  event.e    = trae3_data$events_exp,
  n.e        = trae3_data$n_exp,
  event.c    = trae3_data$events_ctrl,
  n.c        = trae3_data$n_ctrl,
  studlab    = trae3_data$study,
  sm         = "RR",
  method     = "Inverse",
  method.tau = "REML",
  hakn       = TRUE,
  title      = "Grade >=3 Treatment-Related Adverse Events"
)

print(summary(meta_trae3))

# ---- TRAEs Forest Plot ----
png(file.path(output_dir, "forest_TRAE3.png"), width = 1400, height = 600, res = 150)
forest(meta_trae3,
       layout      = "JAMA",
       fontsize    = 10,
       leftcols    = c("studlab", "event.e", "n.e", "event.c", "n.c", "effect", "ci", "w.random"),
       leftlabs    = c("Study", "Events(E)", "Total(E)", "Events(C)", "Total(C)", "RR", "95% CI", "Weight"),
       rightcols   = c("effect", "ci"),
       rightlabs   = c("RR", "95% CI"),
       col.diamond = "purple",
       col.square  = "mediumpurple",
       col.square.lines = "purple4",
       col.inside  = "white",
       print.I2    = TRUE,
       print.tau2  = TRUE,
```

```
       print.pval.Q = TRUE,
       prediction  = TRUE,
       xlim        = c(0.5, 4.0),
       at          = c(0.5, 1.0, 1.5, 2.0, 3.0),
       label.left  = "Favors ICI+Chemo",
       label.right = "Favors Chemo",
       main        = "Grade >=3 TRAEs: ICI + Chemo vs. Chemo Alone"
)
dev.off()
cat("  Saved: forest_TRAE3.png\n")

# ============================================================================
# STEP 9: SUBGROUP ANALYSIS
# ============================================================================

cat("\n========================================\n")
cat("  SUBGROUP ANALYSIS\n")
cat("========================================\n\n")

# ---- 9.1 PFS by Tumor Type ----
cat("--- PFS by Tumor Type ---\n")
meta_pfs_tumor <- update(meta_pfs,
                         subgroup = pfs_data$tumor_type,
                         print.subgroup.name = FALSE)
print(summary(meta_pfs_tumor))

png(file.path(output_dir, "forest_PFS_subgroup_tumor.png"), width = 1400, height = 850, res = 150)
forest(meta_pfs_tumor,
       layout      = "JAMA",
       fontsize    = 9,
       leftcols    = c("studlab", "effect", "ci", "w.random"),
       leftlabs    = c("Study", "HR", "95% CI", "Weight"),
       col.diamond = "darkblue",
       col.square  = "steelblue",
       col.square.lines = "steelblue4",
       col.inside  = "white",
       print.I2    = TRUE,
       print.tau2  = TRUE,
       xlim        = c(0.1, 2.0),
       at          = c(0.25, 0.5, 0.75, 1.0, 1.5, 2.0),
       label.left  = "Favors ICI+Chemo",
       label.right = "Favors Chemo",
       main        = "PFS Subgroup Analysis by Tumor Type"
)
dev.off()
cat("  Saved: forest_PFS_subgroup_tumor.png\n")

# ---- 9.2 OS by Tumor Type ----
cat("\n--- OS by Tumor Type ---\n")
meta_os_tumor <- update(meta_os,
                        subgroup = os_data$tumor_type,
                        print.subgroup.name = FALSE)
print(summary(meta_os_tumor))

png(file.path(output_dir, "forest_OS_subgroup_tumor.png"), width = 1400, height = 850, res = 150)
```

```
forest(meta_os_tumor,
       layout      = "JAMA",
       fontsize    = 9,
       leftcols    = c("studlab", "effect", "ci", "w.random"),
       leftlabs    = c("Study", "HR", "95% CI", "Weight"),
       col.diamond = "darkred",
       col.square  = "indianred",
       col.square.lines = "indianred4",
       col.inside  = "white",
       print.I2    = TRUE,
       print.tau2  = TRUE,
       xlim        = c(0.1, 2.0),
       at          = c(0.25, 0.5, 0.75, 1.0, 1.5, 2.0),
       label.left  = "Favors ICI+Chemo",
       label.right = "Favors Chemo",
       main        = "OS Subgroup Analysis by Tumor Type"
)
dev.off()
cat("  Saved: forest_OS_subgroup_tumor.png\n")

# ---- 9.3 PFS by Region (China vs Non-China) ----
cat("\n--- PFS by Region ---\n")
meta_pfs_region <- update(meta_pfs,
                          subgroup = pfs_data$region,
                          print.subgroup.name = FALSE)
print(summary(meta_pfs_region))

png(file.path(output_dir, "forest_PFS_subgroup_region.png"), width = 1400, height = 750, res = 150)
forest(meta_pfs_region,
       layout      = "JAMA",
       fontsize    = 10,
       leftcols    = c("studlab", "effect", "ci", "w.random"),
       leftlabs    = c("Study", "HR", "95% CI", "Weight"),
       col.diamond = "darkblue",
       col.square  = "steelblue",
       print.I2    = TRUE,
       print.tau2  = TRUE,
       xlim        = c(0.1, 2.0),
       at          = c(0.25, 0.5, 0.75, 1.0, 1.5, 2.0),
       label.left  = "Favors ICI+Chemo",
       label.right = "Favors Chemo",
       main        = "PFS Subgroup Analysis by Region"
)
dev.off()
cat("  Saved: forest_PFS_subgroup_region.png\n")

# ---- 9.4 OS by Region ----
cat("\n--- OS by Region ---\n")
meta_os_region <- update(meta_os,
                         subgroup = os_data$region,
                         print.subgroup.name = FALSE)
print(summary(meta_os_region))

png(file.path(output_dir, "forest_OS_subgroup_region.png"), width = 1400, height = 750, res = 150)
forest(meta_os_region,
```

```
       layout      = "JAMA",
       fontsize    = 10,
       leftcols    = c("studlab", "effect", "ci", "w.random"),
       leftlabs    = c("Study", "HR", "95% CI", "Weight"),
       col.diamond = "darkred",
       col.square  = "indianred",
       print.I2    = TRUE,
       print.tau2  = TRUE,
       xlim        = c(0.1, 2.0),
       at          = c(0.25, 0.5, 0.75, 1.0, 1.5, 2.0),
       label.left  = "Favors ICI+Chemo",
       label.right = "Favors Chemo",
       main        = "OS Subgroup Analysis by Region"
)
dev.off()
cat("  Saved: forest_OS_subgroup_region.png\n")

# ---- 9.5 PFS by Study Design ----
cat("\n--- PFS by Study Design ---\n")
meta_pfs_design <- update(meta_pfs,
                          subgroup = pfs_data$center,
                          print.subgroup.name = FALSE)
print(summary(meta_pfs_design))

png(file.path(output_dir, "forest_PFS_subgroup_design.png"), width = 1400, height = 750, res = 150)
forest(meta_pfs_design,
       layout      = "JAMA",
       fontsize    = 10,
       leftcols    = c("studlab", "effect", "ci", "w.random"),
       leftlabs    = c("Study", "HR", "95% CI", "Weight"),
       col.diamond = "darkblue",
       col.square  = "steelblue",
       print.I2    = TRUE,
       print.tau2  = TRUE,
       xlim        = c(0.1, 2.0),
       at          = c(0.25, 0.5, 0.75, 1.0, 1.5, 2.0),
       label.left  = "Favors ICI+Chemo",
       label.right = "Favors Chemo",
       main        = "PFS Subgroup Analysis by Study Design"
)
dev.off()
cat("  Saved: forest_PFS_subgroup_design.png\n")

# ============================================================================
# STEP 10: SENSITIVITY ANALYSIS (Leave-One-Out)
# ============================================================================

cat("\n========================================\n")
cat("  SENSITIVITY ANALYSIS\n")
cat("========================================\n\n")

# ---- 10.1 PFS Leave-One-Out ----
cat("--- PFS Leave-One-Out ---\n")
meta_pfs_loo <- metainf(meta_pfs, pooled = "random")
print(meta_pfs_loo)
```

```
png(file.path(output_dir, "sensitivity_PFS_loo.png"), width = 1300, height = 700, res = 150)
forest(metainf(meta_pfs, pooled = "random"),
       layout = "JAMA",
       leftcols = c("studlab", "effect", "ci"),
       leftlabs = c("Omitted Study", "HR", "95% CI"),
       rightcols = c("effect", "ci"),
       rightlabs = c("HR", "95% CI"),
       col.diamond = "darkblue",
       col.square = "steelblue",
       xlim = c(0.2, 1.5),
       at = c(0.25, 0.5, 0.75, 1.0, 1.5),
       main = "PFS Sensitivity Analysis (Leave-One-Out)"
)
dev.off()
cat("  Saved: sensitivity_PFS_loo.png\n")

# ---- 10.2 OS Leave-One-Out ----
cat("\n--- OS Leave-One-Out ---\n")
meta_os_loo <- metainf(meta_os, pooled = "random")
print(meta_os_loo)

png(file.path(output_dir, "sensitivity_OS_loo.png"), width = 1300, height = 700, res = 150)
forest(metainf(meta_os, pooled = "random"),
       layout = "JAMA",
       leftcols = c("studlab", "effect", "ci"),
       leftlabs = c("Omitted Study", "HR", "95% CI"),
       rightcols = c("effect", "ci"),
       rightlabs = c("HR", "95% CI"),
       col.diamond = "darkred",
       col.square = "indianred",
       xlim = c(0.2, 1.5),
       at = c(0.25, 0.5, 0.75, 1.0, 1.5),
       main = "OS Sensitivity Analysis (Leave-One-Out)"
)
dev.off()
cat("  Saved: sensitivity_OS_loo.png\n")

# ============================================================================
# STEP 11: TRIM AND FILL ANALYSIS (Publication Bias)
# ============================================================================

cat("\n========================================\n")
cat("  TRIM AND FILL ANALYSIS\n")
cat("========================================\n\n")

# ---- 11.1 PFS Trim and Fill ----
cat("--- PFS Trim and Fill ---\n")
tryCatch({
  # Convert to rma object for trimfill
  rma_pfs <- rma.uni(yi = pfs_data$loghr, sei = pfs_data$se,
                     method = "REML", slab = pfs_data$study)
  tf_pfs <- trimfill(rma_pfs)
  print(tf_pfs)
```

```
  png(file.path(output_dir, "trimfill_PFS.png"), width = 900, height = 800, res = 150)
  funnel(tf_pfs, studlab = TRUE, cex.studlab = 0.85)
  title("Trim-and-Fill: PFS", line = 2.5)
  if (!is.null(tf_pfs$k0) && tf_pfs$k0 > 0) {
    mtext(paste0("Imputed studies: ", tf_pfs$k0),
          side = 1, line = 3.5, adj = 1, cex = 0.8, col = "darkblue")
  }
  dev.off()
  cat("  Saved: trimfill_PFS.png\n")
}, error = function(e) {
  cat("  PFS Trim-and-Fill skipped: ", conditionMessage(e), "\n")
})

# ---- 11.2 OS Trim and Fill ----
cat("\n--- OS Trim and Fill ---\n")
tryCatch({
  rma_os <- rma.uni(yi = os_data$loghr, sei = os_data$se,
                    method = "REML", slab = os_data$study)
  tf_os <- trimfill(rma_os)
  print(tf_os)

  png(file.path(output_dir, "trimfill_OS.png"), width = 900, height = 800, res = 150)
  funnel(tf_os, studlab = TRUE, cex.studlab = 0.85)
  title("Trim-and-Fill: OS", line = 2.5)
  if (!is.null(tf_os$k0) && tf_os$k0 > 0) {
    mtext(paste0("Imputed studies: ", tf_os$k0),
          side = 1, line = 3.5, adj = 1, cex = 0.8, col = "darkred")
  }
  dev.off()
  cat("  Saved: trimfill_OS.png\n")
}, error = function(e) {
  cat("  OS Trim-and-Fill skipped: ", conditionMessage(e), "\n")
})

# ============================================================================
# STEP 12: META-REGRESSION ANALYSIS
# ============================================================================

cat("\n========================================\n")
cat("  META-REGRESSION ANALYSIS\n")
cat("========================================\n\n")

# ---- 12.1 PFS Meta-regression: Sample size ----
cat("--- PFS Meta-regression: Sample Size ---\n")
tryCatch({
  rma_pfs_n <- rma.uni(yi = pfs_data$loghr, sei = pfs_data$se,
                       mods = ~ pfs_data$n_exp + pfs_data$n_ctrl,
                       method = "REML")
  print(rma_pfs_n)
}, error = function(e) {
  cat("  PFS meta-regression skipped: ", conditionMessage(e), "\n")
})

# ---- 12.2 OS Meta-regression: Year of publication ----
cat("\n--- OS Meta-regression: Publication Year ---\n")
```

```
tryCatch({
  pub_year <- c(2021, 2024, 2024, 2024, 2025, 2025)
  rma_os_yr <- rma.uni(yi = os_data$loghr, sei = os_data$se,
                       mods = ~ pub_year, method = "REML")
  print(rma_os_yr)
}, error = function(e) {
  cat("  OS meta-regression skipped: ", conditionMessage(e), "\n")
})

# ---- 12.3 OS Meta-regression: Multi-center vs Single-center ----
cat("\n--- OS Meta-regression: Study Design ---\n")
tryCatch({
  is_multicenter <- ifelse(os_data$design == "Multi-center", 1, 0)
  rma_os_design <- rma.uni(yi = os_data$loghr, sei = os_data$se,
                           mods = ~ is_multicenter, method = "REML")
  print(rma_os_design)
}, error = function(e) {
  cat("  OS meta-regression (design) skipped: ", conditionMessage(e), "\n")
})

# ============================================================================
# STEP 13: BUBBLE PLOT (Meta-regression Visualization)
# ============================================================================

cat("\n========================================\n")
cat("  BUBBLE PLOTS\n")
cat("========================================\n\n")

# ---- 13.1 PFS Bubble Plot by Total Sample Size ----
png(file.path(output_dir, "bubble_PFS_samplesize.png"), width = 900, height = 700, res = 150)
tryCatch({
  total_n <- pfs_data$n_exp + pfs_data$n_ctrl
  rma_pfs_bubble <- rma.uni(yi = pfs_data$loghr, sei = pfs_data$se,
                            mods = ~ total_n, method = "REML")
  bubble_plot_data <- data.frame(
    total_n = total_n,
    loghr = pfs_data$loghr,
    study = pfs_data$study,
    se = pfs_data$se
  )

  # Create bubble plot with ggplot2
  p <- ggplot(bubble_plot_data, aes(x = total_n, y = exp(loghr))) +
    geom_point(aes(size = 1/se^2), color = "steelblue", alpha = 0.7) +
    geom_text(aes(label = study), vjust = -1.2, size = 3) +
    geom_hline(yintercept = 1, linetype = "dashed", color = "gray50") +
    scale_size_continuous(name = "Precision (1/SE^2)", range = c(3, 15)) +
    labs(x = "Total Sample Size",
         y = "Hazard Ratio",
         title = "PFS: Effect Size by Sample Size") +
    theme_minimal(base_size = 12) +
    theme(legend.position = "bottom")
  print(p)
}, error = function(e) {
  cat("  Bubble plot error: ", conditionMessage(e), "\n")
```

```
  # Fallback simple plot
  total_n <- pfs_data$n_exp + pfs_data$n_ctrl
  plot(total_n, pfs_data$hr, pch = 19, col = "steelblue", cex = 2,
       xlab = "Total Sample Size", ylab = "Hazard Ratio",
       main = "PFS: Effect Size by Sample Size",
       ylim = c(0.2, 1.0))
  abline(h = 1, lty = 2, col = "gray")
  text(total_n, pfs_data$hr, labels = pfs_data$study, pos = 3, cex = 0.7)
})
dev.off()
cat("  Saved: bubble_PFS_samplesize.png\n")

# ============================================================================
# STEP 14: EXPORT SUMMARY RESULTS
# ============================================================================

cat("\n=============================================\n")
cat("  EXPORTING RESULTS\n")
cat("=============================================\n\n")

# ---- 14.1 Summary Table ----
results_summary <- data.frame(
  Outcome = c("PFS", "OS", "ORR", "DCR", "Grade >=3 TRAEs"),
  Studies = c(meta_pfs$k, meta_os$k, meta_orr$k, meta_dcr$k, meta_trae3$k),
  Effect_Measure = c("HR", "HR", "RR", "RR", "RR"),
  Pooled_Effect = c(
    round(exp(meta_pfs$TE.random), 3),
    round(exp(meta_os$TE.random), 3),
    round(exp(meta_orr$TE.random), 3),
    round(exp(meta_dcr$TE.random), 3),
    round(exp(meta_trae3$TE.random), 3)
  ),
  CI_Lower = c(
    round(exp(meta_pfs$lower.random), 3),
    round(exp(meta_os$lower.random), 3),
    round(exp(meta_orr$lower.random), 3),
    round(exp(meta_dcr$lower.random), 3),
    round(exp(meta_trae3$lower.random), 3)
  ),
  CI_Upper = c(
    round(exp(meta_pfs$upper.random), 3),
    round(exp(meta_os$upper.random), 3),
    round(exp(meta_orr$upper.random), 3),
    round(exp(meta_dcr$upper.random), 3),
    round(exp(meta_trae3$upper.random), 3)
  ),
  P_value = c(
    formatC(meta_pfs$pval.random, format = "e", digits = 2),
    formatC(meta_os$pval.random, format = "e", digits = 2),
    formatC(meta_orr$pval.random, format = "e", digits = 2),
    formatC(meta_dcr$pval.random, format = "e", digits = 2),
    formatC(meta_trae3$pval.random, format = "e", digits = 2)
  ),
  I2 = c(
    paste0(round(meta_pfs$I2 * 100, 1), "%"),
```

```
    paste0(round(meta_os$I2 * 100, 1), "%"),
    paste0(round(meta_orr$I2 * 100, 1), "%"),
    paste0(round(meta_dcr$I2 * 100, 1), "%"),
    paste0(round(meta_trae3$I2 * 100, 1), "%")
  ),
  Tau2 = c(
    round(meta_pfs$tau2, 4),
    round(meta_os$tau2, 4),
    round(meta_orr$tau2, 4),
    round(meta_dcr$tau2, 4),
    round(meta_trae3$tau2, 4)
  ),
  Heterogeneity_P = c(
    format(meta_pfs$pval.Q, digits = 3, nsmall = 3),
    format(meta_os$pval.Q, digits = 3, nsmall = 3),
    format(meta_orr$pval.Q, digits = 3, nsmall = 3),
    format(meta_dcr$pval.Q, digits = 3, nsmall = 3),
    format(meta_trae3$pval.Q, digits = 3, nsmall = 3)
  ),
  Model = c("Random (REML)", "Random (REML)", "Random (REML)",
            "Random (REML)", "Random (REML)")
)

print(results_summary)
write.csv(results_summary, file.path(output_dir, "meta_analysis_summary.csv"),
          row.names = FALSE, fileEncoding = "UTF-8")
cat("  Saved: meta_analysis_summary.csv\n")

# ---- 14.2 Individual Study Results ----
pfs_results <- data.frame(
  Study = pfs_data$study,
  Outcome = "PFS",
  HR = pfs_data$hr,
  CI_95 = paste0("(", pfs_data$lower, " - ", pfs_data$upper, ")"),
  P_value = pfs_data$p_value,
  Weight_Percent = round(meta_pfs$w.random / sum(meta_pfs$w.random, na.rm = TRUE) * 100, 1)
)

os_results <- data.frame(
  Study = os_data$study,
  Outcome = "OS",
  HR = os_data$hr,
  CI_95 = paste0("(", os_data$lower, " - ", os_data$upper, ")"),
  P_value = os_data$p_value,
  Weight_Percent = round(meta_os$w.random / sum(meta_os$w.random, na.rm = TRUE) * 100, 1)
)

all_results <- rbind(pfs_results, os_results)
write.csv(all_results, file.path(output_dir, "individual_study_results.csv"),
          row.names = FALSE, fileEncoding = "UTF-8")
cat("  Saved: individual_study_results.csv\n")

# ============================================================================
# STEP 15: GENERATE TEXT REPORT
# ============================================================================
```

```
cat("\n========================================\n")
cat("  GENERATING TEXT REPORT\n")
cat("========================================\n\n")

report_text <- paste0(
"================================================================================\n",
"  META-ANALYSIS REPORT\n",
"  ICI + Chemotherapy vs. Chemotherapy Alone in Advanced Biliary Tract Cancer\n",
"================================================================================\n\n",
"STUDIES INCLUDED: 6 retrospective studies\n",
"  1. Zhao 2021    (China, Single-center, n=90,  Mixed BTC)\n",
"  2. Lu 2024      (China, Single-center, n=186, Mixed BTC)\n",
"  3. Madzikatire 2024 (China, Single-center, n=81, iCCA)\n",
"  4. Rimini 2024  (Italy, Multi-center, n=563, Mixed BTC)\n",
"  5. Zhang 2025   (China, Single-center, n=150, GBC)\n",
"  6. Zhao 2025    (China, Single-center, n=183, Mixed BTC)\n\n",
"TOTAL PATIENTS: 1,253 (Experimental: 694, Control: 559)\n\n",
"================================================================================\n",
"  PRIMARY ENDPOINTS\n",
"================================================================================\n\n",
"1. PROGRESSION-FREE SURVIVAL (PFS)\n",
"   Pooled HR = ", round(exp(meta_pfs$TE.random), 3),
" (95% CI: ", round(exp(meta_pfs$lower.random), 3),
" - ", round(exp(meta_pfs$upper.random), 3), ")\n",
"   Z = ", round(meta_pfs$zval.random, 2), ", P-value = ",
formatC(meta_pfs$pval.random, format = "e", digits = 2), "\n",
"   Heterogeneity: I2 = ", round(meta_pfs$I2 * 100, 1), "%, tau2 = ",
round(meta_pfs$tau2, 4), ", Q-test P = ",
format(meta_pfs$pval.Q, digits = 3), "\n",
"   Interpretation: Statistically significant PFS benefit with ICI + chemotherapy\n\n",
"2. OVERALL SURVIVAL (OS)\n",
"   Pooled HR = ", round(exp(meta_os$TE.random), 3),
" (95% CI: ", round(exp(meta_os$lower.random), 3),
" - ", round(exp(meta_os$upper.random), 3), ")\n",
"   Z = ", round(meta_os$zval.random, 2), ", P-value = ",
formatC(meta_os$pval.random, format = "e", digits = 2), "\n",
"   Heterogeneity: I2 = ", round(meta_os$I2 * 100, 1), "%, tau2 = ",
round(meta_os$tau2, 4), ", Q-test P = ",
format(meta_os$pval.Q, digits = 3), "\n",
"   Interpretation: Statistically significant OS benefit with ICI + chemotherapy\n\n",
"================================================================================\n",
"  SECONDARY ENDPOINTS\n",
"================================================================================\n\n",
"3. OBJECTIVE RESPONSE RATE (ORR)\n",
"   Pooled RR = ", round(exp(meta_orr$TE.random), 3),
" (95% CI: ", round(exp(meta_orr$lower.random), 3),
" - ", round(exp(meta_orr$upper.random), 3), ")\n",
"   P-value = ", formatC(meta_orr$pval.random, format = "e", digits = 2), "\n",
"   Heterogeneity: I2 = ", round(meta_orr$I2 * 100, 1), "%\n",
"   Interpretation: Significantly higher ORR with ICI + chemotherapy\n\n",
"4. DISEASE CONTROL RATE (DCR)\n",
"   Pooled RR = ", round(exp(meta_dcr$TE.random), 3),
" (95% CI: ", round(exp(meta_dcr$lower.random), 3),
" - ", round(exp(meta_dcr$upper.random), 3), ")\n",
```

```
"   P-value = ", formatC(meta_dcr$pval.random, format = "e", digits = 2), "\n",
"   Heterogeneity: I2 = ", round(meta_dcr$I2 * 100, 1), "%\n",
"   Interpretation: Significantly higher DCR with ICI + chemotherapy\n\n",
"===============================================================================\n",
"  SAFETY\n",
"===============================================================================\n\n",
"5. GRADE >=3 TREATMENT-RELATED ADVERSE EVENTS\n",
"   Pooled RR = ", round(exp(meta_trae3$TE.random), 3),
" (95% CI: ", round(exp(meta_trae3$lower.random), 3),
" - ", round(exp(meta_trae3$upper.random), 3), ")\n",
"   P-value = ", formatC(meta_trae3$pval.random, format = "e", digits = 2), "\n",
"   Heterogeneity: I2 = ", round(meta_trae3$I2 * 100, 1), "%, tau2 = ",
round(meta_trae3$tau2, 4), "\n",
"   Interpretation: No statistically significant difference in Grade >=3 TRAEs\n\n",
"===============================================================================\n",
"  SUBGROUP ANALYSIS\n",
"===============================================================================\n\n",
"PFS by Tumor Type:\n",
"  - Mixed BTC (4 studies): HR = 0.55 (95% CI: 0.43-0.71)\n",
"  - iCCA      (1 study):  HR = 0.48 (95% CI: 0.27-0.86)\n",
"  - GBC       (1 study):  HR = 0.39 (95% CI: 0.28-0.56)\n",
"  Subgroup interaction test: P = 0.22 (not significant)\n\n",
"OS by Tumor Type:\n",
"  - Mixed BTC (4 studies): HR = 0.65 (95% CI: 0.34-1.26)\n",
"  - iCCA      (1 study):  HR = 0.53 (95% CI: 0.30-0.94)\n",
"  - GBC       (1 study):  HR = 0.53 (95% CI: 0.36-0.79)\n",
"  Subgroup interaction test: P = 0.73 (not significant)\n\n",
"===============================================================================\n",
"  CONCLUSION\n",
"===============================================================================\n\n",
"Immune checkpoint inhibitors combined with gemcitabine-based chemotherapy\n",
"significantly improved PFS (HR=0.52, 95%CI:0.43-0.63), OS (HR=0.61, 95%CI:\n",
"0.42-0.87), ORR (RR=1.97, 95%CI:1.43-2.71), and DCR (RR=1.26, 95%CI:1.09-1.45)\n",
"compared with chemotherapy alone in patients with advanced biliary tract cancer.\n",
"No significant increase in Grade >=3 TRAEs was observed (RR=1.61, 95%CI:0.83-3.10).\n",
"The treatment benefit was consistent across different tumor types (iCCA, GBC,\n",
"and mixed BTC), geographic regions, and study designs.\n\n",
"Output directory: ", output_dir, "\n",
"Generated: ", format(Sys.time(), "%Y-%m-%d %H:%M:%S"), "\n",
"==============================================================================="
)

cat(report_text)
writeLines(report_text, file.path(output_dir, "meta_analysis_report.txt"))
cat("\n  Saved: meta_analysis_report.txt\n")

# ===========================================================================
# STEP 16: PRINT FILE LIST
# ===========================================================================

cat("\n=======================================\n")
cat("  ALL FILES GENERATED\n")
cat("=======================================\n\n")
cat("Directory: ", normalizePath(output_dir), "\n\n")
```

```
generated_files <- list.files(output_dir, pattern = "\\.(png|csv|txt)$")
for (f in sort(generated_files)) {
  cat("  [OK] ", f, "\n")
}

cat("\n===== META-ANALYSIS COMPLETE =====\n")
```

Supplementary Code 3: Revised Figures and Supplementary Plots

File: Supplementary_Code_3_Revised_Figures_and_Supplementary_Plots.R

Purpose: Figures revised in response to reviewer comments and newly added supplementary plots

Corresponds to: Revised Figure 4, Figure 15, Supplementary Figures S1-S2

```
# ==============================================================================
# Supplementary Code for:
#   PD-1/PD-L1 Inhibitors Combined with Chemotherapy as First-Line
#   Treatment for Advanced Biliary Tract Cancer:
#   A Systematic Review and Meta-Analysis
#
# Journal: Frontiers in Medicine
#
# File: Supplementary_Code_3_Revised_Figures_and_Supplementary_Plots.R
#
# Description:
#   Regenerated figures and supplementary plots created in response to reviewer comments.
#
# Corresponds to: Figure 4 (revised), Figure 15a/15b, Supplementary Figure S1, Supplementary Figure S2
#
# R version: 4.6.1
# Required packages:
#   - meta     (v8.5-0)
#   - metafor  (v5.0-1)
#   - ggplot2
#   - gridExtra
#   - grid
#
# Notes:
#   - All study data are embedded directly in the script for full reproducibility
#   - Figures are saved as both PNG (300 dpi) and PDF formats
#   - Run the script from top to bottom to reproduce all analyses
#
# ==============================================================================

set.seed(20240801)

install_if_missing <- function(pkg) {
  if (!requireNamespace(pkg, quietly = TRUE)) {
    install.packages(pkg, repos = "https://cran.r-project.org")
  }
}
packages <- c("meta", "metafor", "ggplot2", "grid", "gridExtra")
invisible(sapply(packages, install_if_missing))

library(meta)
library(metafor)
library(ggplot2)
library(grid)
```

```
library(gridExtra)

out_dir <- "./reviewer_revised_figures"
dir.create(out_dir, showWarnings = FALSE, recursive = TRUE)

calc_se_loghr <- function(lower, upper) {
  (log(upper) - log(lower)) / (2 * 1.96)
}

cat("========================================\n")
cat("  Reviewer-revised figures and supplementary plots\n")
cat("========================================\n\n")


# ============================================================================
# FIGURE 4: RCT PFS forest plot (fixed-effect model, revised)
# Reviewer 1 noted: PFS I2 = 29.8% (< 50%), fixed-effect model should be used per protocol
# ============================================================================
cat("--- Figure 4: RCT PFS forest plot (Fixed-effect model) ---\n")

rct_pfs_studies <- c("Oh 2022/2024 TOPAZ-1", "Kelley 2023 KEYNOTE-966")
rct_pfs_hr  <- c(0.75, 0.86)
rct_pfs_low <- c(0.63, 0.75)
rct_pfs_up  <- c(0.89, 1.00)
rct_pfs_loghr <- log(rct_pfs_hr)
rct_pfs_se    <- mapply(calc_se_loghr, rct_pfs_low, rct_pfs_up)

meta_rct_pfs_fe <- metagen(
  TE = rct_pfs_loghr, seTE = rct_pfs_se,
  studlab = rct_pfs_studies,
  sm = "HR",
  method.tau = "DL",
  method.random.ci = "classic",
  common = TRUE,
  random = FALSE,
  title = "RCT: Progression-Free Survival (Fixed-effect)"
)
print(summary(meta_rct_pfs_fe))

png(file.path(out_dir, "Fig4_RCT_PFS_forest_fixed_effect.png"),
    width = 13*300, height = 5*300, res = 300)
forest(meta_rct_pfs_fe,
       layout = "JAMA",
       fontsize = 11,
       leftcols = c("studlab", "effect", "ci", "w.common"),
       leftlabs = c("Study", "HR", "95% CI", "Weight\n(Fixed)"),
       rightcols = c("effect", "ci"),
       rightlabs = c("HR", "95% CI"),
       col.diamond = "steelblue",
       col.square = "steelblue",
       print.I2 = TRUE,
       print.tau2 = TRUE,
       print.pval.Q = TRUE,
       xlim = c(0.4, 1.4),
       at = c(0.4, 0.6, 0.8, 1.0, 1.2, 1.4),
```

```
       ref = 1,
       label.left = "Favors ICI + Chemo",
       label.right = "Favors Chemo",
       main = "RCT: Progression-Free Survival (PFS)")
dev.off()

pdf(file.path(out_dir, "Fig4_RCT_PFS_forest_fixed_effect.pdf"), width = 13, height = 5)
forest(meta_rct_pfs_fe,
       layout = "JAMA",
       fontsize = 11,
       leftcols = c("studlab", "effect", "ci", "w.common"),
       leftlabs = c("Study", "HR", "95% CI", "Weight\n(Fixed)"),
       rightcols = c("effect", "ci"),
       rightlabs = c("HR", "95% CI"),
       col.diamond = "steelblue",
       col.square = "steelblue",
       print.I2 = TRUE,
       print.tau2 = TRUE,
       print.pval.Q = TRUE,
       xlim = c(0.4, 1.4),
       at = c(0.4, 0.6, 0.8, 1.0, 1.2, 1.4),
       ref = 1,
       label.left = "Favors ICI + Chemo",
       label.right = "Favors Chemo",
       main = "RCT: Progression-Free Survival (PFS)")
dev.off()
cat("  Saved: Fig4_RCT_PFS_forest_fixed_effect.png & .pdf\n")


# ============================================================================
# FIGURE 15a: Retrospective PFS subgroup forest plot (by tumor type)
# ============================================================================
cat("\n--- Figure 15a: Retrospective PFS subgroup forest plot (by tumor type) ---\n")

ps_pfs_studies <- c("Zhao 2021", "Lu 2024", "Madzikatire 2024",
                    "Rimini 2024", "Zhang 2025", "Zhao 2025")
ps_pfs_hr  <- c(0.62, 0.65, 0.48, 0.57, 0.393, 0.46)
ps_pfs_low <- c(0.39, 0.48, 0.27, 0.47, 0.278, 0.38)
ps_pfs_up  <- c(0.94, 0.88, 0.87, 0.70, 0.555, 0.54)
ps_pfs_loghr <- log(ps_pfs_hr)
ps_pfs_se    <- mapply(calc_se_loghr, ps_pfs_low, ps_pfs_up)

ps_pfs_tumor <- c("Mixed BTC", "Mixed BTC", "iCCA",
                  "Mixed BTC", "GBC", "Mixed BTC")

meta_ps_pfs_sub <- metagen(
  TE = ps_pfs_loghr, seTE = ps_pfs_se,
  studlab = ps_pfs_studies,
  sm = "HR",
  method.tau = "REML",
  method.random.ci = "HK",
  common = TRUE, random = TRUE,
  subgroup = ps_pfs_tumor,
  print.subgroup.name = TRUE,
  title = "Retrospective: PFS by Tumor Type"
```

```
)
print(summary(meta_ps_pfs_sub))

rma_pfs_sub <- rma.uni(yi = ps_pfs_loghr, sei = ps_pfs_se,
                       mods = ~ as.factor(ps_pfs_tumor), method = "REML")
interaction_p_pfs <- rma_pfs_sub$QMp
cat(sprintf("  Interaction test p-value (tumor type, PFS): %.4f\n", interaction_p_pfs))

png(file.path(out_dir, "Fig15a_PS_PFS_subgroup_tumor.png"),
    width = 13*300, height = 9*300, res = 300)
forest(meta_ps_pfs_sub,
       layout = "JAMA",
       fontsize = 10,
       leftcols = c("studlab", "effect", "ci", "w.random"),
       leftlabs = c("Study", "HR", "95% CI", "Weight"),
       rightcols = c("effect", "ci"),
       rightlabs = c("HR", "95% CI"),
       col.diamond.random = "darkblue",
       col.bg = "steelblue",
       print.I2 = TRUE,
       print.tau2 = TRUE,
       print.pval.Q = TRUE,
       print.pval.subgroup = TRUE,
       xlim = c(0.1, 1.5),
       at = c(0.25, 0.5, 0.75, 1.0, 1.5),
       ref = 1,
       label.left = "Favors ICI + Chemo",
       label.right = "Favors Chemo",
       main = "Retrospective Studies: PFS Subgroup by Tumor Type",
       subgroup.name = "Tumor Type")
dev.off()

pdf(file.path(out_dir, "Fig15a_PS_PFS_subgroup_tumor.pdf"), width = 13, height = 9)
forest(meta_ps_pfs_sub,
       layout = "JAMA",
       fontsize = 10,
       leftcols = c("studlab", "effect", "ci", "w.random"),
       leftlabs = c("Study", "HR", "95% CI", "Weight"),
       rightcols = c("effect", "ci"),
       rightlabs = c("HR", "95% CI"),
       col.diamond.random = "darkblue",
       col.bg = "steelblue",
       print.I2 = TRUE,
       print.tau2 = TRUE,
       print.pval.Q = TRUE,
       print.pval.subgroup = TRUE,
       xlim = c(0.1, 1.5),
       at = c(0.25, 0.5, 0.75, 1.0, 1.5),
       ref = 1,
       label.left = "Favors ICI + Chemo",
       label.right = "Favors Chemo",
       main = "Retrospective Studies: PFS Subgroup by Tumor Type",
       subgroup.name = "Tumor Type")
dev.off()
cat("  Saved: Fig15a_PS_PFS_subgroup_tumor.png & .pdf\n")
```

```
# ============================================================================
# FIGURE 15b: Retrospective OS subgroup forest plot (by tumor type)
# Reviewer 1 noted: OS subgroup results lacked visual representation; added here
# ============================================================================
cat("\n--- Figure 15b: Retrospective OS subgroup forest plot (by tumor type) ---\n")

ps_os_studies <- c("Zhao 2021", "Lu 2024", "Madzikatire 2024",
                   "Rimini 2024", "Zhang 2025", "Zhao 2025")
ps_os_hr  <- c(0.85, 0.91, 0.53, 0.63, 0.53, 0.39)
ps_os_low <- c(0.52, 0.62, 0.30, 0.50, 0.357, 0.32)
ps_os_up  <- c(1.37, 1.32, 0.94, 0.80, 0.786, 0.47)
ps_os_loghr <- log(ps_os_hr)
ps_os_se    <- mapply(calc_se_loghr, ps_os_low, ps_os_up)

ps_os_tumor <- c("Mixed BTC", "Mixed BTC", "iCCA",
                 "Mixed BTC", "GBC", "Mixed BTC")

meta_ps_os_sub <- metagen(
  TE = ps_os_loghr, seTE = ps_os_se,
  studlab = ps_os_studies,
  sm = "HR",
  method.tau = "REML",
  method.random.ci = "HK",
  common = TRUE, random = TRUE,
  subgroup = ps_os_tumor,
  print.subgroup.name = TRUE,
  title = "Retrospective: OS by Tumor Type"
)
print(summary(meta_ps_os_sub))

rma_os_sub <- rma.uni(yi = ps_os_loghr, sei = ps_os_se,
                      mods = ~ as.factor(ps_os_tumor), method = "REML")
interaction_p_os <- rma_os_sub$QMp
cat(sprintf("  Interaction test p-value (tumor type, OS): %.4f\n", interaction_p_os))

png(file.path(out_dir, "Fig15b_PS_OS_subgroup_tumor.png"),
    width = 13*300, height = 9*300, res = 300)
forest(meta_ps_os_sub,
       layout = "JAMA",
       fontsize = 10,
       leftcols = c("studlab", "effect", "ci", "w.random"),
       leftlabs = c("Study", "HR", "95% CI", "Weight"),
       rightcols = c("effect", "ci"),
       rightlabs = c("HR", "95% CI"),
       col.diamond.random = "darkred",
       col.bg = "indianred",
       print.I2 = TRUE,
       print.tau2 = TRUE,
       print.pval.Q = TRUE,
       print.pval.subgroup = TRUE,
       xlim = c(0.1, 2.0),
       at = c(0.25, 0.5, 0.75, 1.0, 1.5, 2.0),
       ref = 1,
```

```
       label.left = "Favors ICI + Chemo",
       label.right = "Favors Chemo",
       main = "Retrospective Studies: OS Subgroup by Tumor Type",
       subgroup.name = "Tumor Type")
dev.off()

pdf(file.path(out_dir, "Fig15b_PS_OS_subgroup_tumor.pdf"), width = 13, height = 9)
forest(meta_ps_os_sub,
       layout = "JAMA",
       fontsize = 10,
       leftcols = c("studlab", "effect", "ci", "w.random"),
       leftlabs = c("Study", "HR", "95% CI", "Weight"),
       rightcols = c("effect", "ci"),
       rightlabs = c("HR", "95% CI"),
       col.diamond.random = "darkred",
       col.bg = "indianred",
       print.I2 = TRUE,
       print.tau2 = TRUE,
       print.pval.Q = TRUE,
       print.pval.subgroup = TRUE,
       xlim = c(0.1, 2.0),
       at = c(0.25, 0.5, 0.75, 1.0, 1.5, 2.0),
       ref = 1,
       label.left = "Favors ICI + Chemo",
       label.right = "Favors Chemo",
       main = "Retrospective Studies: OS Subgroup by Tumor Type",
       subgroup.name = "Tumor Type")
dev.off()
cat("  Saved: Fig15b_PS_OS_subgroup_tumor.png & .pdf\n")


# ============================================================================
# FIGURE S1: Cohort overlap sensitivity analysis (4 scenarios)
# Reviewer 1 requested: assess impact of excluding potentially overlapping/related studies
# Zhao 2021 and Zhao 2025 are from the same center in China; potential patient overlap risk
# ============================================================================
cat("\n--- Figure S1: Cohort overlap sensitivity analysis ---\n")

ps_pfs_all_hr  <- c(0.62, 0.65, 0.48, 0.57, 0.393, 0.46)
ps_pfs_all_low <- c(0.39, 0.48, 0.27, 0.47, 0.278, 0.38)
ps_pfs_all_up  <- c(0.94, 0.88, 0.87, 0.70, 0.555, 0.54)

ps_os_all_hr  <- c(0.85, 0.91, 0.53, 0.63, 0.53, 0.39)
ps_os_all_low <- c(0.52, 0.62, 0.30, 0.50, 0.357, 0.32)
ps_os_all_up  <- c(1.37, 1.32, 0.94, 0.80, 0.786, 0.47)

calc_pooled <- function(indices, hr_vec, low_vec, up_vec) {
  loghr <- log(hr_vec[indices])
  se <- mapply(calc_se_loghr, low_vec[indices], up_vec[indices])
  m <- metagen(TE = loghr, seTE = se, sm = "HR",
               method.tau = "REML", method.random.ci = "HK",
               common = FALSE, random = TRUE)
  c(hr = exp(m$TE.random), low = exp(m$lower.random), up = exp(m$upper.random),
    i2 = m$I2, pval_q = m$pval.Q)
}
```

```
idx_all      <- 1:6
idx_excl_z1  <- c(2, 3, 4, 5, 6)
idx_excl_z5  <- c(1, 2, 3, 4, 5)
idx_excl_both<- c(2, 3, 4, 5)

scenario_labels <- c("All 6 studies",
                     "Excluding Zhao 2021",
                     "Excluding Zhao 2025",
                     "Excluding Zhao 2021 & 2025")

pfs_res <- rbind(
  calc_pooled(idx_all, ps_pfs_all_hr, ps_pfs_all_low, ps_pfs_all_up),
  calc_pooled(idx_excl_z1, ps_pfs_all_hr, ps_pfs_all_low, ps_pfs_all_up),
  calc_pooled(idx_excl_z5, ps_pfs_all_hr, ps_pfs_all_low, ps_pfs_all_up),
  calc_pooled(idx_excl_both, ps_pfs_all_hr, ps_pfs_all_low, ps_pfs_all_up)
)
rownames(pfs_res) <- scenario_labels
cat("  PFS scenarios:\n"); print(round(pfs_res, 3))

os_res <- rbind(
  calc_pooled(idx_all, ps_os_all_hr, ps_os_all_low, ps_os_all_up),
  calc_pooled(idx_excl_z1, ps_os_all_hr, ps_os_all_low, ps_os_all_up),
  calc_pooled(idx_excl_z5, ps_os_all_hr, ps_os_all_low, ps_os_all_up),
  calc_pooled(idx_excl_both, ps_os_all_hr, ps_os_all_low, ps_os_all_up)
)
rownames(os_res) <- scenario_labels
cat("  OS scenarios:\n"); print(round(os_res, 3))

sens_labels <- c(paste0("PFS: ", scenario_labels), paste0("OS: ", scenario_labels))
sens_hr  <- c(pfs_res[, "hr"],  os_res[, "hr"])
sens_low <- c(pfs_res[, "low"], os_res[, "low"])
sens_up  <- c(pfs_res[, "up"],  os_res[, "up"])
sens_se  <- (log(sens_up) - log(sens_low)) / (2 * 1.96)
sens_loghr <- log(sens_hr)

sens_col <- c(rep("steelblue", 4), rep("indianred", 4))

png(file.path(out_dir, "FigS1_Overlap_Sensitivity_Analysis.png"),
    width = 12*300, height = 7*300, res = 300)
par(mar = c(5, 14, 4, 2) + 0.1)
forest(sens_loghr, sens_se,
       slab = sens_labels,
       xlab = "Hazard Ratio",
       ilab.xpos = -2.5,
       at = c(0.3, 0.4, 0.5, 0.6, 0.8, 1.0),
       transf = exp,
       refline = 1,
       pch = 15,
       psize = 1.5,
       col = sens_col,
       cex.lab = 1.1, cex.axis = 1.0,
       main = "Sensitivity Analysis: Impact of Potential Cohort Overlap")
abline(h = 4.5, lty = "dashed", col = "gray70")
text(-2.8, 8.2, "PFS", font = 2, cex = 1.2, col = "steelblue")
```

```
text(-2.8, 3.2, "OS",  font = 2, cex = 1.2, col = "indianred")
mtext("Zhao 2021 and Zhao 2025 from the same center in China; removing either or both assesses robustness to cohort ov
      side = 1, line = 3.5, cex = 0.85, adj = 0)
dev.off()

pdf(file.path(out_dir, "FigS1_Overlap_Sensitivity_Analysis.pdf"), width = 12, height = 7)
par(mar = c(5, 14, 4, 2) + 0.1)
forest(sens_loghr, sens_se,
       slab = sens_labels,
       xlab = "Hazard Ratio",
       ilab.xpos = -2.5,
       at = c(0.3, 0.4, 0.5, 0.6, 0.8, 1.0),
       transf = exp,
       refline = 1,
       pch = 15,
       psize = 1.5,
       col = sens_col,
       cex.lab = 1.1, cex.axis = 1.0,
       main = "Sensitivity Analysis: Impact of Potential Cohort Overlap")
abline(h = 4.5, lty = "dashed", col = "gray70")
text(-2.8, 8.2, "PFS", font = 2, cex = 1.2, col = "steelblue")
text(-2.8, 3.2, "OS",  font = 2, cex = 1.2, col = "indianred")
mtext("Zhao 2021 and Zhao 2025 from the same center in China; removing either or both assesses robustness to cohort ov
      side = 1, line = 3.5, cex = 0.85, adj = 0)
dev.off()
cat("  Saved: FigS1_Overlap_Sensitivity_Analysis.png & .pdf\n")


# ==============================================================================
# FIGURE S2: Retrospective ICI-type subgroup forest plots (PFS + OS)
# Reviewer 3 questioned: are all PD-1/PD-L1 inhibitors interchangeable?
# ==============================================================================
cat("\n--- Figure S2: ICI-type subgroup forest plots (PFS + OS) ---\n")

ps_ici_type <- c("PD-1 (various)", "PD-L1 (Durvalumab)", "Mixed (PD-1/PD-L1)",
                 "PD-L1 (Durvalumab)", "PD-1 (Tislelizumab)", "PD-1 (Camrelizumab)")

meta_ps_pfs_ici <- metagen(
  TE = ps_pfs_loghr, seTE = ps_pfs_se,
  studlab = ps_pfs_studies,
  sm = "HR",
  method.tau = "REML",
  method.random.ci = "HK",
  common = TRUE, random = TRUE,
  subgroup = ps_ici_type,
  print.subgroup.name = TRUE,
  title = "Retrospective: PFS by ICI Type"
)
print(summary(meta_ps_pfs_ici))

rma_pfs_ici <- rma.uni(yi = ps_pfs_loghr, sei = ps_pfs_se,
                       mods = ~ as.factor(ps_ici_type), method = "REML")
interaction_p_pfs_ici <- rma_pfs_ici$QMp
cat(sprintf("  Interaction test p-value (ICI type, PFS): %.4f\n", interaction_p_pfs_ici))
```

```
png(file.path(out_dir, "FigS2a_PS_PFS_subgroup_ICI_type.png"),
    width = 13*300, height = 9*300, res = 300)
forest(meta_ps_pfs_ici,
       layout = "JAMA",
       fontsize = 10,
       leftcols = c("studlab", "effect", "ci", "w.random"),
       leftlabs = c("Study", "HR", "95% CI", "Weight"),
       rightcols = c("effect", "ci"),
       rightlabs = c("HR", "95% CI"),
       col.diamond.random = "darkgreen",
       col.bg = "seagreen",
       print.I2 = TRUE,
       print.tau2 = TRUE,
       print.pval.Q = TRUE,
       print.pval.subgroup = TRUE,
       xlim = c(0.1, 1.5),
       at = c(0.25, 0.5, 0.75, 1.0, 1.5),
       ref = 1,
       label.left = "Favors ICI + Chemo",
       label.right = "Favors Chemo",
       main = "Retrospective Studies: PFS Subgroup by ICI Type",
       subgroup.name = "ICI Type")
dev.off()

pdf(file.path(out_dir, "FigS2a_PS_PFS_subgroup_ICI_type.pdf"), width = 13, height = 9)
forest(meta_ps_pfs_ici,
       layout = "JAMA",
       fontsize = 10,
       leftcols = c("studlab", "effect", "ci", "w.random"),
       leftlabs = c("Study", "HR", "95% CI", "Weight"),
       rightcols = c("effect", "ci"),
       rightlabs = c("HR", "95% CI"),
       col.diamond.random = "darkgreen",
       col.bg = "seagreen",
       print.I2 = TRUE,
       print.tau2 = TRUE,
       print.pval.Q = TRUE,
       print.pval.subgroup = TRUE,
       xlim = c(0.1, 1.5),
       at = c(0.25, 0.5, 0.75, 1.0, 1.5),
       ref = 1,
       label.left = "Favors ICI + Chemo",
       label.right = "Favors Chemo",
       main = "Retrospective Studies: PFS Subgroup by ICI Type",
       subgroup.name = "ICI Type")
dev.off()
cat("  Saved: FigS2a_PS_PFS_subgroup_ICI_type.png & .pdf\n")

meta_ps_os_ici <- metagen(
  TE = ps_os_loghr, seTE = ps_os_se,
  studlab = ps_os_studies,
  sm = "HR",
  method.tau = "REML",
  method.random.ci = "HK",
  common = TRUE, random = TRUE,
```

```
  subgroup = ps_ici_type,
  print.subgroup.name = TRUE,
  title = "Retrospective: OS by ICI Type"
)
print(summary(meta_ps_os_ici))

rma_os_ici <- rma.uni(yi = ps_os_loghr, sei = ps_os_se,
                      mods = ~ as.factor(ps_ici_type), method = "REML")
interaction_p_os_ici <- rma_os_ici$QMp
cat(sprintf("  Interaction test p-value (ICI type, OS): %.4f\n", interaction_p_os_ici))

png(file.path(out_dir, "FigS2b_PS_OS_subgroup_ICI_type.png"),
    width = 13*300, height = 9*300, res = 300)
forest(meta_ps_os_ici,
       layout = "JAMA",
       fontsize = 10,
       leftcols = c("studlab", "effect", "ci", "w.random"),
       leftlabs = c("Study", "HR", "95% CI", "Weight"),
       rightcols = c("effect", "ci"),
       rightlabs = c("HR", "95% CI"),
       col.diamond.random = "darkorange",
       col.bg = "darkorange3",
       print.I2 = TRUE,
       print.tau2 = TRUE,
       print.pval.Q = TRUE,
       print.pval.subgroup = TRUE,
       xlim = c(0.1, 2.0),
       at = c(0.25, 0.5, 0.75, 1.0, 1.5, 2.0),
       ref = 1,
       label.left = "Favors ICI + Chemo",
       label.right = "Favors Chemo",
       main = "Retrospective Studies: OS Subgroup by ICI Type",
       subgroup.name = "ICI Type")
dev.off()

pdf(file.path(out_dir, "FigS2b_PS_OS_subgroup_ICI_type.pdf"), width = 13, height = 9)
forest(meta_ps_os_ici,
       layout = "JAMA",
       fontsize = 10,
       leftcols = c("studlab", "effect", "ci", "w.random"),
       leftlabs = c("Study", "HR", "95% CI", "Weight"),
       rightcols = c("effect", "ci"),
       rightlabs = c("HR", "95% CI"),
       col.diamond.random = "darkorange",
       col.bg = "darkorange3",
       print.I2 = TRUE,
       print.tau2 = TRUE,
       print.pval.Q = TRUE,
       print.pval.subgroup = TRUE,
       xlim = c(0.1, 2.0),
       at = c(0.25, 0.5, 0.75, 1.0, 1.5, 2.0),
       ref = 1,
       label.left = "Favors ICI + Chemo",
       label.right = "Favors Chemo",
       main = "Retrospective Studies: OS Subgroup by ICI Type",
```

```
       subgroup.name = "ICI Type")
dev.off()
cat("  Saved: FigS2b_PS_OS_subgroup_ICI_type.png & .pdf\n")


# ============================================================================
# Complete
# ============================================================================
cat("\n=========================================\n")
cat("  All reviewer-revised figures and supplementary plots generated\n")
cat("=========================================\n\n")
cat("List of generated figures:\n")
cat("  Fig 4  : RCT PFS forest plot (Fixed-effect model)\n")
cat("  Fig 15a: Retrospective PFS subgroup forest plot (by tumor type)\n")
cat("  Fig 15b: Retrospective OS subgroup forest plot (by tumor type, added)\n")
cat("  Fig S1 : Cohort overlap sensitivity analysis (PFS + OS)\n")
cat("  Fig S2a: Retrospective PFS subgroup forest plot (by ICI type)\n")
cat("  Fig S2b: Retrospective OS subgroup forest plot (by ICI type)\n")
cat("\nAll figures saved to:", normalizePath(out_dir), "\n")
cat("Format: PNG (300dpi) + PDF\n")
```
