# Supplementary material for: PD-1/PD-L1 inhibitors combined with chemotherapy as first-line treatment for advanced biliary tract cancer: a systematic review and meta-analysis
Source: Front Med (Lausanne). 2026 Sep 3;13:1916331. doi: 10.3389/fmed.2026.1916331 (PMC13581943; doi:10.3389/fmed.2026.1916331)
Supplement: Supplementary file 2 [file Data_Sheet_1.DOCX]

Supplementary Material

# 1 Supplementary Data

Table 1 **Characteristics of included retrospective studies(Extended Version)**

| **Study** | **First author** | **Publication year** | **Country** | **Study design** | **Study period** | **Tumor type** | **Experimental group (n)** | **Control group (n)** | **Total sample size** | **Chemotherapy regimen (experimental)** | **Chemotherapy regimen (control)** | **ICI type** | **Primary outcome(s)** | **Secondary outcome(s)** |
| --- | --- | --- | --- | --- | --- | --- | --- | --- | --- | --- | --- | --- | --- | --- |
| TOPAZ-1 | Oh D-Y | 2022 | Multinational (17 countries) | Multicenter, double-blind, placebo-controlled RCT | NR | BTC (biliary tract cancer) | Durvalumab + GP (n=341) | Placebo + GP (n=344) | 685 | GP (gemcitabine 1000 mg/m2 d1,8 + cisplatin 25 mg/m2 d1,8) | GP (gemcitabine 1000 mg/m2 d1,8 + cisplatin 25 mg/m2 d1,8) | Durvalumab (anti-PD-L1) | OS | PFS, ORR, DCR, DOR, safety, PRO |
| KEYNOTE-966 | Kelley RK | 2023 | Multinational (Global) | Multicenter, double-blind, placebo-controlled RCT | NR | BTC (biliary tract cancer) | Pembrolizumab + GP (n=533) | Placebo + GP (n=536) | 1069 | GP (gemcitabine 1000 mg/m2 d1,8 + cisplatin 25 mg/m2 d1,8) | GP (gemcitabine 1000 mg/m2 d1,8 + cisplatin 25 mg/m2 d1,8) | Pembrolizumab (anti-PD-1) | OS | PFS, ORR, DOR, safety, QoL |
| Cui 2023 | Cui D | 2023 | China | Single-center, open-label RCT | NR | BTC (biliary tract cancer) | Nivolumab + GEMOX (n=50) | GEMOX (n=50) | 100 | GEMOX (gemcitabine 1000 mg/m2 + oxaliplatin 100 mg/m2) | GEMOX (gemcitabine 1000 mg/m2 + oxaliplatin 100 mg/m2) | Nivolumab (anti-PD-1) | ORR, DCR | Tumor markers (CEA, CA125, CA19-9, CA242, CA50), safety |
| Deng 2023 | Deng J | 2023 | China | Single-center, open-label RCT | NR | iCCA (intrahepatic cholangiocarcinoma) | Pembrolizumab + XELOX (n=40) | XELOX (n=40) | 80 | XELOX (capecitabine 1000 mg/m2 + oxaliplatin 100 mg/m2) | XELOX (capecitabine 1000 mg/m2 + oxaliplatin 100 mg/m2) | Pembrolizumab (anti-PD-1) | ORR | Safety, apoptotic factors (XIAP, Smac), tumor markers (CA19-9, CA125, CEA) |
| Li 2023 | Li R | 2023 | China | Single-center, open-label RCT | NR | BTC (biliary tract cancer) | Sintilimab + GC (n=33) | GC (n=33) | 66 | GC (gemcitabine 1000 mg/m2 + cisplatin 75 mg/m2) | GC (gemcitabine 1000 mg/m2 + cisplatin 75 mg/m2) | Sintilimab (anti-PD-1) | ORR | PFS, MST, tumor markers (CA19-9, CEA, CA125), immune function (CD3+, CD4+, CD8+), safety |

| **Study** | **First author** | **Publication year** | **Country** | **Study design** | **Study period** | **Tumor type** | **Experimental group (n)** | **Control group (n)** | **Total sample size** | **Chemotherapy regimen (experimental)** | **Chemotherapy regimen (control)** | **ICI type** | **Primary outcome(s)** | **Secondary outcome(s)** |
| --- | --- | --- | --- | --- | --- | --- | --- | --- | --- | --- | --- | --- | --- | --- |
| Zhao 2021 | Zhao S | 2021 | China | Single-center retrospective study | 2017.1-2020.3 | Mixed BTC | ICI + chemotherapy (n=45) | Chemotherapy (n=45) | 90 | Gemcitabine-based platinum-containing | Gemcitabine-based platinum-containing | Anti-PD-1 (nivolumab, sintilimab, pembrolizumab, toripalimab, or camrelizumab) | Not predefined | OS, PFS, ORR, DCR, safety |
| Lu 2024 | Lu Y | 2024 | China | Single-center retrospective study | 2020.1-2022.12 | Mixed BTC | Durvalumab + chemotherapy (n=93) | Chemotherapy (n=93) | 186 | Gemcitabine-based platinum-containing | Gemcitabine-based platinum-containing | Durvalumab (anti-PD-L1) | Not predefined | OS, PFS, ORR, DCR, safety |
| Madzikatire 2024 | Madzikatire TB | 2024 | China | Single-center retrospective study | 2019.12-2023.4 | iCCA | Anti-PD-1/PD-L1 + chemotherapy (n=51) | Chemotherapy (n=30) | 81 | GemCis, GEMOX, GS, or FOLFIRINOX | GemCis, GEMOX, GS, or FOLFIRINOX | Anti-PD-1/PD-L1 (durvalumab, pembrolizumab, or camrelizumab) | Not predefined | OS, PFS, ORR, DCR, safety |
| Rimini 2024 | Rimini M | 2024 | Italy | Multicenter retrospective study | 2006.3-2023.12 | Mixed BTC | Durvalumab + GP (n=350) | GP (n=213) | 563 | GP (cisplatin + gemcitabine) | GP (cisplatin + gemcitabine) | Durvalumab (anti-PD-L1) | Not predefined | OS, PFS, safety |
| Zhang 2025 | Zhang D | 2025 | China | Single-center retrospective study | 2020.1-2023.12 | GBC | Tislelizumab + GEMOX (n=62) | GEMOX (n=88) | 150 | GEMOX | GEMOX | Tislelizumab (anti-PD-1) | Not predefined | OS, PFS, ORR, DCR, safety |
| Zhao 2025 | Zhao J | 2025 | China | Single-center retrospective study | 2018.9-2021.9 | Mixed BTC | Camrelizumab + chemotherapy (n=93) | Chemotherapy (n=90) | 183 | GP or GEMOX | GP or GEMOX | Camrelizumab (anti-PD-1) | Not predefined | OS, PFS, ORR, DCR, safety |

# 2 Supplementary Figures and Tables

## 2.1 Supplementary Figures

**Figures 1 RoB2 risk of Bias Judgments（RCT）**

**Figures 2 Proportion of RoB2 risk of bias across domains（RCT）**

Note: RoBINS-I = Risk Of Bias In Non-randomized Studies - of Interventions; L = Low risk; M = Moderate risk; S = Serious risk; C = Critical risk; NI = No information. D1 = Bias due to confounding; D2 = Bias in selection of participants into the study; D3 = Bias in classification of interventions; D4 = Bias due to deviations from intended interventions; D5 = Bias due to missing data; D6 = Bias in measurement of outcomes; D7 = Bias in selection of the reported result.

**Figures 2 RoBINS-I Risk of Bias Judgments（RS）**

**Figures 3Proportion of RoBINS-I risk of bias across domains（RS）**

Figures 4 ORR Forest Plot by Chemotherapy Backbone (GemCis vs. Non-GemCis)

Figures 5 ORR Forest Plot by Study Design (Phase III vs. Single-Center)

Figures 6 OS Forest Plot by Geographic Region (China vs. Non-China)

Figures 7 OS Forest Plot by Tumor Subtype (Mixed BTC, iCCA, GBC)

**Figures 8 PFS Forest Plot by Study Design (Single-Center vs. Multicenter)**

Figures 9PFS Forest Plot by Geographic Region (China vs. Non-China)

Figures 10PFS Forest Plot by Tumor Subtype (Mixed BTC, iCCA, GBC)

Figures 8 DCR Funnel Plot

Figures 9 OS Funnel Plot (Retrospective Studies)

Figures 10 RCT sensitivity_DCR_loo

Figures 11 RCT sensitivity_ORR_loo

Figures 12 trimfill_OS

Figures 13 trimfill_PFS

Figures 14 bubble_PFS_samplesize
