# Supplementary material for: PD-1/PD-L1 inhibitors combined with chemotherapy as first-line treatment for advanced biliary tract cancer: a systematic review and meta-analysis
Source: Front Med (Lausanne). 2026 Sep 3;13:1916331. doi: 10.3389/fmed.2026.1916331 (PMC13581943; doi:10.3389/fmed.2026.1916331)
Supplement: Supplementary file 3 [file Data_Sheet_2.DOCX]

**Supplementary Appendix 1**

**AI-Assisted Content and Prompts**

*PD-1/PD-L1 Inhibitors Combined with Chemotherapy as First-Line
Treatment for Advanced Biliary Tract Cancer:
A Systematic Review and Meta-Analysis*

**1. AI Tools and Versions**

This study utilized generative AI tools for specific tasks during manuscript preparation. All AI-generated content was reviewed, validated, and revised by the authors, who take full responsibility for the final content of the manuscript.

| **Tool Name** | **Version / Model** | **Developer** |
| --- | --- | --- |
| Kimi (Moonshot AI) | Kimi K2.6 | Moonshot AI (Beijing, China) |
|  |  |  |

**2. Areas of AI Assistance**

AI was used in the following specific areas during manuscript preparation and revision. The authors designed all research questions, analytical frameworks, and clinical interpretations. AI served solely as an auxiliary tool for language, formatting, and coding support.

**2.1 Language Editing and Polishing**

AI was used to assist with English language polishing, grammar correction, and stylistic improvement of the manuscript text. This included:
• Correcting spelling errors and typographical mistakes throughout the manuscript
• Improving sentence structure and academic writing style
• Ensuring consistent terminology across all sections
• Refining the abstract and discussion sections for clarity and conciseness

**2.2 R Code Generation and Statistical Support**

AI was used to assist with R code development for meta-analyses and figure generation. Specific tasks included:
• Generating initial code templates for meta-analysis using the meta and metafor packages
• Assisting with forest plot customization and layout optimization
• Debugging code errors and suggesting improvements for figure aesthetics
• Helping with code organization and documentation

All statistical analyses were designed, executed, and validated by the authors. The authors verified all numerical results and interpret all findings.

**2.3 Reference Formatting**

AI was used to assist with formatting references according to Frontiers in Medicine journal guidelines. This included standardizing reference style, correcting formatting inconsistencies, and ensuring proper citation format. All references were manually verified by the authors for accuracy and completeness.

**2.4 Response to Reviewers Drafting**

During the revision process, AI was used to assist with drafting the point-by-point response letter to reviewers. This included helping organize responses, suggest appropriate phrasing, and ensure comprehensive coverage of all reviewer comments. All responses were reviewed, revised, and approved by the authors.

**2.5 Manuscript Revision Support**

AI assisted with various revision tasks, including:
• Suggesting standardized phrasing for methodological clarifications
• Helping organize supplementary material descriptions
• Assisting with consistency checks across manuscript sections
• Generating template text for results descriptions (which were then validated against actual data)

**3. Author Verification and Validation Process**

All AI-generated content underwent rigorous review and validation by the authors. The verification process included:

**(1) Statistical Results:** All numerical results, effect sizes, confidence intervals, p-values, and heterogeneity statistics were independently verified by the authors by running the R code manually and cross-checking with the original study publications.

**(2) Clinical Interpretation:** All clinical interpretations and conclusions were developed and validated by the authors based on their expertise in biliary tract cancer oncology and meta-analysis methodology.

**(3) Factual Accuracy:** All factual statements, study characteristics, and references were manually verified against the original source publications.

**(4) Scientific Content:** The scientific content, study design, and analytical approach were entirely conceived and executed by the authors. AI played no role in study design, data extraction, or scientific interpretation.

**(5) Language Quality:** All AI-polished text was reviewed for scientific accuracy, appropriate tone, and correct use of medical terminology.

**4. AI-Generated Content Disclaimer**

The authors declare that generative AI tools were used solely for language assistance, coding support, and formatting tasks. AI was not used to generate scientific content, interpret data, or draw conclusions. The authors take full responsibility for the entire content of this manuscript, including all AI-assisted portions that have been reviewed and validated by the authors.

No AI tool is listed as an author. The use of AI does not constitute authorship, and all authorship criteria have been met exclusively by the human authors listed on the manuscript.

**5. Prompts Used**

Below are representative actual prompts provided to the AI tool during manuscript preparation and revision. These prompts reflect the specific tasks performed with AI assistance. All AI outputs were reviewed, edited, and validated by the authors before incorporation into the manuscript.

**5.1 Manuscript Revision and Language Editing Prompts**

***Prompt 1: Abstract Rewriting***

*"Please rewrite the abstract of this meta-analysis manuscript. The key changes needed are: (1) clarify that Part I includes 5 RCTs with 2,000 patients, but only 2 phase III RCTs (TOPAZ-1 and KEYNOTE-966, n=1,754) reported OS and PFS data, while all 5 RCTs reported ORR and DCR; (2) unify the R version to 4.6.1 throughout; (3) add a sentence stating that full R code and datasets are provided in the Supplementary Material for reproducibility; (4) Part II should consistently state 6 retrospective studies with 1,253 patients. Keep the abstract within 250 words and maintain the standard structured abstract format."*

***Prompt 2: GBC Subgroup Discussion Tone Adjustment***

*"Please revise the discussion section regarding gallbladder cancer (GBC) subgroup findings. The current text is too optimistic about GBC benefit. Please: (1) acknowledge honestly that in the two phase III RCTs, the GBC subgroup did not show statistically significant OS benefit (TOPAZ-1 HR 0.90, KEYNOTE-966 HR 0.96); (2) state that the positive GBC results from retrospective studies should be interpreted cautiously; (3) suggest possible explanations including selection bias and baseline prognostic factor differences; (4) conclude that the GBC subgroup finding should be considered hypothesis-generating pending further prospective validation. Maintain a balanced, evidence-based tone without overstating the benefit."*

***Prompt 3: Spelling and Consistency Corrections***

*"Please go through the entire manuscript and: (1) correct all spelling errors, including cmhnsive→comprehensive, radmized contolld tils→randomized controlled trials, manutripts→manuscripts; (2) replace all instances of R 4.3 with R 4.6.1 for consistency; (3) resolve the inconsistency in the number of retrospective studies—some places say 5, some say 6, please unify to 6 throughout; (4) standardize the AI usage statement across the cover page, acknowledgments, and supplementary material sections. List all changes you make so I can verify them."*

**5.2 Statistical Analysis and R Code Support Prompts**

***Prompt 4: PFS Fixed-Effect Model Re-analysis***

*"I need to re-run the PFS meta-analysis for the RCT component using a fixed-effect model instead of random-effects. The reason is that I² = 29.8% (p = 0.23), which is below the 50% threshold specified in our predefined protocol. The two studies are: Oh 2022 TOPAZ-1 (HR 0.75, 95% CI 0.63-0.89) and Kelley 2023 KEYNOTE-966 (HR 0.86, 95% CI 0.75-1.00). Please write R code using the meta package to: (1) calculate the pooled HR using fixed-effect model; (2) generate a forest plot with JAMA layout; (3) report I², Q statistic, and p-value for heterogeneity; (4) save as both PNG (300 dpi) and PDF. Use dark blue for the diamond and square colors."*

***Prompt 5: OS Subgroup Forest Plot by Tumor Type***

*"Please generate a forest plot for OS subgroup analysis by tumor type from the retrospective studies. The subgroups are: mixed BTC (HR 0.64, 95% CI 0.34-1.19, from Zhao 2021, Lu 2024, Madzikatire 2024, Rimini 2024, Zhao 2025), iCCA (HR 0.53, 95% CI 0.30-0.94, from Madzikatire 2024), and GBC (HR 0.53, 95% CI 0.36-0.79, from Zhang 2025). Use the meta package metagen function. The forest plot should show each subgroup separately with pooled estimates, include heterogeneity statistics within each subgroup, and add an interaction test p-value at the bottom (p = 0.88). Use red color scheme for OS."*

***Prompt 6: Cohort Overlap Sensitivity Analysis***

*"I need to create a sensitivity analysis figure assessing the impact of potential patient cohort overlap between Zhao 2021 and Zhao 2025 (both from the same center in China). Please create a figure showing 4 scenarios for both PFS and OS: (1) all 6 studies included; (2) excluding Zhao 2021; (3) excluding Zhao 2025; (4) excluding both Zhao studies. The PFS HRs are: all 0.52 (0.43-0.63), exclude Zhao2021 0.51, exclude Zhao2025 0.55, exclude both 0.53. The OS HRs are: all 0.61 (0.42-0.87), exclude Zhao2021 0.57, exclude Zhao2025 0.67 (0.50-0.90), exclude both 0.64. Use ggplot2 to create a clean forest-plot-style figure with both PFS and OS side by side or stacked. Add a note at the bottom explaining the overlap concern."*

**5.3 Response to Reviewers Drafting Prompts**

***Prompt 7: Reviewer 1 - Study Classification and Patient Overlap***

*"Please help me draft a response to Reviewer 1 Major Comment 3 about study classification and potential patient overlap. The reviewer points out that: (1) Rimini et al. 2024 is included as a retrospective study but also appears in the discussion as validation of TOPAZ-1 results, making its classification unclear; (2) Zhao 2021 and Zhao 2025 are both from the same Chinese center, potentially having overlapping patient cohorts. Our response should: (1) clearly state that Rimini 2024 is exclusively a retrospective real-world study and was never included in the RCT meta-analysis; (2) explain that we verified through original publications that Zhao 2021 and Zhao 2025 had non-overlapping enrollment periods; (3) describe the sensitivity analyses we performed (excluding each Zhao study individually and both simultaneously); (4) report the results showing conclusions remain robust. Be polite, thorough, and specific."*

***Prompt 8: Reviewer 3 - ICI Heterogeneity Question***

*"Please draft a response to Reviewer 3 Comment 4 about ICI drug heterogeneity. The reviewer questions whether all PD-1/PD-L1 inhibitors are interchangeable, noting that some studies use nivolumab (not yet approved for BTC) and others use various drugs. Our response should: (1) acknowledge this is an important point; (2) list all ICI agents included in the analysis; (3) describe the subgroup analysis we added by ICI type (PD-1 vs PD-L1) with interaction test results (PFS p=0.11, OS p=0.18); (4) clearly state that we do NOT claim equivalence across all PD-1/PD-L1 inhibitors; (5) clarify that our conclusion is restricted to the class effect observed in available evidence; (6) distinguish between approved agents (durvalumab, pembrolizumab) and exploratory agents. Keep the tone constructive and evidence-based."*

**5.4 Methodological Description and Results Reporting Prompts**

***Prompt 9: Limitations Section Expansion***

*"Please help expand the limitations section of our discussion. Currently we have 5 limitations. Please add a 6th limitation about potential patient cohort overlap: Zhao 2021 and Zhao 2025 are from the same center, which raises the possibility of overlapping patient populations. We performed sensitivity analyses excluding these studies individually and together, and the overall conclusions remain robust, though excluding Zhao 2025 resulted in modest attenuation of the OS effect (HR 0.67 vs 0.61). Also, please review and refine all existing limitations to ensure they are clearly stated and appropriately balanced. The limitations should be: (1) RCT evidence relies on only 2 phase III trials; (2) retrospective studies have inherent selection bias; (3) ICI agents are heterogeneous; (4) GBC subgroup evidence is limited; (5) limited number of studies for some outcomes; (6) potential cohort overlap between Zhao studies."*

***Prompt 10: OS Subgroup Results Description***

*"Please generate the results section text describing the OS subgroup analysis by tumor type from the retrospective studies. The data is: mixed BTC subgroup (5 studies): HR 0.64 (95% CI 0.34-1.19); iCCA subgroup (1 study): HR 0.53 (95% CI 0.30-0.94); GBC subgroup (1 study): HR 0.53 (95% CI 0.36-0.79). The interaction test p-value is 0.88, indicating no statistically significant difference between subgroups. Please write this in standard meta-analysis results reporting style, include HR, 95% CI, mention the interaction test, and keep it concise (about 80-100 words). Use past tense and formal academic language."*

**5.5 Supplementary Material Preparation Prompts**

***Prompt 11: R Code Organization for Supplementary Material***

*"Please help organize our R code for submission as supplementary material to Frontiers in Medicine. We have 3 R script files: (1) 5RCTmeta-analysis.R - meta-analysis of 5 RCTs; (2) 6个PSmeta-analysis.R - meta-analysis of 6 retrospective studies; (3) reviewer_revised_figures.R - revised figures for reviewer response. Please: (1) rename them to English names following Frontiers conventions; (2) add standardized file headers with manuscript title, file purpose, R version, package versions, and corresponding manuscript sections; (3) translate all Chinese comments to English; (4) create a README file explaining each script, how to run them, and what output they produce; (5) ensure all study data is embedded in the scripts for full reproducibility."*

*Note: The above prompts are representative examples of the actual prompts used during manuscript preparation and revision. Many iterative refinements, follow-up prompts, and conversational adjustments were used throughout the process. All AI outputs were subject to critical review, editing, and validation by the authors before incorporation into the manuscript. The authors take full responsibility for all content.*

**6. Author Confirmation**

The corresponding author confirms on behalf of all authors that:

• All AI-assisted content has been reviewed and approved by the authors.
• The authors take full responsibility for the accuracy and integrity of all content.
• AI tools were not used to generate scientific conclusions or interpret data.
• No AI tool meets the criteria for authorship.
• All listed authors meet the ICMJE authorship criteria.
