# Supplementary material for: PD-1/PD-L1 inhibitors combined with chemotherapy as first-line treatment for advanced biliary tract cancer: a systematic review and meta-analysis
Source: Front Med (Lausanne). 2026 Sep 3;13:1916331. doi: 10.3389/fmed.2026.1916331 (PMC13581943; doi:10.3389/fmed.2026.1916331)
Supplement: Supplementary file 4 [file Data_Sheet_3.DOCX]

**Supplementary Figures**

*PD-1/PD-L1 Inhibitors Combined with Chemotherapy as First-Line
Treatment for Advanced Biliary Tract Cancer:
A Systematic Review and Meta-Analysis*

**Supplementary Figure 1. RCT PFS Forest Plot (Fixed-Effect Model)**

Revised forest plot for progression-free survival (PFS) in the RCT component, using the fixed-effect model as specified in the predefined protocol (I² = 29.8%, p = 0.23, below the 50% threshold). This figure addresses Reviewer 1's comment regarding the inconsistency between the reported model selection and the predefined threshold.

*Addresses reviewer comment: Reviewer 1, Major Comment 2*

**Supplementary Figure 2a. PFS Subgroup Analysis by Tumor Type**

Subgroup analysis of progression-free survival (PFS) by tumor type (mixed BTC, iCCA, GBC) in the retrospective study component. Interaction test for subgroup differences: p = 0.34.

*Addresses reviewer comment: Reviewer 1, Major Comment 4*

**Supplementary Figure 2b. OS Subgroup Analysis by Tumor Type**

Subgroup analysis of overall survival (OS) by tumor type (mixed BTC, iCCA, GBC) in the retrospective study component. This figure was added in response to Reviewer 1's comment that OS subgroup results lacked visual representation. Interaction test for subgroup differences: p = 0.88.

*Addresses reviewer comment: Reviewer 1, Major Comment 4*

**Supplementary Figure 3. Cohort Overlap Sensitivity Analysis**

Sensitivity analysis assessing the impact of potential patient cohort overlap between Zhao 2021 and Zhao 2025 (both from the same center in China). Four scenarios are shown: (1) all 6 studies, (2) excluding Zhao 2021, (3) excluding Zhao 2025, (4) excluding both Zhao studies. Results remain robust across all scenarios.

*Addresses reviewer comment: Reviewer 1, Major Comment 3*

**Supplementary Figure 4a. PFS Subgroup Analysis by ICI Type**

Subgroup analysis of progression-free survival (PFS) by type of immune checkpoint inhibitor (PD-1 vs PD-L1) in the retrospective study component. This figure addresses Reviewer 3's question regarding the heterogeneity of ICI agents across studies. Interaction test for subgroup differences: p = 0.11.

*Addresses reviewer comment: Reviewer 3, Comment 4*

**Supplementary Figure 4b. OS Subgroup Analysis by ICI Type**

Subgroup analysis of overall survival (OS) by type of immune checkpoint inhibitor (PD-1 vs PD-L1) in the retrospective study component. This figure addresses Reviewer 3's question regarding the heterogeneity of ICI agents across studies. Interaction test for subgroup differences: p = 0.18.

*Addresses reviewer comment: Reviewer 3, Comment 4*
